# 3D Anomaly Detection for Sensor-Based Industrial Inspection: A Critical Survey of Methods, Benchmarks, and Deployment Constraints

**DOI:** 10.3390/s26216717

**Published:** 2026-10-23

**Authors:** Lars Fichtel, Radu Timofte, Dominik Seuß

**Affiliations:** 1 Center for Artificial Intelligence (CAIRO), Technical University of Applied Sciences Würzburg-Schweinfurt, Münzstraße 12, 97070 Würzburg, Germany; 2 Computer Vision Laboratory, University of Würzburg, 97074 Würzburg, Germany; 3 Fraunhofer Institute for Integrated Circuits IIS, 91058 Erlangen, Germany

**Keywords:** 3D anomaly detection, industrial inspection, point clouds, RGB-D sensing, multimodal sensor fusion, unsupervised learning, few-shot learning, zero-shot learning, benchmark datasets, 3D sensing

## Abstract

Some industrial defects are geometric and invisible to a color camera, and anomaly detection on point clouds, depth maps and RGB-D images grew up around them. This survey covers 2020 to 21 August 2026, 94 detectors and 17 benchmark datasets, with an executable search protocol tested against the corpus and every collated number attributed to its source. Methods sit on two axes: six mutually exclusive scoring mechanisms (feature and memory matching, reconstruction, teacher–student discrepancy, density estimation, registration, and foundation-model transfer) and eight separable design dimensions, so fusion and zero-shot supervision are properties, not families. Benchmark results are collated with protocol, metric variant and peer-review status rather than ranked: on MVTec 3D-AD, the highest image-level area under the receiver operating characteristic curve (I-AUROC) is 98.4% peer-reviewed and 97.2% in a preprint, under differing protocols; the five highest span 1.1 points. Color-and-geometry fusion leads where both are captured; density estimation and reconstruction lead on point-cloud-only, few-shot Real3D-AD. Few of the 108 works examined report enough of the fields a runtime comparison needs. Sensing robustness is mostly tested along conditions an ablation already varies, rarely along those an instrument would introduce. A closing guide states what the evidence lets a practitioner select.

## 1. Introduction

Anomaly detection, the identification of samples or regions that deviate from expected normal patterns, is a fundamental problem in computer vision with critical applications in industrial quality control. Two-dimensional (2D) anomaly detection methods achieve strong performance on established benchmarks [1,2,3,4] but analyze surface appearance from a single viewpoint, so geometric defects such as warping, denting, and swelling are difficult or impossible to detect from 2D images alone and are visible in the full three-dimensional geometry of the object.

Cheaper, more available 3D sensing hardware, such as structured-light scanners and LiDAR, creates both the opportunity and the demand for anomaly detection directly on 3D data. Sub-millimeter point spacing is now routine, and point spacing bounds what a scan can represent, not what a detector can find or what a tolerance decision can rest on (Section 11).

### 1.1. Motivation and Scope

This survey reviews methods, benchmarks and deployment constraints for 3D anomaly detection from 2020 to 2026, covering learned unsupervised, few-shot, and zero-shot methods over point clouds, depth maps, RGB-D images, or multi-view reconstructions, and both detection (whether a sample is anomalous) and localization (which regions are defective).

**Figure 1:**
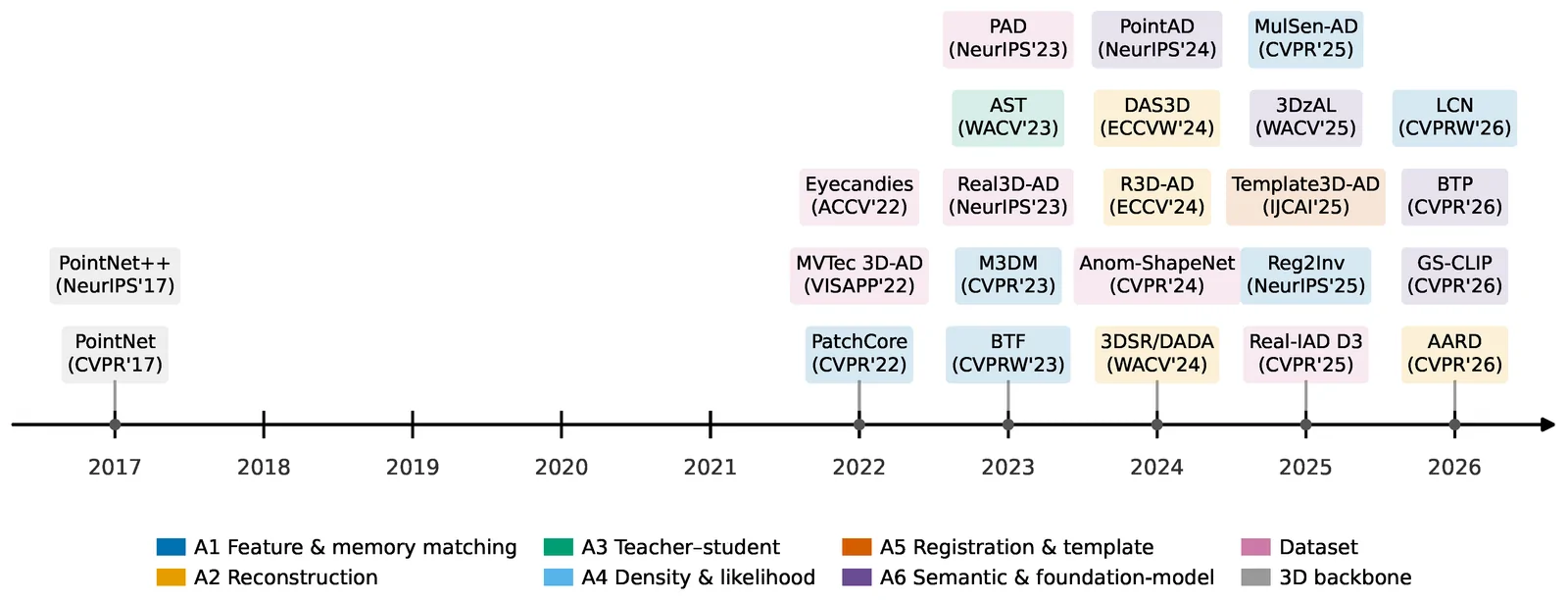
Timeline of key publications in 3D anomaly detection (2017–2026), showing the progression from foundational 3D representations and 2D anomaly detection methods to dedicated 3D approaches and emerging paradigms.

The field has grown rapidly since the MVTec 3D-AD benchmark appeared in 2022 [5] (Figure 1).

### 1.2. Survey Scope and Methodology

The protocol was written alongside the corpus rather than before it and was not registered in advance. We therefore make no claim of conformance to a systematic review reporting standard.

The search covers arXiv and OpenAlex, using 16 phrase queries over title and abstract, restricted to work from 2020 onward, and frozen at 21 August 2026, when both sources were last queried in one run. We searched OpenAlex rather than the longer list such reviews often name because it indexes Crossref, arXiv, and the major publishers and returns an exact result count for a query anyone can re-issue. arXiv is searched separately because this field’s preprint layer often precedes indexing elsewhere.

No query contains a method-specific term. Expanding a search with the names of the paradigms one expects to find (e.g., “memory bank” and “normalizing flow”) can only rediscover those paradigms, and a taxonomy confirmed that way confirms itself. Every query names a task, a modality, or a supervision setting. Table S1 of the Supplementary Materials lists the phrases with what each returned; Section S6 there gives the executed strings, and Table 1 summarizes the search by tier. Because a database search alone proved insufficient, citation chaining through the reference lists of included works and through works citing them is treated as a required stage.

We retained a paper when it (i) proposes or evaluates a machine learning method for anomaly detection on 3D data in object-level inspection settings, (ii) is peer-reviewed or deposited on arXiv with substantial technical content, and (iii) reports quantitative results on at least one established benchmark. We excluded purely 2D methods, non-learning approaches, application papers without a methodological contribution, and work on volumetric medical imaging or large-scale LiDAR scenes, whose data regimes and evaluation protocols differ fundamentally; Section 14 returns to them briefly. Multi-view pipelines over rendered or captured views of a 3D object are in scope since object geometry rather than a single view drives the detection. The queries returned 2937 records; de-duplication on arXiv identifier, DOI, and exact folded title removed 1211, leaving 1726 unique. Title-and-abstract screening excluded 734 under three recorded rules, leaving 992 for eligibility assessment. Of those, 121 name an established benchmark in title or abstract, and 871 do not; with the 317 records added by citation chaining, 1188 reached the full-text stage, of which 211 could be retrieved and assessed. The remainder stay at an abstract level, and Section S6 of the Supplementary Materials reports the cost of that limit. Table S5 of the Supplementary Materials lists every record whose full text was retrieved and then failed criterion (iii), so the full-text stage is accounted for record by record.

Four properties were measured, rather than assumed, against the corpus this survey had already assembled by expert reading: recovery (93 of the 102 inventory works, 91%, not uniformly held out; 26 of 32 on the unfitted subset, Section S6), what citation chaining adds, screening sensitivity, and the cost of the abstract-level proxy. Section S6 of the Supplementary Materials reports all four and the single-reviewer limitation in full.

For each retained method, we extracted its paradigm, input modality, supervision regime, venue, peer-review status, and all reported benchmark metrics. Every benchmark number we report was read from the originating publication and released with its location, one row per value in Table S8 of the Supplementary Materials. Section 13 collates the values. Three quantities are reported separately, because conflating them is how a flow diagram comes to state a total that partitions nothing. *Records*: 1726 unique remain of the 2937 retrieved. *Reports*: 113 publications are included. *Entities*: those publications yield 94 dedicated 3D anomaly detection methods, 8 related techniques, and 17 benchmark datasets. The 94 detectors and 8 related techniques are the 102 works of the inventory; adding the 17 dataset papers gives 119 entity-contributing works; and 6 of those contribute both a method and a dataset, so the count of distinct publications is 119 minus 6, which is 113. The entity counts, therefore, do not partition the included reports, and the identity is asserted by a consistency check whose result is recorded with the search log. The full inventory is Table S4 of the Supplementary Materials, and the extraction schema behind it is Table S3 there.

The 8 related techniques, included for context, contribute no anomaly-scoring mechanism and are not counted among the 94; Table S4 lists them separately. Preprints are flagged explicitly (venue “arXiv” in tables, *Pre.* in Table S4), and when they report results on an established benchmark, they appear in the narrative and the comparison tables of Section 13 as a separate evidence tier. No preprint result is described as establishing a state of the art.

### 1.3. Comparison with Existing Surveys

Existing surveys cover 2D industrial or self-supervised anomaly detection [6,7,8], RGB, 3D and multi-modal unsupervised industrial anomaly detection [9], and point-cloud and 3D-vision defect detection from an application standpoint [10,11]. Liang et al. [12] present a regularly updated preprint review of 3D anomaly detection with a taxonomy focused on method classification, the closest point of comparison and, as a living document with an associated leaderboard list, a moving target rather than a fixed reference. Table 2 positions this survey against it and the others.

Three additions relative to Liang et al. are concrete rather than a claim of greater recency. First, a sustained *2D-to-3D paradigm-transfer analysis* (Section 2): for each paradigm we identify the 2D anomaly detection idea it descends from or establish that it has none and assess whether the transfer was effective (Table 3). Second, registration and alignment are a *first-class paradigm* (Section 8) rather than an “other” bin, reflecting their rapid 2025–2026 growth. Third, our cross-benchmark tables are *source-verified* against the originating publications. We deliberately do *not* claim a “unified evaluation”; Section 13.2, Section 13.3 and Section 13.4 collate numbers reported under heterogeneous protocols, so they rank benchmarks more reliably than methods. Against the 2D-focused surveys [6,7,8] and the application-oriented 3D reviews [10,11], our organization by algorithmic paradigm and our coverage of the 2025–2026 foundation-model and multi-category wave provide a methodology-first entry point.

### 1.4. Contributions

This survey argues that 3D anomaly detection is not a straightforward extension of 2D industrial anomaly detection. Many 3D methods do inherit their algorithmic templates from 2D, memory banks and reconstruction models among them, but their success turns on factors that are absent or secondary in 2D: geometric correspondence, pose variation, sensor modality, and the availability of meaningful 3D pretraining. Registration and template deviation are natively 3D, and the continued competitiveness of handcrafted geometric descriptors indicates that learned 3D representations have not displaced classical geometry. We therefore analyze which methods perform well and the benchmark and sensing conditions under which their assumptions hold.

The contributions are:

1. A two-axis taxonomy (Section 3) that separates the primary anomaly-scoring mechanism, of which a method has exactly one, from the eight design dimensions that are recorded separately from it, which removes the arbitrary placement single-axis taxonomies force and keeps the 2D-to-3D paradigm-transfer lens on the mechanism axis.
2. Coverage of all 94 methods, charted on both axes and enumerated with venue and peer-review status in Table S4 of the Supplementary Materials, with detailed technical analysis for the representative methods of each mechanism (Section 4, Section 5, Section 6, Section 7, Section 8 and Section 9) and the 25 extended descriptions in Section S10 there, 16 of them for works the article names once and does not use again.
3. A review of 17 benchmark datasets and standardized evaluation metrics, including the most recent from 2025–2026 (Section 12).
4. A consolidated, source-verified experimental comparison across methods and benchmarks, reported under heterogeneous protocols rather than a unified re-evaluation (Section 13).
5. Open challenges and research directions, including scalability, foundation model integration, and zero-shot detection (Section 15).

## 2. Background and Preliminaries

Two facts drive most design decisions here: the representations are not interchangeable (Section 2.1), and the available pretrained 3D backbones are much weaker than their 2D counterparts (Section 2.4).

### 2.1. 3D Data Representations

The format constrains the architecture more than it constrains the method.

#### 2.1.1. Point Clouds

A point cloud $P={\left\{p_{i}\right\}}_{i=1}^{N}$ is an unordered set of *N* points, each $p_{i}\in R^{3+C}$ carrying coordinates (x,y,z) and optionally *C* attributes. Being unordered, it requires permutation-invariant architectures such as PointNet [13] and PointNet++ [14].

#### 2.1.2. RGB-D Images

RGB-D pairs a color image with a per-pixel depth map on a regular grid, which a 2D CNN reads directly. MVTec 3D-AD [5] ships in this form, as paired RGB and organized point clouds readable as depth maps. The comparison tables abbreviate input modality as PC (point cloud only), RGB-D, and RGB+PC (color image with an unstructured point cloud) and use RGB+3D synonymously with RGB+PC.

#### 2.1.3. Voxel Grids, Meshes, and Multi-View Images

Voxel grids discretize space into a regular volume at a memory cost cubic in resolution; sparse variants [15] soften that, and ModelNet [16] is the standard volumetric benchmark. Triangular meshes give an explicit surface with an irregular topology. No method surveyed here is recorded as operating on either format in the released inventory, and the one benchmark that ships meshes, Anomaly-ShapeNet, is consumed as point clouds. Multi-view representations image an object from several camera positions, which CPMF [17] exploits by rendering point clouds into views.

### 2.2. Problem Formulation

Let X denote the input space of 3D data representations and Y={0,1} the label space, with y=0 normal and y=1 anomalous. The goal is to learn a scoring function s:X→R low for normal samples and high for anomalous ones.

The supervision regimes differ in the content of the training set $D_{\mathrm{train}}$. Four appear in the literature covered here:

- **One-class.** $D_{\mathrm{train}}={\left\{x_{i}\right\}}_{i=1}^{M}$ with $y_{i}=0$ for all *i*, so no anomalous example is seen in training. This is the predominant setting.
- **Synthetic-anomaly supervision.** $D_{\mathrm{train}}={\left\{x_{i}\right\}}_{i=1}^{M}\cup \left\{\left({\tilde{x}}_{i},m_{i}\right)\right\}$, where ${\tilde{x}}_{i}=\tau \left(x_{i}\right)$ comes from a normal sample through a corruption operator τ and $m_{i}\in {\left\{0,1\right\}}^{N_{x}}$ is the point mask it induces. No real defect is observed, so performance is bounded by how well τ covers the real defect distribution, and labels are exact by construction.
- **Open-set supervised.** $D_{\mathrm{train}}$ contains a few genuinely anomalous samples with $y_{i}=1$, drawn from a defect vocabulary known to be incomplete.
- **Few-shot and zero-shot transfer.** A model ψ pretrained on source categories $C_{\mathrm{src}}$ is applied to a target category $c\notin C_{\mathrm{src}}$ given *k* normal references (*k* small, and k=0 in the zero-shot case). Nothing category-specific is fitted, and the reference set is consumed at inference.

These are supervision regimes, not method families, and the same mechanism appears in several of them, which is why Section 3 treats supervision as a cross-cutting dimension.

For anomaly *localization*, the objective extends to a point-wise or pixel-wise anomaly map $a:X\to R^{N_{x}}$ over the $N_{x}$ points or pixels of *x*, with *j*-th component $a_{j}\equiv {\left[a\left(x\right)\right]}_{j}$. We use $a_{j}$ throughout the method sections and aggregate it for the image-level score s(x) (e.g., $s\left(x\right)=\max_{j}a_{j}$).

#### Category Scope

Orthogonal to the supervision regime is how many object categories one fitted model covers:

- **One-class-one-model**: A separate model per object category, the standard protocol in most benchmarks.
- **Unified/multi-category**: A single model handles multiple categories, as explored by MC3D-AD [18].

Real3D-AD [19] is few-shot and one-class-one-model; PointAD [20] is zero-shot and category-agnostic, with no per-category fit.

### 2.3. From 2D to 3D Anomaly Detection

3D anomaly detection builds on 2D industrial anomaly detection, and we assess each paradigm against its 2D ancestor (Table 3).

#### 2.3.1. Feature Embedding Methods

PatchCore [2] stores coreset-subsampled patch features from a pretrained CNN and scores by nearest-neighbor distance; SPADE [21] scores by *k*NN over a feature pyramid; and PaDiM [3] by a per-position Gaussian. These paradigms are adopted directly in M3DM [22] and BTF [23]. SimpleNet [24] trains a discriminator on perturbed pretrained features instead of a memory bank, and UniAD [25] handles multiple classes with one transformer, later mirrored in 3D by MC3D-AD [18].

#### 2.3.2. Teacher–Student Methods

STFPM [26] trains a student to match multi-scale feature pyramids of a pretrained teacher on normal data and scores anomalies by feature discrepancy. Reverse Distillation [27] inverts this architecture with a pretrained teacher encoder and a trainable student decoder. EfficientAD [28] reaches roughly 2 ms per image with a lightweight patch description network. AST [29] carries the idea into 3D, pairing a normalizing-flow teacher with a feed-forward student.

#### 2.3.3. Normalizing Flow Methods

CFlow-AD [30], FastFlow [31], and CS-Flow [32] model the density of normal features from pretrained backbones with normalizing flows, scoring anomalies by low likelihood. Dedicated 3D instances are few: HGAD [33] builds a flow for 3D feature spaces, while VAE3D [34] pursues the same density-based scoring with a variational autoencoder (Section 7).

#### 2.3.4. Self-Supervised and Synthetic Anomaly Methods

CutPaste [35] generates synthetic anomalies by cutting and pasting image patches, and DRAEM [4] overlays external textures instead. In 3D the perturbation must deform geometry instead of overlaying texture, which R3D-AD [36] and DAS3D [37] solve in different ways (Section 3.2).

#### 2.3.5. Paradigms Without a Direct 2D Ancestor

Three parts of the taxonomy do not descend from a single 2D anomaly detection idea. Multi-modal fusion (Section 10) has no ancestor in the 2D anomaly-detection literature specifically; multispectral, thermal and X-ray inspection are long-established in 2D industrial imaging. Registration and alignment (Section 8) derive from classical geometric vision. Foundation-model and zero-shot methods (Section 9) descend from 2D vision–language detectors such as AnomalyCLIP [38] and AnomalyGPT [39].

### 2.4. 3D Feature Learning Backbones

#### 2.4.1. Handcrafted Descriptors

Fast Point Feature Histograms (FPFH) [40] compute rotation-invariant descriptors from surface normals and Darboux frames; Section 4 sets out under which benchmark and modality conditions they hold up against learned 3D representations, BTF [23] being the case in point.

#### 2.4.2. Learned Representations

Five backbones account for nearly all learned 3D features in this survey. PointNet and PointNet++ [13,14] process point sets directly, the latter hierarchically. DGCNN [41] builds a *k*-nearest-neighbor graph and recomputes it in feature space at every layer. Point-BERT [42] and Point-MAE [43] bring masked pretraining to point clouds, with Point-MAE at masking ratios of 60–90%. Point Transformer [44] adapts self-attention [45] to point neighborhoods.

### 2.5. Evaluation Metrics

Evaluation covers *detection* (image- or object-level) and *localization* (pixel- or point-level), reported in Section 13 and summarized in Table 4.

**I-AUROC**, the image-level area under the receiver operating characteristic curve, is the probability that a randomly chosen anomalous sample scores above a randomly chosen normal one. On object-centric benchmarks the same quantity is reported as object-level **O-AUROC**, and we follow each benchmark’s naming convention. It does not penalize poor localization, and reported values on the established benchmarks now sit above 95%, which limits what it separates. **P-AUROC** is the same integral computed point-wise against a ground-truth mask, biased toward large anomalous regions because every point counts equally.

**AUPRO**, the area under the per-region overlap curve, removes that size bias by scoring each ground-truth connected component independently:

(1) $$\mathrm{PRO}\left(\gamma \right)=\frac{1}{\left|R\right|}\sum_{R_{r}\in R}\frac{|R_{r}\cap \hat{A}\left(\gamma \right)|}{|R_{r}|},$$

where R is the set of ground-truth anomalous regions and $\hat{A}\left(\gamma \right)$ the points predicted anomalous at threshold γ. The area under the PRO curve is integrated to a false-positive-rate limit, typically 30%, and normalized to [0,1]. Recent benchmarks prefer it [5]. F1-max is the harmonic mean of precision and recall at its best threshold, average precision (AP) summarizes the precision–recall curve, and V-AUPRO extends AUPRO to voxels.

A connected component is unambiguous on a pixel grid, where 4- or 8-connectivity is given by the lattice, but a point cloud has no intrinsic neighborhood: R is only defined once a connectivity rule is fixed, by *k*-nearest neighbors, a fixed radius, a voxel adjacency, or mesh edges. The rule changes how defects split into components and so changes the denominator |R| and the reported AUPRO. Benchmarks and methods rarely state which rule they use, which is one reason point-level localization numbers are less comparable across papers than their metric name suggests.

## 3. A Two-Axis Taxonomy of 3D Anomaly Detection Methods

Method taxonomies in this field usually present one flat list of families that mixes kinds. “Multi-modal fusion” answers a question about *inputs*, “reconstruction” about *how an anomaly score is computed*, “zero-shot” about *what supervision was available*. Placing all three at one level forces a choice methods do not face, and a flat list must discuss a method that is either twice or place it arbitrarily.

**Figure 2:**
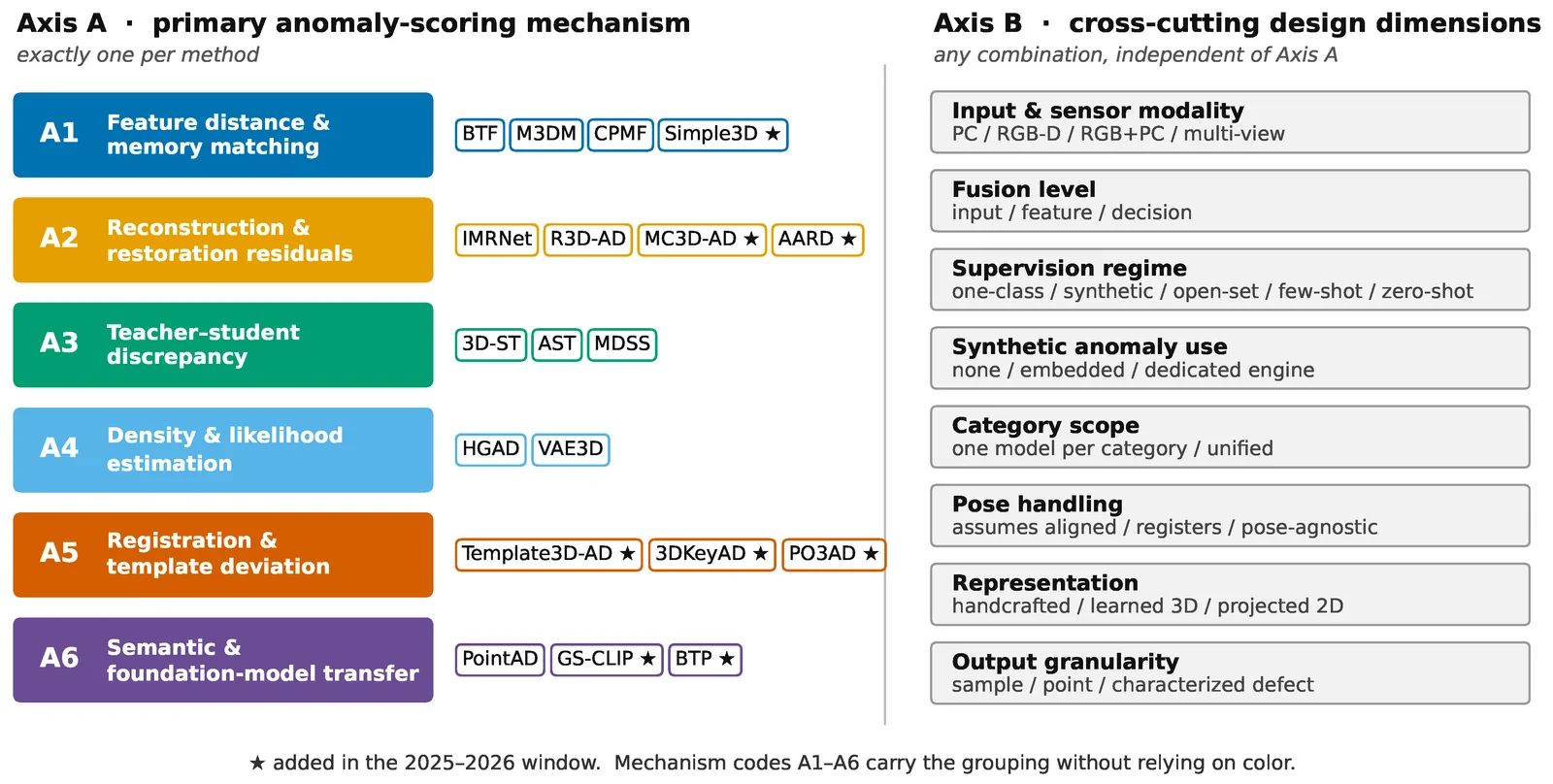
The two-axis taxonomy: six mutually exclusive scoring mechanisms (**left**) and eight independent design dimensions (**right**). Stars mark methods added in the 2025–2026 window. Representative methods only; the complete inventory is Table S4 of the Supplementary Materials.

We therefore organize the field along two axes. The first asks how a method turns an observation into an anomaly score, and a method has exactly one answer. The second records properties that vary independently of it. Figure 2 draws the scheme, and Table 5 the methods.

### 3.1. Axis A: Primary Anomaly-Scoring Mechanism

The mechanism producing a method’s score is mutually exclusive in a way that input modality and supervision are not, which makes it the primary axis. Six mechanisms cover the inventory.

#### 3.1.1. How a Method Is Assigned

The rule is operational: the primary mechanism is the one producing the quantity the method thresholds at test time. Two tie-breaks follow, in order. Cross-modal prediction scored by discrepancy is A3, and within-modality reconstruction scored by its residual is A2, since without that line the two are indistinguishable. Where two mechanisms are genuinely fused, the primary is the one the paper contributes rather than inherits, and Table 5 records the other as secondary. Every assignment came from the paper’s own statement of what produces its score; section membership would be circular, and a keyword tally was abandoned after it pointed at the wrong mechanism for a method fitting a multivariate Gaussian to fused features. Table S4 of the Supplementary Materials releases every assignment.

(A1) Feature distance and memory matching (Section 4).

Discriminative features come from pretrained backbones or handcrafted descriptors, a representative subset of normal features is stored, and the score is the distance from a test feature to its nearest stored neighbor (Equation (2)). Members include BTF [23], M3DM [22], Shape-Guided [46], and CPMF [17].

(A2) Reconstruction and restoration residuals (Section 5).

A model trained to reproduce normal data fails on anomalous regions, and the residual between the input and reconstruction is the score (Equation (3)). The family spans autoencoders and masked reconstruction [49], diffusion [36], and implicit neural representations [50].

(A3) Teacher–student and representation discrepancy (Section 6).

A pretrained teacher and a student trained only on normal data agree on normal input and diverge on anomalies, and the score is that divergence (Equation (4)). AST [29] adds architectural asymmetry to block the identity solution, and 3D-ST [51] distills deep geometric descriptors.

(A4) Density and likelihood estimation (Section 7).

The density of normal features is modeled explicitly, and low likelihood under that model is the score (Equation (6)). This is the sparsest of the six in 3D, with 5 members: HGAD [33], VAE3D [34], MVFM-3DAD [54], MMFlows [55], and 2M3DF [65]. The sparsity is itself a finding, given how well the same idea works in 2D [30,31,32], and a recent one: until MVFM-3DAD, no member reported on a standard 3D benchmark under a comparable protocol, a different situation from a paradigm tried and found wanting.

(A5) Registration and template deviation (Section 8).

A test sample is aligned to one or more normal templates and scored by geometric deviation from the aligned correspondence or by a predicted per-point offset back to the normal surface (Equation (7)). Alignment first appeared as preprocessing inside a memory bank method [19] and is a mechanism in its own right once the deviation carries the signal. PO3AD [59] predicts a displacement field rather than comparing against a template, and that field is thresholded like a reconstruction residual, so Table 5 places it here with reconstruction as its secondary mechanism.

(A6) Semantic and foundation-model transfer (Section 9).

A large pretrained vision or vision–language model defines normality rather than the target category, and the score is similarity in that model’s semantic space [20,60,66]. The mechanism is named for what it does, not for the supervision it enables; Section 9 sets out why transfer and zero-shot are separable.

#### 3.1.2. Exhaustiveness

The six positions were not fixed before reading; they are what the assignment produced. Each of the 94 detectors received exactly one primary mechanism (27, 25, 13, 5, 9, and 15 for A1 to A6), and the remaining 8 of the 102 inventory works are related techniques contributing no scoring mechanism. Testing the axis for a missing position found one work none of the six describes: BridgeNet [67] trains a dual-modal discriminator on synthesized anomalies with no reconstruction branch, so its score is a classifier output rather than a residual. Two mechanisms established in 2D are therefore represented here by at most one work each, one-class boundary methods by none, and discriminative heads by BridgeNet alone. One member is too few for a seventh position, so BridgeNet is recorded under A2 with the fit stated in its description (Section 10); should either class gain members, the axis gains a position, and until then the rule above places such work.

### 3.2. Axis B: Cross-Cutting Design Dimensions

The properties below vary independently of Axis A and may combine with any scoring mechanism, which is why none belongs on the primary axis.

- **Input and sensor modality.** Point cloud only, RGB-D, RGB plus point cloud, or multi-view renderings; the sensing conditions behind these labels are treated below.
- **Fusion level.** Where modalities are combined: raw input, feature space, or decision. Multi-modal methods only, detailed in Section 10, which treats fusion as this dimension rather than a paradigm.
- **Supervision regime.** One-class, synthetic-anomaly supervised, open-set supervised, few-shot, or zero-shot, formalized in Section 2.2, which also states why the two middle settings, the broader literature’s weak supervision, are kept apart.
- **Synthetic anomaly use.** A training-data strategy, not a paradigm: Patch-Gen in the diffusion-based R3D-AD [36], RGB and depth synthesis in DAS3D [37], depth simulation in 3DSR/DADA [48], each inside a method scoring by reconstruction. Dedicated synthesis engines now exist (3D-PNAS [68], the MLLM-guided Synthesis4AD [69]), and for that subject we refer to Wang et al. [70].
- **Category scope.** One model per category, the standard benchmark protocol, against a unified model covering many [18].
- **Pose handling and correspondence assumptions.** Whether the method assumes aligned input, aligns explicitly, or is pose-agnostic. It separates methods more sharply on Real3D-AD than the scoring mechanism does (Section 13).
- **Representation.** Handcrafted descriptors (FPFH [40]), learned 3D features (Point-MAE [43], Point-BERT [42]), or pretrained 2D features applied to projected views.
- **Output granularity.** Sample-level detection, point- or pixel-level localization, and whether the method characterizes the defect beyond locating it.

### 3.3. Reading the Two Axes Together

M3DM [22] is the clearest case. Its score is a nearest-neighbor distance in a memory bank, so its mechanism is A1, and it sits beside PatchCore-style methods there, while its fusion of RGB and point-cloud features places it beside CFM and DAS3D on the other axis. It is described once (Section 4) rather than twice. Registration-based methods show the same in reverse: heterogeneous by modality and one family by mechanism.

Five entries in Table 5 span mechanisms genuinely rather than apparently, namely Reg3D-AD, PO3AD, 3DKeyAD, AST and MISDD, the last of which substitutes a prompt for a sensor it does not have. Table 5 assigns each a primary mechanism and records the secondary one, because a category for every pair would produce a taxonomy with more categories than members.

**Figure 3:**
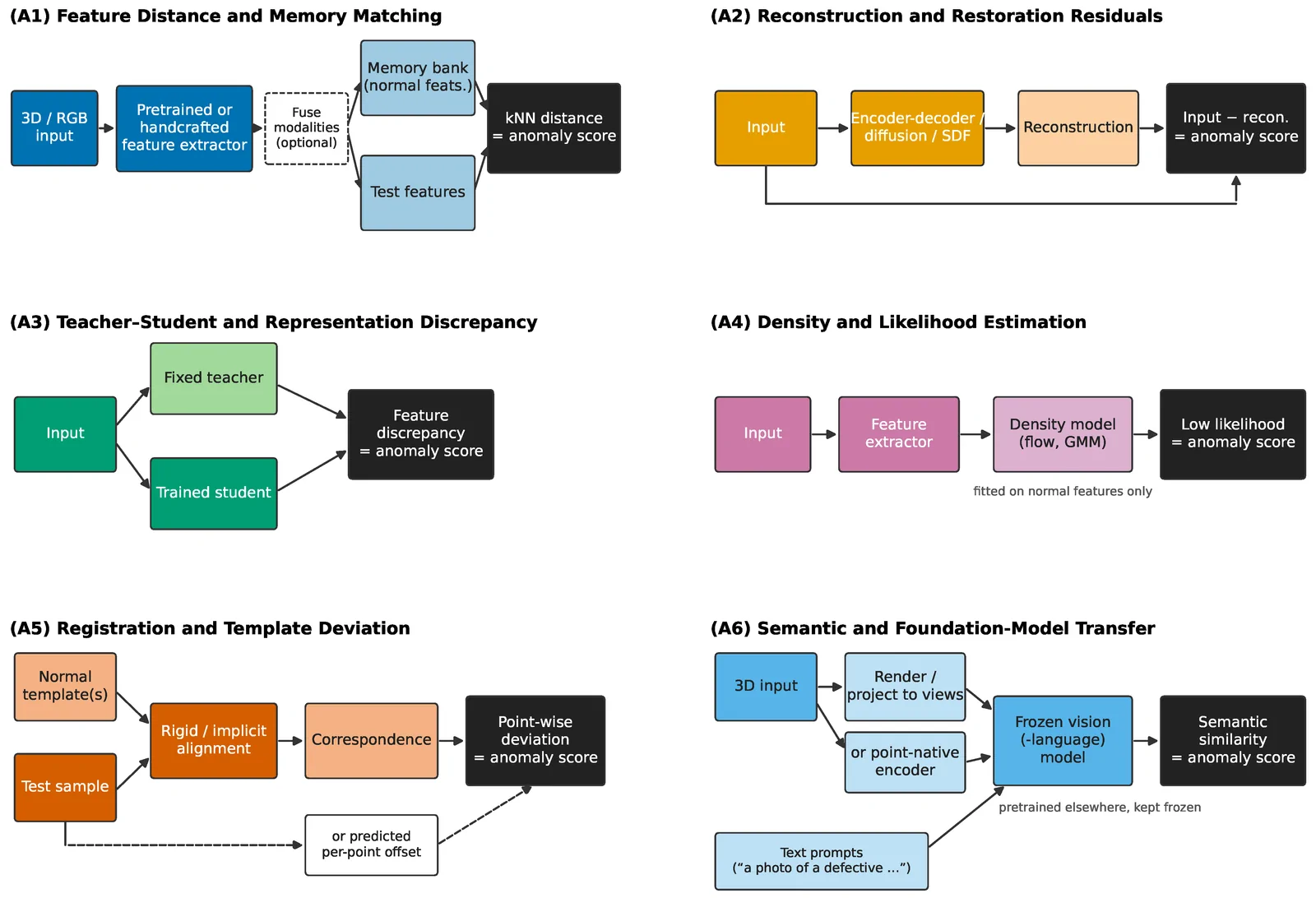
Processing pipelines of the six scoring mechanisms of Section 3.1, labeled A1 to A6 as such. The dark box in each panel is the quantity thresholded at test time. Modality fusion appears as the dashed optional branch in A1 because it is an input-path property that may precede any of the six, not a mechanism competing with them.

Each mechanism’s canonical scoring rule is formalized in its own section (Equations (2)–(4), (6) and (7)), and Figure 3 shows the pipeline behind each, ending in the quantity the assignment rule keys on.

Figure 2 draws the two axes and their positions; Figure 3 draws what each mechanism computes; and Table 5 places 31 of the 102 inventory works on both axes at once, chosen so that every Axis-A position and every Axis-B value a tabulated work occupies appear at least once; input-level fusion and open-set supervision have no representative because no work in the table uses them. The table carries six of the eight cross-cutting dimensions, including the representation a method learns or reuses; those six being where the representative works differ; five are recorded for every work in the released inventory.

## 4. Feature Distance and Memory Matching

Feature embedding and memory bank methods are the most established paradigms for 3D anomaly detection. The three-stage pipeline of Section 3.1, extract, store, and match, differs at each stage.

### 4.1. Adapting 2D Pretrained Features to 3D

No 3D pretrained extractor exists at the scale and diversity of the ImageNet-pretrained CNNs and vision transformers 2D methods reuse for free.

#### 4.1.1. Multi-View Projection

CPMF [17] projects 3D point clouds onto multiple 2D depth map views and extracts features from pretrained 2D networks (ResNet). The projected features carry global semantic information complementing local geometric cues, “complementary pseudo multimodal features”, without real multi-modal input. On MVTec 3D-AD, CPMF reaches 95.2% I-AUROC from point cloud input alone, competitive with methods that require paired RGB.

#### 4.1.2. Hybrid 2D + 3D Features

BTF (Back to the Feature) [23] concatenates features from a pretrained 2D CNN on RGB images with handcrafted FPFH descriptors [40] computed on point clouds. It achieved the strongest localization results at its 2023 publication, with P-AUROC of 99.3% and AUPRO of 96.4% on MVTec 3D-AD. Its image-level score (I-AUROC 86.5%) appears in Section 13.2.

### 4.2. 3D-Native Feature Extraction

#### Handcrafted vs. Learned Features

Hybrid pipelines containing FPFH remain competitive with learned 3D features, M3DM’s Point-MAE bank [22] among them, under particular benchmark and modality conditions. BTF and CPMF both pair FPFH with pretrained 2D features, so neither isolates a pure FPFH-only result, and the evidence for the descriptor itself comes from their ablations rather than their headline numbers. Three properties explain it: it targets the geometry surface inspection cares about, it is rotation-invariant and so needs no canonical pose alignment, and it carries no domain gap, where Point-MAE and Point-BERT are pretrained on furniture and vehicles. The advantage is conditional on the benchmark: FPFH-based methods (BTF, CPMF) are strongest on MVTec 3D-AD, where paired RGB carries much of the detection signal, while the leading peer-reviewed methods on the geometry-only, few-shot Real3D-AD are registration-based (Template3D-AD, 3DKeyAD, Section 13.3).

### 4.3. Memory Bank and Coreset Approaches

The memory-bank paradigm of PatchCore [2] (Section 2.3) meets three obstacles in 3D: feature dimensionality, point cloud density variation, and the need for spatial alignment.

#### 4.3.1. Anomaly Scoring

For a memory bank $M={\left\{m_{k}\right\}}_{k=1}^{K}$ of normal feature vectors, the anomaly score of a query feature $f_{j}$ at position *j* is

(2) $$a_{j}=\min_{m_{k}\in M}{∥f_{j}-m_{k}∥}_{2},$$

and the image-level score s(x) is typically $\max_{j}a_{j}$ or a percentile-based aggregation. Coreset subsampling [2] reduces M to a representative subset while maintaining coverage.

#### 4.3.2. Reg3D-AD

Introduced alongside the Real3D-AD dataset [19], Reg3D-AD adapts PatchCore to high-precision point clouds, aligning test clouds to normal prototypes via rigid registration (RANSAC) before extracting dual features (Point-MAE and raw geometric) for memory bank matching. Registration is critical for 360-degree point clouds where spatial alignment cannot be assumed. Reg3D-AD achieves an O-AUROC of 70.4% and a P-AUROC of 70.5% on Real3D-AD, the first reported results on that benchmark.

#### 4.3.3. PointCore

PointCore [71] improves upon Reg3D-AD with spatially anchored local-global features, matching a query against memory entries from the same spatial region rather than by global nearest-neighbor search. The paper reports a 17.75% O-AUROC gain over Reg3D-AD on Real3D-AD in its own comparison and an inference time of about 3.88 s per object. We do not re-derive the margin from Section 13.3, where the two entries were produced under separate protocols.

#### 4.3.4. Group3AD

Group3AD [72] structures the memory bank itself, grouping local patches by geometric similarity through group-level contrastive learning [73] so that retrieval runs over groups. Its authors report a 5% O-AUROC gain over Reg3D-AD on Real3D-AD in their own comparison.

#### 4.3.5. Shape-Guided Dual-Memory Learning

Shape-Guided [46] employs two complementary memory banks, one storing texture features from RGB images and another storing geometric features guided by explicit 3D shape priors, which the shape guidance aligns to a canonical representation. On MVTec 3D-AD it achieves 94.7% I-AUROC and 97.6% AUPRO.

#### 4.3.6. M3DM: Multi-3D-Memory

M3DM [22] constructs multiple memory banks, one per modality and feature scale, and aggregates their scores rather than searching a single bank. Its point-cloud bank holds patch-level Point-MAE [43] features, the pretrained masked autoencoder of Section 2.4, with Point-BERT [42] explored as an alternative, both pretrained on ShapeNet [74] and ScanObjectNN [75]; its image bank adds CLIP-based [76] visual features, which give semantic awareness for anomalies that combine unusual shape and appearance. How the banks are fused is described in Section 10. On MVTec 3D-AD, M3DM [22] reaches 94.5% I-AUROC.

#### 4.3.7. Rotation Invariance, Synthesis, and Preprocessing

Six further methods contribute a mechanism rather than a tabulated value. Reg2Inv [77] trains registration against normal prototypes to learn rotation-invariant features, so the score follows from the registration residual. RIF [78] instead computes descriptors that hold under arbitrary orientation. GLFM [79] matches global shape context against local geometric detail in a shared feature space, so one model covers categories without retraining and sharpens that boundary on synthesized anomalies. LCN [80] also trains on synthetic anomalies, as a translation task mapping an anomalous representation toward its normal counterpart so that the residual becomes the score. Simple3D [81] runs multi-scale neighborhood descriptors over 500K-point clouds at more than 20 FPS and arrives with the MiniShift dataset (Section 12). The Fence Theorem [82] is a preprocessing design principle rather than a detector, that semantic identity and structural normality should be kept apart since conflating them costs discriminative power.

#### 4.3.8. Spatial Context Aggregation

Hoang et al. [83] propose a single-forward-pass detector that grounds local point patches in learned global prototypes through optimal-transport alignment (Spatial Context Aggregation) and adapts frozen Point-MAE embeddings with a lightweight Feature Adaptor MLP. Its attention-based discriminator trains with a Selective Anomalous Feature Generator synthesizing hard-negative features rather than labeled defects. It runs at up to 13.5 FPS with consistent gains over prior memory-bank baselines on Real3D-AD and introduces the Industrial3D-AD dataset.

#### 4.3.9. LSFA: Self-Supervised Feature Adaptation

LSFA [84] addresses the domain gap of frozen ImageNet- or ShapeNet-pretrained extractors. It fine-tunes lightweight adaptors on the target data with a local-to-global self-supervised objective, jointly optimizing intra-modal adaptation and cross-modal alignment. Feature adaptation lifts memory-bank detection to 97.1% I-AUROC on MVTec 3D-AD (Section 13.2), among the stronger peer-reviewed results collated there, behind CMSA at 98.4%.

#### 4.3.10. AEMG and APG-Net: Prototypes Instead of Raw Banks

Two methods by Liu et al. attack the same weakness, that features stored directly in a memory bank are insensitive to intra-class variation, so the bank stores redundancy rather than coverage. AEMG [85] keeps a dual memory bank but adds attention-enhanced guidance so each modality retains a usable single-domain inference path, reporting 95.6% I-AUROC and 94.4% AUPRO on MVTec 3D-AD. APG-Net [86] replaces stored features with adaptive prototypes and targets the multi-sensor setting, reporting 97.4% object-level AUROC on MulSen-AD [87], a benchmark for which this survey collates no comparison table. Both illustrate a wider movement, from storing normal features to summarizing them.

#### 4.3.11. MISDD: Feature Matching When a Sensor Is Missing

Jiang et al. [47] ask what a multi-modal detector does when a sensor is unavailable at test time. Cross-modal prompt learning carries three prompts: for consistency between the two visual modalities, for adapting to each input pattern, and for compensating for the absent channel, while symmetric contrastive learning aligns each visual modality to text independently. Scoring stays in this family, a similarity to stored galleries of normal RGB, 3D, and text features. With the 3D channel missing at a rate of 30%, it reports 82.0% I-AUROC, 98.2% P-AUROC, and 92.7% AUPRO on MVTec 3D-AD; with both channels degraded at the same rate, 77.7% I-AUROC. The paper reports no full-modality baseline, so none of its figures are comparable to the leaderboards in Section 13, and the modality-missing configuration differs per baseline.

#### 4.3.12. MP3D and MambaAD3D: Multi-Scale Point Features

MP3D [88] targets vehicle stamped parts, large thin-walled components absent from the benchmarks, and reports 91.8% I-AUROC and 94.3% P-AUROC on its own stamped-parts dataset. Its MVTec 3D-AD figures of 95.0% I-AUROC and 98.5% P-AUROC average seven categories, with dowel, foam, and rope absent, so they are not comparable to the ten-category means quoted elsewhere. MambaAD3D [89] fuses tokens across scales through Mamba adapters and evaluates on point-clouds rather than RGB-D benchmarks, reporting 91.2% point-level AUROC and 86.8% object-level AUROC on Anomaly-ShapeNet and 76.6% point-level AUROC on Real3D-AD, alongside a self-collected RealScan-AD set.

### 4.4. Discussion

#### 4.4.1. Strengths

Feature embedding methods (Table 6) hold the peer-reviewed AUPRO ceiling we collate on MVTec 3D-AD, 98.2% by HFMM-Net, which the reconstruction method DAS3D [37] also reports, so the ceiling is shared across two mechanisms rather than owned by one. The pipeline is simple and training-light, and pretrained features are reused without domain-specific fine-tuning. A memory bank can be assembled from very few normal samples, which suits few-shot settings (Section 13).

#### 4.4.2. Limitations

Memory and compute grow with the feature bank, though coreset subsampling mitigates this. Sparse coverage of normal variation shows up as false positives. And because each product category needs its own memory bank, the one-class-one-model design scales poorly in flexible manufacturing.

## 5. Reconstruction and Restoration Residuals

Reconstruction-based methods build on the autoencoder [90]. A model trained exclusively on normal 3D data fails to reconstruct anomalous regions faithfully, so the elevated error is the anomaly signal. Given a reconstruction model ϕ trained on normal data, the score of the *j*-th point $p_{j}$ of a sample *x* is its distance to the reconstructed point set:

(3) $$a_{j}=\min_{\hat{p}\in \phi \left(x\right)}d\left(p_{j},\hat{p}\right),$$

where d(·,·) is a distance function (typically $\ell_{2}$ or learned). The set-to-set form, a single-sided Chamfer distance, is necessary because point-cloud reconstructions are permutation-ambiguous, so for unorganized clouds, the correspondence is by nearest point. Only for organized representations (depth maps, RGB-D grids) does the index-aligned form $a_{j}=d\left(p_{j},\phi {\left(x\right)}_{j}\right)$ apply.

Scoring under Equation (3) runs from input to reconstruction, so it detects material that is present and should not be but is blind to absent material, where no input point $p_{j}$ exists at which to evaluate the residual. Detecting it requires the reverse residual $\min_{p\in x}d\left({\hat{p}}_{j},p\right)$ over reconstructed points, a symmetric Chamfer distance, or a signed-distance formulation in which absent material registers as a sign change. Methods reporting only the one-sided score should be read as detecting additive rather than subtractive deviations.

### 5.1. Autoencoder and Masked Reconstruction Approaches

#### 5.1.1. IMRNet: Iterative Mask Reconstruction

Following masked autoencoders (Point-MAE [43]), IMRNet [49], proposed by Li et al. alongside the Anomaly-ShapeNet benchmark, masks portions of the input point cloud, reconstructs the missing regions, and at test time aggregates errors from several masking patterns into an anomaly map.

It uses neither pretrained models nor memory banks, learning normal geometry from scratch, so it needs enough normal training data to cover diverse geometric patterns. IMRNet achieves 66.1% O-AUROC on Anomaly-ShapeNet and 72.5% on Real3D-AD, early baselines that trail feature-based methods.

#### 5.1.2. EasyNet: Lightweight Reconstruction

EasyNet [91] reconstructs RGB and 3D input with no pretrained backbone and no memory bank, reaching 92.6% I-AUROC on MVTec 3D-AD; its throughput figure is read in Section 13.6. Section S10 of the Supplementary Materials describes the architecture.

#### 5.1.3. MC3D-AD: Multi-Category Unified Reconstruction

Most pipelines train one model per category. MC3D-AD [18] is, per its authors, the first unified multi-category model for point-cloud 3D anomaly detection (HGAD [33] did so earlier for RGB+3D input; Section 7). Its geometry-aware reconstruction network learns shared and category-specific priors through multi-category attention, and one model reaches 78.2% O-AUROC and 76.8% P-AUROC on Real3D-AD across all product categories, which its authors report as a 3.1% gain over the single-category baselines they train for the comparison.

#### 5.1.4. CM3D-AD: Consistency-Model-Based 3D Anomaly Detection

Pranav et al. [92] apply consistency models, which generate samples in two denoising steps rather than the hundreds standard diffusion requires, to 3D point cloud anomaly detection. The two-step inference reduces per-sample latency, addressing the slow iterative sampling of prior diffusion approaches such as R3D-AD [36] at the model-forward pass rather than across a deployed pipeline (Section 15.3).

### 5.2. Diffusion-Based Reconstruction

#### R3D-AD: Reconstruction via Diffusion

R3D-AD [36] applies diffusion models [93,94] to 3D anomaly detection, corrupting a point cloud through a forward process and rebuilding it through a learned reverse one that, trained on normal data, pulls anomalous regions toward their normal counterparts.

Each denoising step adjusts individual point positions, a step-wise point-level displacement. It also proposes Patch-Gen, a synthetic anomaly generation strategy that creates realistic point cloud defects by copying and deforming local patches. R3D-AD achieves 73.4% O-AUROC on Real3D-AD and 74.9% on Anomaly-ShapeNet, which the paper reports as an improvement over IMRNet in its own evaluation. Iterative inference reduces throughput relative to single-pass methods, though the authors report efficiency competitive with memory-bank baselines on Real3D-AD.

### 5.3. Implicit Neural Representations

#### 5.3.1. 3DSR/DADA: Depth-Aware Discrete Autoencoder

Zavrtanik et al. [48] proposed the Depth-Aware Discrete Autoencoder (DADA), which models RGB and depth in one discrete latent space, a vector-quantized variational autoencoder (VQ-VAE) [95] whose codebook is learned from paired data, so anomalies produce errors in both domains.

Because diverse industrial depth datasets are scarce, *depth simulation* synthesizes depth images during training to teach the depth encoder geometric features. The simulation runs only at training time, and at inference the model operates on the captured depth data. On MVTec 3D-AD, DADA achieves 97.8% I-AUROC and ranks first on 7 of 10 categories in the 3D evaluation setting.

#### 5.3.2. PASDF: Pose-Aware Signed Distance Fields

PASDF [50] represents 3D shapes as continuous signed distance fields (SDFs), which map each point to its signed distance to the nearest surface. Anomalies appear as deviations of the learned SDF from the expected normal surface.

A Pose-wise Alignment Module handles arbitrary orientations by registering the input into a frame the model learns rather than one supplied as a template. Because the SDF is continuous, one model both localizes anomalies and predicts the expected normal surface geometry, so detection and repair share one framework. PASDF reports 80.2% O-AUROC on Real3D-AD and 90.0% on Anomaly-ShapeNet, the highest peer-reviewed value in Section 13.4.

#### 5.3.3. SeDiR: Semantically Disentangled Reconstruction

SeDiR [96] separates semantic (category-level) from geometric (instance-level) information through a disentanglement module rather than learning one reconstruction function for all categories, and a geometry-guided decoder reconstructs point clouds from category-specific semantic priors and local geometric evidence. SeDiR reports 93.3% O-AUROC on Anomaly-ShapeNet in its unified multi-category setting (a 9.1% improvement over the second-best method) and a 2.8% improvement on Real3D-AD.

#### 5.3.4. SPA-SDF: Reconstruction Conditioned on Articulation

Gan et al. [97] attack the *rigid prior* shared by nearly every method here: that normal geometry is pose-invariant and canonicalizable to one template, so any residual deviation is a defect. For objects with hinge or sliding joints, joint motion is an intrinsic degree of freedom, and a method enforcing the prior reports valid articulation as an anomaly. SPA-SDF replaces the single template with a pose-conditioned implicit field factorized into an articulation-independent structural prior and a Fourier-encoded joint embedding, recovers the articulation state at test time by minimizing reconstruction energy, and scores the point-wise deviation from the resulting manifold. The paper reports 88.4% object-level AUROC on seen joint configurations and 87.4% on unseen ones, against 87.8% and 87.1% for the strongest baseline it runs, M3DM with Point-MAE features. Its benchmark, ArtiAD, is described in Section 12.

#### 5.3.5. V2G: Reconstructing Geometry from Appearance

Hoang et al. [98] train a network to predict geometric features from visual ones on normal data alone and score a sample by how far the predicted geometry falls from the observed geometry. The residual is cross-modal, which sidesteps the identical-shortcut failure discussed below. They report a mean I-AUROC of 96.8% on MVTec 3D-AD.

#### 5.3.6. DCMUNet: Reconstructing RGB and Depth in Separate Branches

Tang et al. [99] target coating defects, where the flaw is a thin surface layer, and reconstruct RGB and depth in two branches of a hybrid convolutional and Mamba U-Net before fusing their residuals. Depth defects are synthesized from Perlin noise modulated by point-cloud normal vectors, so the generated geometry follows the surface it sits on. The branches run independently, so a deployment with one modality uses the corresponding branch alone, which the authors measure rather than assert. On MVTec 3D-AD, the multi-modal model reaches 94.1% I-AUROC and 96.9% AUPRO, 3.7 points below 3DSR on detection and 1.4 below DBRN [100] on segmentation, and gains 3.2 points of I-AUROC over its own best single-modality variant. The method is built for coatings, and the authors state that its depth-only branch struggles on the large-area geometric defects of MVTec 3D-AD’s more complex shapes.

### 5.4. Discussion

Compared with memory banks, reconstruction methods trade the stored feature database for a generative model, and the anomaly map stays interpretable as a direct input-versus-reconstruction comparison. The paradigm also captures global structural anomalies that local feature matching can miss, and shared reconstruction networks extend to multi-category settings [18].

Three limitations bound the paradigm. The “identical shortcut” problem, where an overfit model reconstructs anomalous inputs as faithfully as normal ones, requires careful architectural design and regularization. Subtle local anomalies may be smoothed over, particularly by autoencoders that compress through a bottleneck. Diffusion-based methods mitigate this through iterative refinement but at the cost of inference speed. Alternative architectures address these issues: MAESTRO [101] is a multi-scale masked autoencoder on point clouds alone, without pretrained models or memory banks, and DUS-Net [102] adds group-center-preserving down-up sampling to keep anomalous geometry from leaking through the bottleneck. DRAIN [103] couples global structure reconstruction with a local inpainting branch and uses the discrepancy between the two to expose defect-specific residuals. C3D-AD [104] extends reconstruction to the continual setting of Section 15.3. A concurrent preprint, LDSD [105], learns discriminative signed distance functions from multi-scale features and is tabulated, as a preprint, in Section 13.3 and Section 13.4.

Reconstruction methods sit at the top of the MVTec 3D-AD results (Section 13.2) alongside memory-bank ones, 3DSR/DADA and FMFR within a point of CMSA at 98.4%, so reconstruction rivals memory matching once multi-modal learning is added. The progression from autoencoders to diffusion to implicit representations tracks advances in generative modeling, the most likely source of further gains, though whether they materialize remains uncertain.

## 6. Teacher–Student and Representation Discrepancy

Rooted in knowledge distillation [106], the teacher–student paradigm trains a student to replicate a pretrained teacher on normal data, and the discrepancy on anomalous input is the anomaly signal. For a teacher *T* and student *S* emitting features at *L* layers, the score of entity *j*, a pixel for organized input or a point for an unorganized cloud, is a weighted sum of per-layer discrepancies:

(4) $$a_{j}=\sum_{\ell =1}^{L}w_{\ell }\;d\;\left({\hat{T}}^{\left(\ell \right)}{\left(x\right)}_{j},\;{\hat{S}}^{\left(\ell \right)}{\left(x\right)}_{j}\right),$$

where ${\hat{T}}^{\left(\ell \right)}{\left(x\right)}_{j}$ and ${\hat{S}}^{\left(\ell \right)}{\left(x\right)}_{j}$ are the teacher and student features at layer *ℓ*, the hat denotes the per-layer normalization each method applies before comparison, *d* is a distance, and $w_{\ell }$ weights the layers. For image-like input, the layers are resampled to one grid so that *j* indexes the same location at every scale, as in STFPM [26]; for a point cloud, the correspondence is by point, and a method scoring at one descriptor layer has L=1 without resampling. 3D-ST [51] takes one layer of local geometric descriptors and scores each point by the $\ell_{2}$ distance between student and teacher, squared only in the training loss. AST [29] scores by that distance squared with L=1, but its teacher is a normalizing flow whose output is a density latent, which is why Table 5 records density estimation as its secondary mechanism.

### 6.1. Architecture Designs for 3D

#### 6.1.1. AST: Asymmetric Student–Teacher Networks

Standard teacher–student approaches carry a structural weakness: with both networks sharing one architecture, the student can learn an identity mapping that replicates the teacher even on anomalous inputs, collapsing the discrepancy. AST [29] targets this failure through *architectural asymmetry*: the teacher is a normalizing flow modeling the density of normal features, while the student is feed-forward. A student cannot trivially copy a density model, so it must learn a compressed normality representation that breaks down on anomalies.

AST processes RGB and 3D independently and fuses the anomaly maps, reaching 93.7% I-AUROC on MVTec 3D-AD [5]; the flow teacher descends from the same authors’ 2D CS-Flow [32].

#### 6.1.2. 3D-ST: Deep Geometric Descriptors

3D-ST [51] is, per its authors, the first unsupervised method to operate directly on 3D point clouds. Localization needs one forward pass through both networks, with no memory bank or iterative reconstruction. On MVTec 3D-AD it reaches 83.3% AUPRO, ahead of prior baselines at the time. Working on 3D data alone leaves it blind to texture defects visible only in RGB.

#### 6.1.3. Connections to 2D Foundations

Of the three 2D ancestors in Section 2.3, STFPM [26], Reverse Distillation [27] and EfficientAD [28], only Reverse Distillation has crossed into the 3D setting, extended to multi-modal industrial data by TRD [107], described in Section S10 of the Supplementary Materials.

#### 6.1.4. LPFSTNet: Parameter-Free Head Attention

LPFSTNet [108] attacks the paradigm’s compute cost rather than its accuracy. A lightweight student and a parameter-free head-attention module keep the discrepancy signal while cutting model size, with 94.7% I-AUROC and 99.2% P-AUROC reported on MVTec 3D-AD and no added inference time from the attention. It is one of few methods here treating latency as a design constraint (Section 15.3).

#### 6.1.5. DCRDF-Net: Dual-Channel Reverse Distillation

DCRDF-Net [109] applies reverse distillation across two channels, one per modality, and fuses them with an agreement criterion, so RGB–depth disagreement is itself evidence. It reports a 97.1% mean I-AUROC on MVTec 3D-AD. Its 98.8% localization figure is reported as “PRO” with no false-positive rate limit stated, which Section 13.1 keeps apart from the AUPRO values in Section 13.2 (Section 11.4).

### 6.2. Discussion

The teacher–student category is less prominent in 3D than in 2D anomaly detection, plausibly for two reasons.

#### 6.2.1. Lack of Pretrained 3D Teachers

In 2D, teachers are typically ImageNet-pretrained CNNs (ResNet [110], EfficientNet) or vision transformers [111]; the 3D counterparts (Point-MAE [43], Point-BERT [42]) are the weaker backbones of Section 15.2.

#### 6.2.2. MMRD: Reverse Distillation Across Modalities

Gu et al. [53] carry reverse distillation [27] into the multi-modal setting. A frozen siamese teacher encodes RGB and depth or surface-normal input separately and fuses the two encodings across levels, without learnable parameters, into the distillation target, which a learnable student decoder restores from priors learned on normal data. On MVTec 3D-AD with RGB and 3D input, they report 95.0% I-AUROC and 97.6% AUPRO, against 89.8% with RGB alone, so the geometric channel is worth better than five points. Reconstructing a *fused* target rather than mimicking a teacher layer by layer leaves the student less room to generalize onto anomalies. The paper’s second figure is pixel-level PRO rather than P-AUROC.

#### 6.2.3. Unstructured Nature of Point Clouds

The multi-scale pyramid matching of STFPM and EfficientAD [28] needs the image grid that Equation (4) resamples to; a point cloud has none, so the correspondence must be constructed, which 3D-ST does through local geometric descriptors.

#### 6.2.4. Advantages

Hierarchical feature comparison gives multi-scale detection for free, and the training dynamics are well understood from the 2D literature.

#### 6.2.5. Limitations and Future Potential

The primary limitation is the identity mapping AST’s asymmetry addresses, at the cost of normalizing flow training, and it is most pronounced where 3D features are less discriminative than 2D ones.

## 7. Density and Likelihood Estimation

Normalizing flow methods [112,113] model the probability density of normal features from a pretrained backbone by mapping them to a standard normal distribution, so that anomalies surface as points with low likelihood.

### 7.1. Normalizing Flow Fundamentals for Anomaly Detection

A normalizing flow [114] defines an invertible mapping $g_{\theta }:R^{D}\to R^{D}$ from a feature distribution $p_{F}$ to a base distribution $p_{Z}$ (typically standard Gaussian), so that by the change of variables formula the log-likelihood of a feature vector $f\in R^{D}$, extracted at one position of the input sample as in Equation (2), is:

(5) $$\log p_{F}\left(f\right)=\log p_{Z}\left(g_{\theta }\left(f\right)\right)+\log\left|\det\frac{\partial g_{\theta }\left(f\right)}{\partial f}\right|.$$

The model is trained to maximize this likelihood on normal features, so anomalous features receive low scores at test time, yielding the anomaly signal at position *j* with feature $f_{j}$:

(6) $$a_{j}=-\log p_{F}\left(f_{j}\right).$$

### 7.2. Flow-Based Approaches on 3D Features

#### 7.2.1. 2D Flow Methods Applied to 3D

The most common route to 3D has been indirect: extract features from 3D data using standard backbones, then apply one of the 2D flow architectures of Section 2.3 (CFlow-AD [30], FastFlow [31], or CS-Flow [32]) to the resulting distributions. Adapting them requires only swapping the 2D feature extractor for a 3D backbone (FPFH [40], Point-MAE [43]).

#### 7.2.2. HGAD: Hierarchical Gaussian Mixture Flow

HGAD [33] introduces a hierarchical Gaussian mixture normalizing flow for unified anomaly detection. Rather than fitting a single Gaussian base distribution, it models normal features as a hierarchically organized mixture of Gaussians, capturing their multi-modal nature across object categories and spatial positions, which supports multi-category detection within a single model.

#### 7.2.3. AST’s Flow-Based Teacher

AST [29], discussed in Section 6, uses a normalizing flow as the teacher network in a teacher–student framework.

#### 7.2.4. VAE-Based Density Estimation

VAE3D [34] employs a variational autoencoder to learn a likelihood model of normal point clouds, scoring anomalies by reconstruction and likelihood deviation. Evaluated on ShapeNet, it is among the first works to target general 3D objects rather than industrial surfaces.

#### 7.2.5. MVFM-3DAD: Flow Matching as a Density Proxy

MVFM-3DAD [54] reframes 3D anomaly detection as density-proxy estimation and is, as of our freeze date, the only member of this family reporting on the standard 3D benchmarks under a comparable protocol. A Bidirectional Geometric Projector converts a point cloud into multi-view representations, a Flow-guided Density Proxy Estimator assigns each view feature a reference density, and the backward projection maps the estimates back onto the points. Its motivation is stated against the other two mechanisms, which a density model avoids: memory matching is bounded by what the stored features cover, and reconstruction can learn the identity shortcut of Section 5. On Real3D-AD it reports 90.8% mean O-AUROC and 96.0% mean P-AUROC, and Section 13.3 records no higher value on either axis.

#### 7.2.6. MMFlows: Multi-Scale Normalizing Flow over Two Modalities

Qu and Lin [55] build a feature pyramid from a Vision Transformer over RGB and a Point Transformer over the point cloud, align the two by point-feature correspondence, and estimate density with a normalizing flow at several scales, keeping low-level features for small defects. On MVTec 3D-AD they report 93.3% I-AUROC, 98.9% P-AUROC, and 96.1% AUPRO (the abstract prints 98.8%; the tables 98.9%). MMFlows is one of the 5 members, and like HGAD it reaches 3D through a 2D backbone rather than by modeling point density directly.

### 7.3. Discussion

#### 7.3.1. Why Flows Are Less Dominant in 3D

Normalizing flow methods succeeded in 2D but have seen limited adoption in 3D. Three factors plausibly contribute:

1. **Feature quality dependence.** The flow models the distribution of normal features, so features that fail to capture anomaly-relevant information yield an uninformative density estimate, and pretrained 3D features are the weaker ones of Section 4 and Section 15.2.
2. **Feature space dimensionality.** 3D feature spaces differ in dimensionality from 2D CNN features, FPFH descriptors being 33-dimensional against 384 or 768 for Point-MAE, so architectures optimized for 2D features may not transfer optimally, and the grid-structure problem of Section 6 affects this family too, since the coupling layers assume a fixed input layout.
3. **Limited 3D-specific flow architectures.** Existing flow architectures for 3D point clouds were built for *generation*, and their adaptation to detection remains underexplored.

#### 7.3.2. Advantages

Unlike memory bank methods with their heuristic distance thresholds, flows define an explicit density model and, for flows proper, score by an exact likelihood, which places the anomaly score on a probability scale. This does not remove the thresholding problem: a likelihood is no easier to calibrate against a target false-alarm rate than any other score. Inference requires no memory bank to search, so prediction cost is independent of the normal set size, although it still scales with point count, feature dimension, and network depth.

Until MVFM-3DAD [54] neither advantage had produced a competitive 3D result, and Section 13 states what its one preprint value does and does not settle.

#### 7.3.3. Future Potential

As 3D foundation models improve and supply higher-quality features, flow-based detection in 3D should improve with them, and flows designed for point cloud feature spaces rather than borrowed from 2D could make density-based detection competitive in 3D, one such route being HGAD [33].

## 8. Registration and Template Deviation

Registration is the one mechanism in this taxonomy with no 2D anomaly detection ancestor (Table 3): it becomes a detection paradigm only once pose variation is intrinsic to the data, and the point cloud registration literature already supplies the alignment machinery.

The family shares an *align-then-deviate* template. Given a test sample *x* and a normal template *t*, a rigid transform is estimated and per-point deviation is scored:

(7) $$\begin{array}{cc} \rho^{*} & =\underset{\rho \in SE(3)}{\arg\;\min}\;d_{\mathrm{reg}}\left(\rho (x),\;t\right),\\ a_{j} & =\min_{p\in t}\;{∥\rho^{*}{\left(x\right)}_{j}-p∥}_{2}, \end{array}$$

where $d_{\mathrm{reg}}$ is the registration objective and $a_{j}$, point *j*’s deviation, may equivalently be measured in a feature space. The methods differ in what is registered, how many references are kept, and where deviation is measured. They give the three sub-families this section follows: explicit template registration, offset prediction that bypasses the alignment, and view synthesis that aligns poses through a differentiable renderer.

### 8.1. Explicit Template Registration

#### 8.1.1. Reg3D-AD

Reg3D-AD [19] scores by memory-bank matching and is described in Section 4; it appears here because its RANSAC [115] alignment step is the one this family later generalized into a scoring mechanism.

#### 8.1.2. Template3D-AD

Template3D-AD [56] matches on center points instead of whole clouds and extracts multi-scale features, including local curvature aggregation, from just *one* normal reference template per category. On Real3D-AD it reaches 84.4% O-AUROC, the leading peer-reviewed value in Section 13.3 (reported as a 1.5% improvement at publication), and 86.5% O-AUROC on Anomaly-ShapeNet (reported as an 11.6% improvement, first in 32 of 40 categories).

#### 8.1.3. 3DKeyAD

3DKeyAD [57] scores cluster-wise discrepancies between registered test clusters and keypoint-centered normal prototypes rather than point-wise deviations, which suits it to high-resolution clouds, and reports 80.1% O-AUROC and 86.1% P-AUROC on Real3D-AD. Supplementary Section S10 describes the clustering.

### 8.2. Offset Prediction

#### 8.2.1. PO3AD

Rather than aligning to a template, PO3AD [59] predicts point offsets: the displacements that would transform an anomalous point cloud into its normal counterpart. The offsets are themselves the anomaly signal, and the alignment step is absorbed into the offset regressor. A normal-vector-guided augmentation scheme supplies pseudo anomalies for training. PO3AD reaches 76.5% O-AUROC on Real3D-AD; its authors report average detection improvements of 1.4% on Real3D-AD and 9.0% on Anomaly-ShapeNet over prior methods.

#### 8.2.2. MC4AD: Corrective Forces as Offsets

Liang et al. [116] take a physics-inspired view of the idea PO3AD formalizes, modeling deviation from normality as a mechanical force. A network predicts an internal and an external corrective force per point, and the resultant force magnitude is the point-level score, with its maximum as the object score. A diverse anomaly generation module supplies training pairs. The method operates on point clouds alone: the mechanics it names as complementary are the internal and external force components rather than two sensing modalities, and its backbone ablation covers PointNet, PointNet++, PointTransformer and DGCNN. Its MVTec 3D-AD numbers are reported as object-level AUROC on the benchmark’s 3D half, so they are not comparable with the image-level values of Section 13.2 and are not placed there (Section 13).

### 8.3. View Synthesis and Pose Estimation

#### R^3^-PAD: Aligning Illumination Before Aligning Pose

Wang et al. [117] identify a confound that sits underneath the whole view-synthesis sub-family. A pose-agnostic pipeline renders a reference view and compares the query against it, so any appearance difference the renderer fails to reproduce is scored as a defect. Gaussian splatting does not model reflections and similar lighting effects, and test-time illumination need not match training illumination. Neither is an anomaly. R^3^-PAD inserts a Retinex decomposition before the comparison, separating reflectance from illumination, and adds a multi-scale fusion stage for localization. It reports a 97.9% mean I-AUROC on MAD-Sim, averaged over five runs. It evaluates on the pose-agnostic benchmarks rather than MVTec 3D-AD or Real3D-AD, so it does not appear in either results table. It is a preprint.

### 8.4. Discussion

Alignment-based methods attack the fixed-viewpoint assumption most benchmarks rest on, which matters as deployment moves to arbitrary poses (Section 15.3). Along the axes of Equation (7), explicit template registration is the most direct: Template3D-AD’s table-leading O-AUROC from a single template shows that one well-chosen template can replace a memory bank, reducing storage and making the reference auditable. Offset prediction removes the test-time alignment step, trading an explicit correspondence for a single forward pass, and view-synthesis methods relax the input from point clouds to posed images, which reaches settings without 3D sensors but ties detection quality to rendering fidelity.

Two failure modes bound the paradigm. First, registration is ill-posed for deformable or highly symmetric objects, and alignment error propagates into the anomaly score, a failure mode that purely feature-based methods avoid; keypoint- and cluster-based variants (3DKeyAD) mitigate but do not eliminate it. Second, it presumes a trustworthy template and inherits any variation in the reference: rotation-invariant features (RIF, Section 4) and pose-aware implicit fields (PASDF, Section 5) attack the same pose problem without an explicit alignment step, and which route prevails on deformable parts remains an open question (Section 15).

## 9. Semantic and Foundation-Model Transfer

The score here is a distance in a pretrained model’s semantic space (Section 3.1). Dedicated 3D foundation models remain scarce, so this family bridges to 2D models such as CLIP through projection or aligns point-native encoders to language.

A common alternative name, “foundation-model and zero-shot”, joins two things that are not the same. Transfer is how the score is computed; zero-shot describes what training data were available (Section 2.2). The two usually coincide here but are separable in both directions: a method can transfer foundation-model semantics while fitting a handful of normal references, and a method can be zero-shot without a foundation model, so supervision is recorded on the cross-cutting axis (Section 3.2).

PointAD: CLIP-Based Zero-Shot 3D AD.

PointAD [20] renders point clouds into multi-view 2D images, extracts features with a frozen CLIP [76] visual encoder, and projects them back to 3D for scoring. Learnable text prompts in the CLIP text space separate normal from anomalous regions without category-specific training data.

On MVTec 3D-AD, PointAD reaches 82.0% I-AUROC and 94.2% AP in the zero-shot setting. The gap against fully trained methods, which exceed 97%, is what forgoing category-specific training currently costs.

3DzAL: Zero-Shot 3D Anomaly Localization.

Where most zero-shot work stops at detection, 3DzAL [60] targets 3D anomaly *localization*. It trains a patch-level contrastive framework on pseudo anomalies from task-irrelevant 3D data, and a normalcy classifier, with adversarial perturbations on input patch coordinates, produces classification-based and feature-distance-based anomaly scores.

PMVP-PCLIP: Multi-View Projection for Zero-Shot Point Cloud Anomaly Detection.

MVP-PCLIP [61] builds its zero-shot detector on multi-view projection and CLIP features, matching per-view features against text prompts for normal and anomalous appearance. Aggregating view-level scores softens the information loss of any single projection angle. MVP-PCLIP and PointAD [20] differ mainly in this step: PointAD scores a fused representation, MVP-PCLIP votes over per-view scores.

BTP: Back to Point, Point-Language Models.

BTP [66] processes point clouds directly with a point-language model rather than through 2D projection, training a point-native transformer to align with language features, so normal object properties and anomaly types can be specified in text in 3D space, and there is no 3D-to-2D projection step at which geometry is lost.

Vision-Language Models for 3D.

A few general-purpose foundation models feed into 3D anomaly detection from the side. AnomalyGPT [39] and AnomalyCLIP [38] bring large vision–language and object-agnostic prompt learning from the 2D setting that 3D methods build on. SAM (Segment Anything Model) [118] supplies class-agnostic segmentation useful for localization, and DINOv2 [119] features have driven training-free, zero-shot 3D brain MRI anomaly detection through multi-axis 2D slicing and volumetric token aggregation [120].

ZUMA: Training-Free Zero-Shot Multimodal Detection.

ZUMA [121] targets the setting with no normal samples at all, which the authors motivate by privacy and confidentiality constraints. Cross-domain calibration builds a shared embedding space for RGB and point-cloud input, and dynamic semantic interaction grounds that space in natural language. It reports 84.0% I-AUROC, 97.6% P-AUROC, and 92.5% PRO on MVTec 3D-AD in the multi-modal setting. A fine-tuned variant, ZUMA-FT, adds a few learnable parameters. The paper names its metrics I-AUC and P-AUC, which we report under the survey’s I-AUROC and P-AUROC labels.

Where & What Anomaly?: Detection Plus Semantic Classification.

Huang et al. [122] treat binary detection as an incomplete formulation and produce a defect *label* alongside a localization map, zero-shot and pose-agnostic. On the LEGO subset of the pose-agnostic benchmark family, they report a 99.7% point-level AUROC and 97.1% AUPRO, with per-object semantic classification evaluated separately. Section 15.4 names the move from binary detection to defect characterization as one of three field-defining challenges, and this is the clearest attempt at it in our corpus. Its scope is narrower than the challenge: the semantic vocabulary is fixed in advance, and severity and physical extent are not estimated.

PointAD+: Hierarchical Representations for Zero-Shot 3D.

Zhou et al. [63] extend PointAD with explicit point-level representation learning alongside the rendered-view branch. In the point-only zero-shot setting on MVTec 3D-AD, they report 82.5% I-AUROC and 96.9% P-AUROC against PointAD’s 82.0% and 95.5%, and feeding RGB into the point branch raises the same benchmark to 87.7% I-AUROC, 98.2% P-AUROC, and 93.1% AUPRO. Transferred across datasets to Real3D-AD, PointAD+ falls below its own one-vs-rest scores on every metric, P-AUROC from 76.0% to 72.5%, I-AUROC from 83.5% to 74.3%, and AP from 84.7% to 78.2%, while PointAD decays barely at all. The authors attribute the loss to the geometry gap between source and target point clouds, which explicit point learning is exposed to and a rendered view is not.

MCL-AD: Collaboration Across Point Cloud, Image, and Text.

Li et al. [64] argue that zero-shot 3D methods lean on geometry alone and leave the RGB channel’s texture and color cues unused. Their framework learns an object-agnostic decoupled text prompt under a multi-modal contrastive loss, then modulates an RGB-guided and a point-cloud-guided branch against each other. On MVTec 3D-AD, it reports 89.0% I-AUROC and 96.9% AP at the image level, and 97.7% P-AUROC with 92.2% AUPRO at the pixel level, above PointAD on both levels. Removing the point-cloud prompt anchor degrades the fused result across metrics, which the authors read as evidence that the point branch contributes rather than rides on CLIP.

### Discussion

*Projection-based* approaches (PointAD, MVP-PCLIP, and GS-CLIP) render point clouds to 2D and exploit mature CLIP features, at the cost of irreversible geometric information loss. *Point-native* approaches (BTP) align a 3D encoder to language directly, but cannot yet draw on a 2D model’s web-scale pretraining.

## 10. Modality and Fusion Across the Mechanisms

Fusion is the first of the cross-cutting dimensions of Section 3.2, and it has its own section because it carries more of the reported performance on RGB-paired benchmarks than any single scoring mechanism does. A fusion method still scores by one of the six mechanisms of Section 3.1, and Table 7 records which, from what each paper says, produces its anomaly score; methods with a home section elsewhere have only their fusion machinery treated here.

Color images capture surface texture and color variation, while 3D data carries shape, curvature, and surface topology, so anomalies invisible in one modality are often obvious in the other. Methods using both occupy most of the upper range of reported detection scores on benchmarks that provide both [5], a fact about those benchmarks as much as about the methods, to which Section 13 returns.

One placement is a closer fit rather than an exact one: BridgeNet [67] is recorded under (A2) with the fit argued in Section 3, the only such case among the 94 detectors and 8 related techniques; DAS3D and DBRN [100] reconstruct, so the residual reading is literal for them.

### 10.1. RGB+3D Fusion Strategies

#### 10.1.1. M3DM: Fusion Measured Within One Implementation

M3DM [22] is described in Section 4, and its fusion levels are placed in the taxonomy below. What this section adds is the ablation: the paper’s own 3D-only variant reaches 87.4% against the multi-modal value in Section 13.2, so the gain from fusion is measured within one implementation rather than across papers, the only setting in which such a margin is controlled.

#### 10.1.2. M3DM-NR: Noise-Resistant Extension

M3DM-NR [123] keeps M3DM’s backbone and adds CLIP-based [76] denoising that down-weights the regions industrial depth sensors corrupt on reflective and transparent surfaces (Section 11.3, Section S10 of the Supplementary Materials). Its noise is training-set contamination rather than sensor noise, so Table S12 does not count it among the sensing experiments.

#### 10.1.3. Shape-Guided Dual-Memory Learning

Shape-Guided [46] is described in Section 4; what concerns fusion is that its geometric bank is pose-normalized before the modalities meet, so texture and geometry are compared in a common frame rather than concatenated in whichever pose the scan arrived in. Its AUPRO is matched or exceeded in Section 13.2 by CMSA [124], DGCF [125], MMRD [53], FMFR [126], V2G [98], and HFMM-Net and DAS3D [37] at the 98.2% ceiling, all within a point of each other, and by one entry reported as PRO with no stated limit, which Section 13.1 keeps apart (Section 11.4).

#### 10.1.4. CPMF: Pseudo Multi-Modal Fusion

CPMF [17] (Section 4) needs no color sensor, which suits point-cloud-only benchmarks such as Real3D-AD [19]; what its rendered views buy on MVTec 3D-AD is read in Section 13.

#### 10.1.5. DAS3D: Dual-Modality Anomaly Synthesis

DAS3D [37] trains on synthetic dual-modality anomalies rather than fusing real signals, and Perlin noise-like deformations on depth maps with an augmentation dropout against over-reliance on either modality. Its reconstruction network reaches an I-AUROC on MVTec 3D-AD second only to CMSA (Section 13.2).

#### 10.1.6. MulSen-AD: Multi-Sensor Anomaly Detection

Li et al. [87] push fusion beyond RGB+3D to three sensors capturing appearance (RGB), surface geometry (point cloud), and internal structure, aligned across modalities and scored jointly. Section 12 describes the MulSen-AD dataset.

#### 10.1.7. BridgeNet

BridgeNet [67] bridges 2D and 3D industrial anomaly detection with parameter-shared RGB and depth modules and dedicated generators, multi-scale Gaussian and unified texture synthesis, producing training-time defects in both modalities.

#### 10.1.8. MAFR: Fusion-Restoration

MAFR [127] pairs a shared fusion encoder with attention-guided decoders that each restore their own modality and aggregates the reconstruction errors for scoring, reporting 97.2% mean I-AUROC on MVTec 3D-AD, the highest we collate from a preprint on that benchmark, and 90.1% on Eyecandies.

### 10.2. Fusion Taxonomy

Fusion happens at one of three points. *Early* fusion concatenates or interleaves raw inputs before feature extraction, which demands an architecture that can ingest heterogeneous inputs. *Mid-level* fusion combines features extracted independently from each modality and is the most common strategy, the standard example being M3DM’s Point Feature Alignment and Unsupervised Feature Fusion; BTF’s [23] joint memory bank (Section 4) is the plain-concatenation case, and its localization result suggests that fusion need not be elaborate when the feature types are already complementary. *Late* or decision fusion runs each modality through its own pipeline and combines the scores, as M3DM’s Decision Layer Fusion does. M3DM and Shape-Guided each span the middle and late levels; neither has a documented raw-input early-fusion operation.

#### 10.2.1. Recent Fusion Designs

2M3DF [65] fuses multiple perspectives as well as modalities, combining global semantic context, local geometry, and color, reporting 96.6% mean I-AUROC. UCF [128] questions the assumption that RGB and point features carry an explicit spatial correspondence and combines uni-modal and cross-modal paths instead, reporting 96.3% I-AUROC. DGCF [125] makes the disagreement between modalities the signal rather than averaging it away, reporting 97.3% I-AUROC and 97.6% AUPRO. UniMF [129] addresses a single model across categories and a handful of normal samples together, reporting 76.9% I-AUROC under a one-shot protocol not comparable with the full-data values in Section 13.2 and therefore not placed there.

These mark a shift from how to combine two feature streams to which stream to trust where, with the answer spatially and categorically variable.

#### 10.2.2. LM3M: Compressing the Memory Bank, and a Clean Reproduction

Zhang [130] applies transformer compression to M3DM’s RGB branch, reporting 92.9% I-AUROC, 98.5% P-AUROC, and 95.6% AUPRO on MVTec 3D-AD against 94.5%, 99.2%, and 96.4% uncompressed, and cutting mean inference from 13.05 s to 9.33 s, a 28.6% reduction at a mean cost of about one point, which speaks to the latency constraint Section 15.4 names. Its uncompressed baseline reproduces M3DM’s published means exactly, 96.4% AUPRO and 94.5% I-AUROC, which is rare in this literature. The 13 s is a desktop research runtime, and the author declines to claim embedded deployment, so it is a relative saving, not an inspection-cycle figure.

#### 10.2.3. CDRS: Scoring by Cross-Modal Disagreement, and a Baseline Gap

Xu et al. [131] treat disagreement between the RGB and 3D branches as a reliability signal, down-weighting the branch it implicates, and report 80.0% I-AUROC, 98.0% P-AUROC, and 92.6% AUPRO on MVTec 3D-AD. The image-level figure falls far below the rest of this section because the comparison is against the authors’ own run of the released M3DM code under its official full-setting command, which scores 77.9%, not the 94.5% M3DM publishes. The paper does not resolve whether that gap is a configuration difference, and neither can we from the outside. We report the pair, because an improvement over a reproduction sixteen points below its published value is a different claim from an improvement over the published state of the art.

#### 10.2.4. CMSA, HFMM-Net, and FMFR: Three Takes on the Fusion Stage

Three works keep the memory bank scoring of this family and rework how the modalities meet. CMSA [124] aligns RGB and point features in a shared semantic space with an adapter and a contrastive term, reaching 98.4% I-AUROC and 97.6% AUPRO@30% on MVTec 3D-AD and 90.3% I-AUROC on Eyecandies. Its AUPRO at a 1% false-positive limit, 46.3% against CFM’s 45.5%, shows how much of the apparent agreement lives in the permissive 30% limit the field quotes by default. HFMM-Net [132] swaps attention-based fusion for a Mamba state-space block for cost, reporting 95.6% I-AUROC, 99.3% P-AUROC, and 98.2% AUPRO@30%, and FMFR [126] remaps in several stages instead of one, reporting 98.0% mean I-AUROC and 97.9% AUPRO@30%. The three land within about three points of each other at the image level while differing in fusion mechanism, adapter design, and cost, as the pattern Section 13 describes here.

### 10.3. Discussion

#### 10.3.1. Recent Advances

CFM [133] learns an explicit map between modality feature spaces; Long et al. [134] benchmark fusion architectures against each other; ISMP [135] mines extra cues inside the 3D modality; LSFA [84] adapts each modality’s pretrained features before fusion. The levers are largely orthogonal: the fusion mechanism, the input signal, and the per-modality features.

#### 10.3.2. Dominance on MVTec 3D-AD

Entries with real multi-modal input occupy most of the upper range of the MVTec 3D-AD results (Section 13.2), CPMF excepted (Section 4), and MP3D [88] is not tabulated there because its value is a mean over seven of the ten categories rather than the full benchmark.

#### 10.3.3. Applicability Constraints

The requirement for paired multi-modal data, captured simultaneously and with spatial alignment, limits these methods. Real3D-AD provides only point clouds, and many industrial scenarios use single-modality sensors, which CPMF’s pseudo multi-modal approach only partially addresses.

The fusion dimension is where the difference between what MVTec 3D-AD and Real3D-AD reward is concentrated (Section 13).

## 11. Sensing Conditions as Determinants of Detectability

Every method in this survey consumes data produced by a physical instrument, and most through a benchmark that fixed the instrument years earlier. A defect that the sensor did not resolve cannot be recovered by any scoring mechanism.

### 11.1. Sensor Principles and What They Cost

Table 8 lists the acquisition principles behind the benchmarks of Section 12. The limits are not asserted from first principles: structured-light pattern design trades acquisition speed against correspondence ambiguity [136]; laser triangulation’s uncertainty grows with standoff and with the angle between surface normal and baseline, with speckle setting a floor on spot localization [137,138]; time-of-flight carries multi-path interference, wiggling error, and a depth bias varying with integration time and reflectivity [139]; and photogrammetric accuracy depends on network geometry, image scale and surface texture [140]; lock-in infrared thermography is the MulSen-AD [87] case in the table.

The principle a dataset used is not always recoverable from its paper. Real3D-AD calls its capture “high-resolution laser scans” while describing the instrument, two pages later, as a binocular system with a high-luminance LED and a blue-ray filter, which is structured light rather than laser triangulation [19]. We record it as structured light on the strength of that description.

Eyecandies is rendered in Blender [141] and Anomaly-ShapeNet derives from ShapeNet CAD models [49,74], so their depth is noiseless and complete in a way no instrument achieves, which matters when a method tuned on them is called ready for deployment.

**Table 8:** Acquisition principles behind the benchmarks of Section 12, with the surfaces on which each characteristically fails. “Coverage” distinguishes a single viewpoint, which yields an organized 2.5D image, from multi-view capture stitched into full geometry.

Principle	Used by	Coverage	Characteristic Failure	Consequence for Detection
Structured light	MVTec 3D-AD [5]; Real3D-AD [19]	Single view (MVTec); multi-view stitched (Real3D-AD)	Specular, transparent, and dark surfaces return no pattern	Missing points are indistinguishable from missing material to a one-sided residual
Laser triangulation	Not evidenced in these benchmarks; named by Real3D-AD [19], which we record as structured light (Section 11.1)	Line sweep, usually multi-view	Slower; sensitive to part motion during the sweep	Acquisition time enters the cycle-time budget, not just inference
Photogrammetry/multi-view stereo	PAD (multi-pose imagery) [142]	Multi-view	Requires surface texture to match across views	Weak on the untextured machined surfaces inspection targets present
Lock-in infrared thermography	MulSen-AD [87]	Subsurface (our reading)	Lower lateral resolution than surface methods (our reading)	Reaches the shallow-layer anomalies and internal cracks its source names
Rendering (no sensor)	Eyecandies [141]; Anomaly-ShapeNet [49]	Arbitrary	Depth is complete and noise-free by construction	No missing-data or calibration failure modes to learn from

### 11.2. Six Quantities That Are Not Interchangeable

Comparisons routinely rank benchmarks by a single number under a heading such as “precision” or “resolution”, collapsing six distinct quantities. Four of them, trueness, precision, repeatability and measurement uncertainty, are used in the sense of the international vocabulary of metrology [143] and, for the last, of the guide to the expression of uncertainty in measurement [144]. Axial resolution follows the vocabulary’s *resolution*. Lateral sampling distance is not a metrological term but a property of the sampling grid and the quantity most often reported in place of the other five:

- **Lateral sampling distance**, also called point spacing: the distance between neighboring samples on the surface, which bounds the smallest feature that can be represented.
- **Axial resolution**, or depth resolution: the smallest change along the viewing direction that produces a change in the reported value, a quantization limit rather than an error bound.
- **Trueness**: closeness of agreement between the mean of many measurements and a reference value, that is, the absence of systematic bias.
- **Precision**: closeness of agreement among repeated measurements, that is, the absence of scatter. We avoid *accuracy* for the pair, because this literature uses it both for the combination and as a loose synonym for either.
- **Repeatability**: precision under the narrowest conditions, namely the same instrument, operator and setup within a short interval. Reproducibility, the corresponding quantity across changed conditions, is what a multi-station inspection line depends on and is never reported.
- **Measurement uncertainty**: a parameter characterizing the dispersion of the values attributable to the measurand [144]. Reported as an interval with a coverage factor and a level of confidence, it is the *expanded* uncertainty, the form an accept-or-reject decision needs. We use the term only where a source supplies such a statement, which in this corpus is nowhere.

A vendor’s nominal figure is not a smallest detectable defect.

Datasheet figures for lateral sampling or axial resolution are obtained on cooperative reference artifacts under controlled conditions and state what the instrument samples, not what it can distinguish on the part. The smallest defect a pipeline can detect is bounded below by the lateral sampling distance, by repeatability, and by the resampling step of Section 11.3, which discards resolution the sensor did deliver. A nominal number therefore establishes a necessary condition and never a sufficient one, and no benchmark in Section 12 reports the chain from that number to a detectable defect size.

Sub-millimeter capability does not establish metrological suitability.

An accept-or-reject decision against a dimensional tolerance is a measurement claim, and it requires traceability to a calibrated standard and a stated uncertainty with a coverage factor. A sub-millimeter sampling figure supplies neither, and a pipeline whose output is an unnormalized anomaly score has no path to a dimensional statement (Section 11.4). We therefore describe the systems in this survey as detecting geometric deviation rather than performing tolerance inspection.

Real3D-AD reports its acquisition characteristic in two places with two terms and two values an order of magnitude apart: the abstract gives “point cloud resolution (0.0010 mm–0.0015 mm)” while the introduction states that “the point precision of Real3D-AD reaches up to 0.010 mm” [19]. Neither says which of the six quantities is meant. The 0.11 mm commonly attributed to MVTec 3D-AD does not appear in the MVTec paper; it appears in Real3D-AD’s comparison against it [19], and the MVTec paper characterizes its data by sensor model and image resolution, 1920×1200 pixels [5].

We accordingly report an *acquisition characteristic* in Table 9 rather than a precision, with the term, its source, used and where, and leave the cell empty where a benchmark states none.

### 11.3. Calibration, Missing Data, and Density

#### 11.3.1. Calibration and RGB-Depth Alignment

Multi-modal methods (Section 10) assume a pixel and a point describe the same piece of surface. That correspondence is an extrinsic calibration, and its residual error is a geometric displacement no fusion architecture can distinguish from a real one. MVTec 3D-AD supplies organized point clouds in which the correspondence is exact by construction [5], and this is a rarely acknowledged part of why RGB-3D fusion performs so well there.

#### 11.3.2. Missing Data

The MVTec authors state that their data contains pixels “where the sensor did not return any 3D information due to, e.g., occlusions, reflections, or sensor inaccuracies” [5]. Every benchmark built on a physical instrument contains such gaps. A hole in the point cloud is what a missing-material defect looks like to the one-sided residual of Equation (3) (Section 5). Whether a method treats a gap as a defect, as an absence of evidence, or not at all is rarely reported.

#### 11.3.3. Point-Density Variation and Resampling

Density varies within a scan with surface orientation and distance and across scans with the setup. Nearly every pipeline resamples, by voxel downsampling or farthest-point sampling, before feature extraction, which changes the local neighborhood statistics over which the descriptors of Section 4 are computed, so a reported score depends on a preprocessing choice that comparison tables do not record.

#### 11.3.4. Sensor Drift and Cross-Sensor Transfer

Whether a model fitted on one instrument transfers to another is largely untested, because almost every benchmark uses one sensor throughout, with 3D-ADAM [145] the exception (Section 12). Longer-term drift between recalibrations is a routine concern in production metrology.

#### 11.3.5. Acquisition Time and Synchronization

Runtime discussions report model inference (Section 13), but a cycle-time budget covers the whole pipeline, whose acquisition frequently dominates: the multi-view stitched capture Real3D-AD uses requires part manipulation between views. Where several sensors contribute, as in MulSen-AD [87], a synchronization error on a moving part appears as a geometric inconsistency between modalities.

### 11.4. Ground-Truth Space and Metrological Limits

Annotations are defined in whichever space the acquisition produced, and that choice propagates into the metric. MVTec 3D-AD annotates on the image grid [5], Real3D-AD annotates points directly [19], and SiM3D evaluates in voxel space with V-AUPRO [146], so the entities being scored differ in count by orders of magnitude (Section 13.1).

**Figure 4:**
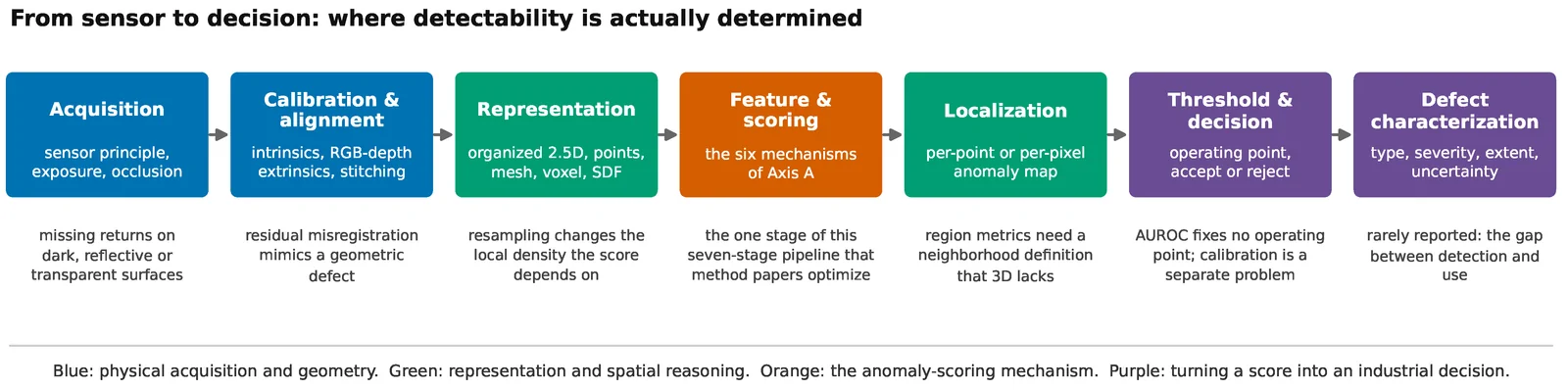
From sensor to decision. The scoring mechanism that a method paper contributes occupies one stage; the failure modes listed beneath each of the others are contributed by the sensing and deployment context and are largely invisible in benchmark scores. Colors group the stages by kind and are not the only carrier of that grouping, since each stage is also named.

Two limits follow from the sensing side, and neither is reported. The first is the *smallest detectable defect* of Section 11.2. No benchmark in Section 12 publishes a defect-size distribution against the sensor’s resolution, so what fraction of a benchmark’s defects sit near its instrument’s floor is unknown. The second is *metrological traceability*, an unbroken chain of calibrations to a standard, each with a stated uncertainty [143], which this literature’s unnormalized scores do not have. Figure 4 marks that gap as a stage of its own, and no work in this survey reports a coverage interval.

None of this diminishes the algorithmic contributions surveyed here. It bounds them: a benchmark score describes a method’s behavior under one instrument’s failure modes as much as its ability to detect anomalies in general.

### 11.5. What Has Actually Been Measured

Table S12 of the Supplementary Materials lists every experiment in the corpus that varies a sensing condition and reports the effect, 117 of them from 63 of the 108 works examined (the 98 inventory works with a source PDF and the 10 dataset papers, as in Section 13.6).

Point density and downsampling account for 26 of the experiments, a change of capture setup 24, additive noise 14, missing data 15, lighting 8, rotation 9, resolution 14, and calibration 7. What gets studied is what a method’s own design already exposes: a downsampling rate and a view count are hyperparameters an ablation sweeps anyway. What decides whether a deployed instrument’s output resembles the benchmark’s is almost never touched.

These are ablations in service of a design choice, not characterizations of an operating envelope. They establish that a method degrades under a condition, never at a point at which detection becomes unusable. Effects are quoted in each source’s own metric and units and not compared across rows.

The three conditions a deployed instrument is most likely to violate are the thinnest in that table. Calibration: 7 rows from 6 works, of which two vary anything. ActiveInspect [147] perturbs the extrinsic rotation and the focal length at test time and reports I-AUROC falling from 90.6% to 89.2% at a two-degree rotation error, and the Fence Theorem [82] reports detection scores tracking registration accuracy across three methods, which is sample-to-template alignment rather than sensor-to-sensor; the rest describe a rig’s calibration procedure without testing sensitivity to it. Cross-sensor transfer and drift (Section 11.3): the first almost unused, the second in no benchmark and no experiment. The methods surveyed here are therefore characterized against algorithmic difficulty, not against the acquisition conditions, with the exceptions Table S12 lists.

#### 11.5.1. Acquisition Designed Around the Defect: Highly Reflective Surfaces

Zeng et al. [148] change the capture: fringe saturation on specular surfaces leaves holes in the reconstruction (Section 11.3), so they adapt the projected pattern to local reflectance and segment defects with PointNet++. Their fringe-projection rig calibrates to average reprojection errors of 0.04 pixels for the camera and 0.05 for the projector, and they report 81.18% mIoU with 90.76% overall accuracy on Real3D-AD, plus 82.67% mIoU on a self-built reflective-surface set. It is listed among the related techniques rather than counted with the detectors: the segmentation is supervised on labeled defects, and mIoU is not comparable to the AUROC family. The premise is that acquisition quality on difficult materials is a property of the projector, not the detector.

#### 11.5.2. Choosing What to Acquire: Active Inspection

ActiveInspect [147] treats its observations as a budget: a policy decides at each step whether to request another observation or stop and return a verdict, with depth and point-cloud measurements converted into renderings a vision–language model can read. On Real-IAD D^3^ the paper reports 99.8% of the accuracy exhaustive evaluation of all fifteen observations attains while using 18% of them, with its largest gains on the geometry-dependent defect classes. It too is listed among the related techniques because the verdict comes from the underlying model rather than from a scoring mechanism it contributes. It measures an accuracy-per-observation trade-off, the quantity an inline inspection cell is constrained by, which no benchmark in Section 12 reports.

## 12. Datasets and Benchmarks

Progress in 3D anomaly detection has been paced by increasingly challenging benchmarks.

### 12.1. MVTec 3D-AD

The MVTec 3D-AD dataset [5] was, to our knowledge and among the datasets identified by our search, the first large-scale benchmark for unsupervised 3D anomaly detection in industrial settings; its acquisition figure, and the 0.11 mm attributed to it by Real3D-AD [19], are treated in Section 11.2. The split is 2656 training and 294 validation samples, both normal only, against 1197 test samples (249 normal, 948 anomalous) [5], with pixel-precise localization ground truth for scratches, dents, holes, contamination, and geometric deformations.

Three limitations bear on how its results read: (i) all objects are captured from a single top-down viewpoint, limiting assessment of view-invariant methods; (ii) reported values are compressed near the top of the scale, the five highest I-AUROC values within 1.1 points and AUPRO at 98.2% (Section 13, Table 10); and (iii) the small number of categories limits generalizability analysis.

### 12.2. Real3D-AD

Real3D-AD [19], introduced at NeurIPS 2023, answers MVTec 3D-AD’s single viewpoint with high-precision, full 360-degree point clouds and no RGB images. A binocular blue-light scanner (PMAX-S130) on a turntable stitches front and back views into clouds of 40,000 to millions of points per item [19], and Section 11.2 quotes its two conflicting acquisition figures. Anomaly types are bulge (material redundancy) and sink (incompleteness), with synthetic defects added through copy and edge-cutting operations.

The setting is inherently few-shot, four normal training samples per category against 50 normal and 50 anomalous test samples, which reflects what defect-free reference scans cost in industry. The dataset also introduced the Reg3D-AD baseline and the ADBENCH-3D benchmark.

### 12.3. Anomaly-ShapeNet

Anomaly-ShapeNet [49] expands the scope to 40 object categories derived from ShapeNet [74], with 8000 to 30,000 points per cloud and four normal training samples per category. A refinement module first enhances the ShapeNet meshes, then an injection procedure creates six types of synthetic defects with precise 3D ground-truth annotations. The number of categories enables evaluation of scalability across diverse object geometries on synthetic rather than measured surfaces.

### 12.4. Eyecandies

The Eyecandies dataset [141], introduced at ACCV 2022, is fully synthetic. Its candy samples are rendered photo-realistically by a simulated conveyor-belt setup under multiple lighting conditions for 90,000 image views.

Anomalies are procedurally injected into the rendering pipeline, so pixel-wise ground-truth annotations come automatically and data generation is unlimited, at the cost of a domain gap when a method trained here is deployed on real-world data. It evaluates multi-modal methods that combine RGB with depth and normal maps.

### 12.5. PAD: Pose-Agnostic Anomaly Detection

The PAD dataset [142] challenges the pose-alignment assumption underlying prior benchmarks. It features 20 complex LEGO toy categories with, in its own words, over 11,000 high-resolution multi-pose views, split into MAD-Sim (simulated) and MAD-Real (real-world) subsets. Table 1 of the PAD paper records 5231 normal and 4902 anomalous samples, which is 10,133 rather than 11,000, and the paper states the train and test proportions as ratios, roughly 2:1 for the simulated subset and 4:1 for the real one, rather than as counts.

Test images deliberately lack pose information, so a method cannot rely on spatial alignment with reference views. Its OmniposeAD baseline synthesizes reference views from arbitrary poses with Neural Radiance Fields (NeRF) [149]. PAD supplies no measured 3D data: its samples are RGB views with camera poses, and the geometry a detector uses on it is reconstructed from those views by a neural radiance field in the dataset’s own baseline and by Gaussian splatting or radiance fields in the corpus methods that report on it (SplatPose+ [150], R^3^-PAD [117], and Where&What [122]). It is retained as the benchmark on which the pose-agnostic detectors of Section 8 are evaluated, not as a 3D-sensor dataset, and Table 9 records it as such.

**Table 9:** Comparison of major 3D anomaly detection datasets. The **Reported acquisition characteristic** column quotes each source in its own words and names where in the source the wording appears (Section 11.2). *None* means we read the source and it states no such figure. The **Modalities** column lists the sensors or channels each dataset ships, not the input code of Section 2.1: PC = Point Cloud, D = Depth map, N = Normal map, PS = Photometric Stereo, IR = lock-in infrared thermography. Cat. = Categories.

Dataset	Year	Cat.	Samples	Modalities	Source	Reported Acquisition Characteristic	Key Feature
MVTec 3D-AD [5]	2022	10	4147	RGB + PC	Real	Zivid One+ Medium, 1920×1200 px, stated as “resolution” (§3.1, Data Acquisition). The 0.11 mm “point precision” widely attributed to it is stated by [19], not by the MVTec paper	Standard benchmark
Eyecandies [141]	2022	10	15,000	RGB + D + N	Synth.	None; depth and normals are rendered, not measured (abstract)	Multi-lighting
Real3D-AD [19]	2023	12	1254	PC	Real	“point cloud resolution (0.0010 mm–0.0015 mm)” (abstract) and “point precision … up to 0.010 mm” (introduction)	360°, few-shot
PAD [142]	2023	20	11,000+	RGB multi-view + poses; no measured 3D	Real+Sim	None; “4K views with various poses” (abstract) is a view count, not an acquisition figure	Pose-agnostic
PD-REAL [151]	2023	15	3500+	RGB + D	Real	RealSense consumer depth camera; no numeric figure stated (abstract)	Handmade Play-Doh objects
Anomaly-ShapeNet [49]	2024	40	1600	PC + Mesh	Synth.	None; derived from ShapeNet CAD models (abstract) [74]	40 categories
Real-IAD D3 [152]	2025	20	8450	RGB + PC + PS	Real	“a resolution as fine as 0.002 mm”, alongside “micrometer-level” point clouds (abstract)	Fine defects
SiM3D [146]	2025	8	333	RGB + PC + CAD	Real	“high-resolution images (12 Mpx) and point clouds (∼7M points)” (abstract)	Voxel-level GT
3D-ADAM [145]	2025	28	14,120	RGB + PC	Real	“captured with four industrial depth sensors” (abstract); per-sensor figures given in the source, not aggregated	Cross-sensor
MiniShift [81]	2026	12	2577	PC	Synth.	“each with 500,000 points” (abstract), a point count rather than a spacing	<1% anomalous area
IEC3D-AD [153]	2025	15	2400	PC	Real	None; “significantly improved point cloud resolution” is stated comparatively, with no figure (abstract)	Machined components
MulSen-AD [87]	2025	15	2035	RGB + PC + IR	Real	None; “high-resolution” with no figure, the three sensors named instead (abstract)	3-sensor fusion
Open-Industry [154]	2026	15	2400	PC	Real	None; “high-precision point clouds” is a premise about the field, not a property of this dataset (abstract)	Open-set defect types
Real-IAD MVN [155]	2026	–	–	RGB + Normals	Real	“surface normal maps from five distinct viewpoints” (abstract), a view count rather than a spacing	Dense surface normals
Industrial3D-AD [83]	2026	10	12,000	PC	Real	None; “a widely used industrial depth camera” with no figure (dataset section)	Sensor quantization noise; reflective finishes
IEC3D-IMAD [156]	2026	15	2400	PC	Real	None; the “micrometer-scale” figure (§I.B) describes the defects, not the acquisition	Defect area 0.51–2.64% of the cloud
ArtiAD [97]	2026	39	15,229	PC	Sim	None; point clouds are sampled from articulated models, not measured (abstract)	Articulated objects; joint-angle sweeps

### 12.6. Emerging Datasets

Twelve further datasets each attack a gap the five above leave open.

#### 12.6.1. Smaller Defects on Finer Scans

Real-IAD D3 [152] synchronizes RGB, micrometer-level point clouds and photometric-stereo pseudo-3D over 69 defect groups on components 7 to 27 mm long whose defects cover 0.46% to 6.39% of the surface. MiniShift [81] pushes harder, at 500,000 points per cloud and under 1% anomalous area, synthesized and released with the Simple3D detector. SiM3D [146] keeps only 333 instances but adds CAD models and voxel-level ground truth, so localization can be scored volumetrically with V-AUPRO. In IEC3D-IMAD [156], anomalous points make up 0.51% to 2.64% of a cloud, averaging 1.20%, so a detector can score well on an aggregate measure while missing the defect. Its own baselines show as much. M3DM with Point-MAE features reaches 94% object-level AUROC there, while PatchCore with FPFH reaches 52%, barely above chance, which the dataset’s authors read as evidence that micro-defect performance depends on the feature extractor.

#### 12.6.2. Harder Sensing Conditions

3D-ADAM [145] captures 217 additive-manufacturing parts with four industrial depth sensors and 27,346 annotated defect instances, the only benchmark in this corpus that permits cross-sensor evaluation. Industrial3D-AD [83] narrows to what an ordinary depth camera does badly, quantization noise and reflective finishes, so it tests robustness to acquisition rather than geometric precision. MulSen-AD [87] adds lock-in infrared thermography alongside RGB and a laser scanner, which reaches subsurface voids neither of the others can see. Real-IAD MVN [155] replaces the point cloud with dense multi-view surface normals, on the premise that normals preserve detail sparse sampling discards. PD-REAL [151] uses a consumer RealSense camera on handmade Play-Doh models, which buys the control of making one object with and without a chosen defect at the cost of realism, and Qin et al. [157] later use it for a multi-scale distillation method.

#### 12.6.3. Objects and Protocols the Earlier Benchmarks Assume Away

IEC3D-AD [153] covers gears, connectors, fasteners, and machined parts whose tolerance violations are surface deviations rather than the organic shapes Real3D-AD is built from. Open-Industry [154] changes the protocol instead of the objects, holding out defect categories unseen during training where every benchmark above assumes each defect type is known at test time. These two and IEC3D-IMAD follow one group’s acquisition protocol, 15 categories at 160 samples each, and release separate annotations and splits, but a method evaluated on all three has not been tested on three sensors or three part families. ArtiAD [97] breaks the assumption the whole section rests on, that a part is rigid and its pose can be canonicalized so that residual geometry after alignment reads as a defect. Its objects are joined by hinges and sliders, and each sample carries its joint configuration and part-level motion labels, so pose-induced geometry can be separated from structural defect. A seen/unseen articulation split test extrapolates to joint angles absent from training on simulated rather than captured data, the main caveat on it.

### 12.7. Discussion

MVTec 3D-AD established the standard evaluation protocols (Section 13), and the benchmarks that followed each attack a different limitation of it, from higher precision to pose-agnostic evaluation and high-resolution point clouds with subtle defects (MiniShift [81]), as Table 9 records and Figure S1 of the Supplementary Materials charts.

The remaining gaps are structural. Results from Eyecandies and Anomaly-ShapeNet do not transfer cleanly to real scans, and because nearly every benchmark uses small manufactured objects, neither large-scale industrial inspection nor medical imaging has a standard testbed. Cross-dataset protocols barely exist, and most methods are evaluated on one benchmark. Metric standardization, particularly for point-level and voxel-level scoring, is still in motion.

## 13. Experimental Comparison

### 13.1. Reading the Tables: Protocol, Metric, and Evidence Caveats

The tables collate results from the originating publications and mark no best value. Each number comes from its own paper’s protocol, preprocessing, metric subset, category coverage, and hardware, so no column is a controlled comparison and emphasis would assert a ranking the evidence does not support. Compare rows only where the protocol columns agree.

What a row is.

A row holds the highest value a paper reports for its own method under its headline configuration, not an ablation, a reduced-modality run, or a re-timed baseline. A value measured under a protocol the table does not compare is held out, with its reason, in Table S6 of the Supplementary Materials. Peer-review status is a column in the comparison tables, and Table 5 gives arXiv as the venue of a preprint; no preprint value is called a state of the art in this survey.

Protocol columns.

Each table states, per row, the training regime and normal references per category, the categories the reported mean covers, whether the pipeline registers or resamples the input before scoring, and where in the source the value was read. A field a paper does not state is printed as such a finding about the paper rather than a gap in this survey. Table S10 of the Supplementary Materials holds the full extraction.

Metric harmonization: what was mapped, and what was not.

(i) A sample-level AUROC is printed as I-AUC, Image AUROC, or AUROC and recorded as I-AUROC; a point-level P-AUC or Pixel AUROC is recorded as P-AUROC. (ii) On object-centric benchmarks a sample is a whole object, so an I-AUROC printed there, as by Template3D-AD [56] and PointAD+ [63] on Real3D-AD, is recorded as O-AUROC. (iii) A per-region overlap score is recorded as AUPRO only where the paper states the false-positive-rate limit it integrates to, printed beside the value; where both a 30% and a 1% limit are reported, as by CMSA [124], the 30% value is tabulated and the 1% is quoted in the text. (iv) A value called PRO with no stated limit is not treated as AUPRO and is marked so. Refused: no value is rescaled, re-evaluated, or averaged across papers; P-AUROC over pixels, and over points and V-AUPRO over voxels are kept apart even where the name is shared (Section 11.4); and two AUPRO values on point clouds are compared only where the connectivity rule they depend on is known to agree (Section 2.5). Table 4 lists the aliases and limits encountered.

Evidence tiers.

Table S7 of the Supplementary Materials grades every work on peer review, traceability of each value to a table or page in its source, and benchmark breadth. A preprint value is called a preprint value, and a within-paper comparison, the only controlled margin this survey can report, is attributed to the paper that ran it.

Cross-benchmark reading.

The benchmarks differ in modality, sample regime, object geometry and acquisition, and in what the metric names denote. A difference between an MVTec 3D-AD value and a Real3D-AD one is therefore not a measured performance gap; Section 13 uses it only to ask which assumptions each benchmark rewards.

### 13.2. Results on MVTec 3D-AD

Table 10 reports image-level AUROC (I-AUROC) for detection and pixel-level AUPRO for localization on MVTec 3D-AD [5].

#### 13.2.1. Evolution of Performance

The earliest methods on the benchmark (2021) achieved 50–70% I-AUROC, and the leading collated values now exceed 97%. Three changes account for most of it: pretrained 2D and 3D feature extractors replaced hand-built descriptors, multi-modal fusion brought RGB into a geometry problem, and scoring moved from single distances to dual memory banks and hybrid fusion.

#### 13.2.2. Method Category Analysis

Density estimation is thinly represented in Table 10, with two rows. HGAD [33] targets unified multi-class detection rather than the per-category 3D setting; MVFM-3DAD [54] reports object-level rather than image-level AUROC here; and the MVTec 3D-AD result of MC4AD [116] is an object-level AUROC on the benchmark’s 3D half, so none of the three is placed in the table. The family is best assessed on Real3D-AD (Table 11).

#### 13.2.3. Category-Level Variation

Texture-dominated categories (e.g., carrot, cookie) tend to yield higher AUROC for multi-modal methods, while geometry-dominated categories (e.g., foam, tire) with subtle surface deformations remain harder, above all for methods relying on 2D features. Small, localized defects are the hardest cases in the source papers’ qualitative evidence, though per-defect-size breakdowns are rarely reported, so this observation is indicative rather than measured.

### 13.3. Results on Real3D-AD

Real3D-AD [19] evaluates pure point cloud anomaly detection in a few-shot setting (Section 12), and the values in Table 11 are markedly lower than on MVTec 3D-AD.

The highest object-level value we collate on Real3D-AD comes from outside the registration family: MVFM-3DAD [54] reports 90.8% O-AUROC and 96.0% P-AUROC, several points clear of everything else in Table 11. It is a preprint and a single result, so we do not treat it as settling the benchmark, but it is the strongest evidence here that density estimation transfers to 3D (Section 7).

Registration and alignment-based methods otherwise dominate this benchmark, as template matching suits the high-precision point cloud format. Template3D-AD [56] leads the peer-reviewed subset (Section 8), and 3DKeyAD [57] leads point-level localization among registration methods (P-AUROC 86.1%) via keypoint-guided clustering. PASDF [50] follows at 80.2% O-AUROC with a pose-invariant implicit representation, and DUS-Net [102] at 79.5% through group-center-preserving down-up sampling; ISMP [135] reaches 76.7% by perceiving internal spatial structure and PO3AD [59] 76.5% by predicting per-point offsets. PointCore [71] improves on Reg3D-AD [19] by anchoring local features to global ones (Section 4), and MC3D-AD [18] is the one row that processes all categories with a single model (Section 5).

**Table 10:** Reported results on MVTec 3D-AD, each value the highest its paper reports under its own protocol; no column is a controlled comparison and no best value is marked (Section 13.1). *Status*: Pub. = peer-reviewed, Pre. = preprint. *Regime*: supervision and, below the full split, normal references per category. *Cat.*: categories the mean covers. *Class*: regime, category count and AUPRO limit as one code; rows sharing a code are comparable only if Pre-proc. agrees too. *Pre-proc.*: alignment before scoring (reg.) and resampling target (full description in Table S10 of the Supplementary Materials). A question mark marks an unstated field; an en dash an unreported value.

Method	Status	Input	Year	Regime	Cat.	Class	Pre-Proc.	I-AUROC	AUPRO^Limit^
3D-ST [51]	Pub.	PC	2023	one-class	10/10	OC/10/30	64,000 pts	–	83.3 ^30% and 1%^
AST [29]	Pub.	RGB+PC	2023	one-class	10/10	OC/10/–	not stated	93.7	–
BTF [23]	Pub.	RGB+PC	2023	one-class	10/10	OC/10/30	224 × 224	86.5	96.4 ^30%^
CPMF [17]	Pub.	PC	2023	one-class	10/10	OC/10/?	resampled	95.2	92.9 ^n.s.^
EasyNet [91]	Pub.	RGB-D	2023	synth. sup.	10/10	SS/10/?	yes	92.6	82.1 ^n.s.^
M3DM [22]	Pub.	RGB+PC	2023	one-class	10/10	OC/10/?	1024 × 128 groups	94.5	96.4 ^n.s.^
Shape-Guided [46]	Pub.	RGB+PC	2023	one-class	10/10	OC/10/30	500 pts	94.7	97.6 ^30% and 1%^
3DSR/DADA [48]	Pub.	RGB-D	2024	one-class	10/10	OC/10/?	yes	97.8	97.2 ^n.s.^
CFM [133]	Pub.	RGB+PC	2024	one-class	10/10	OC/10/30	1024 × 32 groups	95.4	97.1 ^30% and 1%^
DAS3D [37]	Pub.	RGB-D	2024	synth. sup.	10/10	SS/10/?	not stated	98.2	98.2 ^n.s.^
LSFA [84]	Pub.	RGB+PC	2024	one-class	10/10	OC/10/?	not stated	97.1	96.8 ^n.s.^
MMRD [53]	Pub.	RGB+PC	2024	one-class	10/10	OC/10/?	not stated	95.0	97.6 ^n.s.^
PointAD [20]	Pub.	PC	2024	zero-shot	9/10	ZS/9/?	not stated	82.0	84.4 ^n.s.^
2M3DF [65]	Pub.	RGB+PC	2025	one-class	10/10	OC/10/?	yes	96.6	97.5 ^n.s.^
CMSA [124]	Pub.	RGB+PC	2025	one-class	10/10	OC/10/30	1024 × 32 groups	98.4	97.6 ^30% and 1%^
DCMUNet [99]	Pub.	RGB-D	2025	synth. sup.	10/10	SS/10/?	not stated	94.1	96.9 ^n.s.^
HFMM-Net [132]	Pub.	RGB+PC	2025	one-class	10/10	OC/10/30	224 × 224	95.6	98.2 ^30% and 1%^
LPFSTNet [108]	Pub.	RGB-D	2025	one-class	10/10	OC/10/–	not stated	94.7	–
M3DM-NR [123]	Pub.	RGB+PC	2025	one-class	10/10	OC/10/?	128 pts	94.5	96.5 ^n.s.^
MAFR [127]	Pre.	RGB+PC	2025	one-class	10/10	OC/10/30	not stated	97.2	97.0 ^30% and 1%^
MCL-AD [64]	Pre.	RGB+PC	2025	zero-shot	10/10	ZS/10/?	yes	89.0	92.2 ^n.s.^
MMFlows [55]	Pub.	RGB+PC	2025	one-class	10/10	OC/10/?	resampled	93.3	96.1 ^n.s.^
PointAD+ [63]	Pre.	RGB+PC	2025	zero-shot	10/10	ZS/10/?	yes	87.7	93.1 ^n.s.^
UCF [128]	Pub.	RGB+PC	2025	one-class	10/10	OC/10/30	yes	96.3	97.5 ^30% and 1%^
V2G [98]	Pub.	RGB+PC	2025	one-class	10/10	OC/10/30	1024 × 32 groups	96.8	97.8 ^30%^
AEMG [85]	Pub.	RGB+PC	2026	one-class	10/10	OC/10/30	500 pts	95.6	94.4 ^30% and 1%^
CDRS [131]	Pub.	RGB+PC	2026	one-class	10/10	OC/10/?	yes	80.0	92.6 ^n.s.^
DCRDF-Net [109]	Pub.	RGB-D	2026	synth. sup.	10/10	SS/10/?	not stated	97.1	–
DGCF [125]	Pub.	RGB+PC	2026	one-class	10/10	OC/10/30	not stated	97.3	97.6 ^30%^
FMFR [126]	Pub.	RGB+PC	2026	one-class	10/10	OC/10/30	resampled	98.0	97.9 ^30% and 1%^
LM3M [130]	Pub.	RGB+PC	2026	one-class	10/10	OC/10/?	resampled	92.9	95.6 ^n.s.^
ZUMA [121]	Pub.	RGB+PC	2026	zero-shot	10/10	ZS/10/?	not stated	84.0	–

Input: PC = point cloud, RGB+PC = colour and point cloud, RGB-D = colour and depth. A superscript on a localization value is the false-positive-rate limit its paper states; *n.s.* marks a per-region score with no limit stated, not the same quantity (Section 13.1). Notes apply to Table 11 and Table 12 as well.

**Table 11:** Reported results on Real3D-AD, collated from the originating publications. Columns, codes and caveats as in Table 10 and Section 13.1.

Method	Status	Input	Year	Regime	Cat.	Class	Pre-Proc.	O-AUROC	P-AUROC
Reg3D-AD [19]	Pub.	PC	2023	few-shot, 4	12/12	FS/12	reg.; resampled	70.4	70.5
Group3AD [72]	Pub.	PC	2024	one-class, 4	12/12	OC/12	yes	75.4	–
IMRNet [49]	Pub.	PC	2024	–	12/12	?/12	yes	72.5	–
PointCore [71]	Pre.	PC	2024	one-class, 4	12/12	OC/12	reg.; resampled	82.9	73.1
R3D-AD [36]	Pub.	PC	2024	synth. sup.	12/12	SS/12	4096 pts	73.4	–
3DKeyAD [57]	Pub.	PC	2025	one-class, 4	12/12	OC/12	reg.; 20,000 pts	80.1	86.1
DUS-Net [102]	Pub.	PC	2025	one-class, 4	12/12	OC/12	8192 × 640 groups	79.5	84.7
ISMP [135]	Pub.	PC	2025	one-class, 4	12/12	OC/12	reg.; resampled	76.7	83.6
MC3D-AD [18]	Pub.	PC	2025	unified, 4	12/12	UNI/12	reg.; resampled	78.2	76.8
PASDF [50]	Pub.	PC	2025	one-class, 4	12/12	OC/12	reg.; resampled	80.2	–
PO3AD [59]	Pub.	PC	2025	one-class, 4	12/12	OC/12	resampled	76.5	–
PointAD+ [63]	Pre.	RGB+PC	2025	zero-shot	12/12	ZS/12	yes	83.5	76.0
Template3D-AD [56]	Pub.	PC	2025	few-shot, 1	12/12	FS/12	reg.; voxel0.25	84.4	–
LDSD [105]	Pre.	PC	2026	one-class, 4	12/12	OC/12	reg.; 100,000 pts	85.9	85.2
MVFM-3DAD [54]	Pre.	PC	2026	one-class	12/12	OC/12	not stated	90.8	96.0
MambaAD3D [89]	Pub.	PC	2026	synth. sup.	12/12	SS/12	resampled	78.4	76.6

**Table 12:** Reported results on Anomaly-ShapeNet, collated from the originating publications. Columns, codes and caveats as in Table 10 and Section 13.1.

Method	Status	Input	Year	Regime	Cat.	Class	Pre-Proc.	O-AUROC	P-AUROC
IMRNet [49]	Pub.	PC	2024	few-shot, 4	40/40	FS/40	30,000 pts	66.1	–
R3D-AD [36]	Pub.	PC	2024	synth. sup.	40/40	SS/40	2048 pts	74.9	–
DUS-Net [102]	Pub.	PC	2025	one-class	40/40	OC/40	8192 × 640 groups	79.7	71.2
MC3D-AD [18]	Pub.	PC	2025	unified	40/40	UNI/40	reg.; resampled	84.2	–
PASDF [50]	Pub.	PC	2025	one-class	40/40	OC/40	reg.; resampled	90.0	–
Template3D-AD [56]	Pub.	PC	2025	few-shot, 1	40/40	FS/40	reg.; voxel0.05	86.5	–
LDSD [105]	Pre.	PC	2026	one-class, 4	40/40	OC/40	reg.; 100,000 pts	92.1	92.4
MambaAD3D [89]	Pub.	PC	2026	synth. sup.	40/40	SS/40	resampled	86.8	91.2

### 13.4. Results on Anomaly-ShapeNet

Anomaly-ShapeNet [49] is the broadest of the three benchmarks (Section 12). In Table 12, PASDF [50] reports 90.0% O-AUROC, the highest peer-reviewed value there, and the highest overall is 92.1%, from the preprint LDSD. SeDiR [96] reports 93.3% and is not tabulated because that figure comes from a unified multi-category model, while this table compares one model per category. Template3D-AD [56] reaches 86.5%, which its authors report as the top score in 32 of 40 categories against the baselines then available. MC3D-AD [18] improves on prior single-category methods by 9.3% in its unified setting.

### 13.5. Cross-Benchmark Analysis

MVTec 3D-AD rewards paired RGB and organized 3D structure. Real3D-AD removes RGB, cuts the reference set to four samples, and requires full 360-degree geometric reasoning, which changes which families lead. Anomaly-ShapeNet broadens category coverage on synthetic defects.

Three patterns recur, charted as Figure S2 of the Supplementary Materials.

#### 13.5.1. Compression of Reported Values on MVTec 3D-AD

Reported values on MVTec 3D-AD have compressed toward the top of the scale. We say compression rather than saturation, because collated values cannot show that no method can improve on the benchmark. In Table 10, the top five fall within 1.1 I-AUROC points (CMSA 98.4% down to DGCF 97.3%), each under its own protocol. The heterogeneous protocols prevent attributing differences of this size to algorithmic improvements, and we have not quantified protocol variance, so we do not claim they fall inside it. Real3D-AD behaves differently: its top five span 7.9 O-AUROC points. Removing MVFM-3DAD at 90.8% leaves the next five spanning 5.7 points, and peer-reviewed work alone leaves 6.0, against 1.1 on MVTec 3D-AD in every case.

The contrast motivates the shift toward Real3D-AD, Anomaly-ShapeNet, and the high-resolution benchmarks of Section 12, though it licenses no ranking across them (Section 13.1).

#### 13.5.2. Multi-Modal Advantage, or Diverse-Feature Advantage?

Methods combining RGB and 3D data generally outperform single-modality approaches on MVTec 3D-AD, and M3DM’s own ablation confirms a substantial I-AUROC gain from adding RGB to its point-cloud branch. Yet CPMF [17], which uses *no* real RGB but renders the point cloud into multiple 2D views (“pseudo multi-modal”), is ahead of M3DM’s multi-modal pipeline on I-AUROC in Table 10, which suggests the gain is at least partly a *diverse-feature* advantage rather than a strict *modality* advantage. The large MVTec-vs-Real3D-AD gap is consistent with a dependence on 2D-derived features, though the benchmarks also differ in training-set size, precision, and viewpoint coverage (Section 12).

#### 13.5.3. Few-Shot Robustness

Memory banks built from only four references cover normal variation less well, which the source papers read as a source of false positives (Section 4); template matching reaches 84.4% O-AUROC from a single reference (Template3D-AD [56]).

#### 13.5.4. Scalability to Many Categories

The 40-category evaluation on Anomaly-ShapeNet shows most methods designed for 10–12 categories do not scale gracefully. Unified multi-category models [18] are one direction, and consistency across diverse geometries remains open.

### 13.6. Computational Cost as Reported

Earlier versions sorted methods into nominal speed bands. We removed them. A band implies the numbers in it are commensurable, and Table S11 of the Supplementary Materials, kept there because it is reference material a reader consults rather than reads, shows they are not: the figures come from different hardware, cover different parts of the pipeline, and use units the papers do not share.

That table reports, per method and quoted from its source, which pipeline stages the timing covers and whether the figure is model-only or end-to-end. Of 108 works examined for a runtime statement about their own method (the 98 inventory works whose source PDF was available and the 10 dataset papers), 79 state at least one figure and 29 state none (the table prints the 79). Hardware is given by 68, throughput or per-object latency by 44 and 27, batch size by 19, point count or resolution by 62, parameter count by 18, and memory by 34. 0 state every field a like-for-like comparison would need (all eight fields above, stated in one reported measurement). Preprocessing is the emptiest column: 14 works say their timed span includes it, and 4 time it separately, so no comparison of preprocessing cost is possible from the literature and none is attempted here.

No ranking by speed is available from this literature, and this survey does not offer one. The quantity an inspection cell is constrained by is absent almost everywhere: a cycle-time budget is per object and end-to-end, and 22 of the reporting works shows which their figure is. The 94.55 FPS of EasyNet [91] on a Tesla V100 is model-only throughput, excluding acquisition, preprocessing, transfer, and the decision step, so it shows that the network is not the bottleneck, not that the pipeline meets a line’s cycle time.

Memory-bank methods search a feature database, so inference cost grows with bank size and with the point count that populates it. Diffusion-based reconstruction runs an iterative denoising trajectory, which CM3D-AD [92] reduces to two consistency-model steps. Teacher–student inference is a pair of forward passes with no retrieval or iteration. Registration-based methods pay an alignment cost that no model-only figure captures. These differences are measured on a common footing nowhere in the corpus, and Section 15.3 treats what measuring them would need.

## 14. Deployment Contexts and Scope Boundaries

Industrial inspection is this survey’s context and the one its benchmarks were built for (Section 12); other domains, excluded from selection (Section 1), appear only as boundaries. Benchmarks feature small rigid manufactured objects under controlled conditions, with many normal references and a fixed pose. Each boundary case removes one of those four conditions, which a method evaluated where all four hold is not shown to survive.

### 14.1. Industrial Inspection

Industrial surface inspection drives this research [158,159], as every major benchmark reflects [5,19,141,152]. The task is to detect scratches, dents, contamination, and dimensional deviation on parts.

Geometric defects such as warping and concavities are often invisible in 2D projections but clear in 3D data. Structured-light sensors and laser scanners measure out-of-plane geometry directly, commonly to sub-millimeter depth resolution, though whether that supports a tolerance decision is the question of Section 11.4. The distinction is measurement modality rather than a resolution hierarchy, since 2D imaging often exceeds a 3D sensor in in-plane resolution. Real-IAD D3 [152] targets components of 7–27 mm at micrometer-level 3D resolution.

Deployment constraints include cycle-time budgets, robustness to lighting and vibration, and a false-positive rate low enough not to stop the line without cause. Section 15.3 treats how rarely the literature reports them. Few-shot results on Real3D-AD [19] address a real constraint, a new line without a large reference set.

Additive manufacturing fails differently [160], and 3D-ADAM defines 12 defect classes for printed parts, such as layer adhesion failures absent from subtractive manufacturing.

### 14.2. Boundary Cases

#### 14.2.1. Medical Volumetric Imaging

CT and MRI produce volumetric data in which pathology deviates from normal anatomy, so the one-class formulation of Section 2.2 applies structurally [161,162]. The assumption it breaks is that normality is a manufactured design’s specification with near-zero intended variance across a category, while anatomical normality is a population with large healthy inter-subject variation, so reference set and anomaly overlap as no industrial benchmark reproduces. Unsupervised autoencoder segmentation of brain MR [163] and training-free zero-shot detection [120] are established. We make no claim about which of the mechanisms in Section 3.1 suits clinical use, because we assessed no clinical evidence.

#### 14.2.2. Large-Scale LiDAR Scenes

Driving-scale point clouds run to millions of points per frame [164], against thousands to tens of thousands at the object level. A scene holds no single object of complete geometry, no template to register against, and no canonical pose, which removes the premises of the registration mechanism (Section 8) entirely rather than merely making it slower. Scalability is the visible problem [165]; the missing reference object is the structural one.

#### 14.2.3. Robotics and Bin Picking

Parts in a bin arrive at arbitrary orientation, so the broken assumption is pre-alignment [166]. It is the boundary case closest to the benchmarks and the one the field has responded to: PAD [142] makes pose-agnostic evaluation explicit, and pose handling is a cross-cutting dimension in Section 3.2. The benchmarks cannot tell us how much reported performance depends on that alignment.

### 14.3. Choosing a Method for an Operating Situation

For 11 operating situations, Table 13 states which method properties each implies, which works with collated evidence measured under it, and what that evidence leaves open. Each situation is fixed by four properties a practitioner knows in advance: input modality, normal references available at commissioning, how far pose varies, and whether a per-object cycle-time budget binds.

The table names method *properties* rather than families, because any scoring mechanism satisfying a requirement is admissible. Methods recur across rows, but none were evaluated across two situations under one protocol, so the rows cannot be read against each other (Section 13.1). The method lists are generated from the result ledger or from the inventory’s supervision field for the zero-shot row, as each cell states, so each cell traces to the supporting row in Section 13 and to its source.

Read the last column first. Two situations a production line meets routinely have almost no evidence: a hard cycle-time budget, where reported costs cover different pipeline stages on different hardware (Section 13.6), and an instrument change between commissioning and operation (Section 11.5). A guideline for ranking methods for those situations would be inventing the ranking.

## 15. Open Challenges and Future Directions

Progress on the benchmarks in Section 13 has been fast. The constraints below have not moved with it.

### 15.1. Evaluation Challenges

#### 15.1.1. Compressed Benchmark Values

Reported values on MVTec 3D-AD are compressed into a narrow band near the top of the scale (Section 13), which limits what a further gain on it can show unless the protocol behind it is stated.

#### 15.1.2. Protocol and Metric Inconsistency

Papers differ in metric subset, preprocessing, category coverage and ground-truth space. The tables of Section 13 therefore mark no best value and carry protocol columns, with the harmonization rules in Section 13.1.

#### 15.1.3. Missing Cross-Dataset Evaluation

Most methods are evaluated on a single benchmark [167].

#### 15.1.4. Evidence Status and Reproducibility

A substantial share of the leading reported values come from preprints, and mixing them with peer-reviewed results overstates what is established, so we mark the evidence tier explicitly (Section 1). Reproducible baselines and standardized evaluation suites remain overdue.

Code availability is weaker than the citation record suggests. Of 30 candidate repository links extracted from the surveyed papers and opened, 27 were the authors’ own, and 4 of those announced code that is not there. Announced-but-absent is the more consequential state, since a reader treats the announcement as a commitment. Table S9 gives the verdict per link and what each of the four announced-but-absent repositories holds. These were read in August 2026, and repository state is perishable.

### 15.2. Representation Challenges

#### 15.2.1. Weak Industrially Relevant 3D Pretraining

2D vision changed once large pretrained backbones became available [168], and 3D anomaly detection has no equivalent. Point-BERT [42] and Point-MAE [43] are both pretrained on ShapeNet [74], and neither approaches the scale or diversity behind comparable 2D models.

#### 15.2.2. Modality Dependence

Whether CPMF’s projected views (Section 13) substitute for a color sensor in general, or only where the benchmark’s defects are visible in projection, is unanswered and matters for production lines that capture geometry only.

#### 15.2.3. Sensor-Domain Shift

Representations are learned on one instrument and, in every benchmark but 3D-ADAM [145], evaluated on the same one, so robustness to a change of sensor principle, noise characteristic, or point density is largely unmeasured (Section 11.3). One line of work adapts at test time instead: TopoOT [169] freezes an existing detector and fits a segmentation head online on topology-aware optimal-transport pseudo-labels, and reports gains over frozen CFM, M3DM, and PO3AD backbones on MVTec 3D-AD and Anomaly-ShapeNet. It supplies no anomaly score of its own and is listed among the related techniques rather than with the detectors.

#### 15.2.4. Point-Native Foundation Models

Against the projection route of Section 9, point-native language alignment [66] and geometry-aware prompting [61,62] are the emerging alternative. Generative models trained on 3D data [93] are a further route, as reconstruction backbones and as synthesizers of anomalous training data, the scarcest resource in the field.

### 15.3. Deployment Challenges

#### 15.3.1. Pose and Correspondence

Most benchmarks fix the viewpoint; real inspection does not. Template- and alignment-based methods that tolerate arbitrary pose, ideally from a single reference and with rotation-invariant features, are a prerequisite for moving beyond curated data (Section 8).

#### 15.3.2. Acquisition Faults That Look Like Defects

Section 11 sets out three: extrinsic miscalibration, dropouts on reflective, transparent and dark surfaces, and the slow drift of an instrument between recalibrations. Each is confusable with the target signal, and none appears in any benchmark we identified.

#### 15.3.3. What Deployment Constraints Are Reported

Against the constraints of Section 14.1, what the literature reports is partial: model throughput is sometimes given, whole-pipeline latency almost never (Section 11.3), and a false-positive rate at a fixed operating point essentially never. Table S11 collects what the papers do report about cost.

#### 15.3.4. Latency

Industrial deployment works to a cycle-time budget, and no reported figure speaks to it directly (Section 13.6). Even the highest model-only throughput there, EasyNet’s [91], does not clear a 10 ms per-sample budget once acquisition and preprocessing are added. Distillation into compact architectures is one route, and the two-step consistency model of CM3D-AD [92] another (Section 5).

#### 15.3.5. Scale

Object-level methods are evaluated on thousands to tens of thousands of points. MiniShift and Simple3D [81] move to 500K-point clouds (Section 4 and Section 12), but nothing here has been tested at full scene scale.

#### 15.3.6. Few-Shot Commissioning

Real3D-AD’s four training samples per category [19] already produce marked degradation against data-rich benchmarks, and zero-shot methods [20,60] remain well below the supervised range, and prompting strategies are downstream of the pretraining gap above.

#### 15.3.7. Multi-Category and Open-World Operation

Maintaining one model per category does not scale to a flexible line. MC3D-AD’s unified model [18] is untested beyond tens of categories. Open-set evaluation exists for unseen *defect* types [154], but the open-world case, in which unseen *object* categories arrive without retraining, has no benchmark. C3D-AD [104] formalizes the continual setting, where categories arrive sequentially and forgetting must be mitigated.

#### 15.3.8. Uncertainty and Defect Characterization

An accept-or-reject decision needs a calibrated operating point that an unnormalized score and AUROC do not supply (Section 11.4). Where designs are proprietary, federated learning [170] is one route to shared models without exchanging raw scans.

### 15.4. Three Field-Defining Challenges

Most of the above would close with sufficient engineering effort. Three would not, because each would change what the field can claim rather than how well it performs:

1. **Distinguishing benchmark-specific progress from genuine generalization.** Until cross-dataset evaluation is routine, a rising number on a benchmark whose values have compressed cannot be separated from adaptation to one instrument’s view of one set of parts.
2. **Learned 3D representations that consistently outperform classical geometry under industrial sensing constraints.** The competitiveness of a 2009 descriptor marks the boundary of what learned 3D features currently deliver, and moving that boundary requires pretraining corpora that resemble industrial parts rather than everyday objects.
3. **Moving from binary detection to real-time defect characterization from few normal references.** Type, severity, dimensions, and a stated coverage interval on each are what an inspection decision consumes, and must be produced within a cycle-time budget from the handful of references a new line provides. Work has begun (Section 9), but it answers the *type* half from a fixed vocabulary and leaves severity, physical extent, and any statement of confidence untouched.

## 16. Conclusions

This survey covered 94 detectors and 8 related techniques for 3D anomaly detection, enumerated in Table S4 of the Supplementary Materials, spanning 2020 to 21 August 2026. Four findings follow from the collated evidence.

Six mutually exclusive scoring mechanisms and eight independent design dimensions (Section 3) place every one of the 94 detectors exactly once, the remaining 8 inventory works contributing no scoring mechanism, and the assignment follows the quantity each method thresholds rather than the vocabulary its paper prefers.

On MVTec 3D-AD, methods that combine color and geometry occupy the upper range of reported detection scores: the highest collated I-AUROC is 98.4% peer-reviewed and 97.2% in a preprint, each under its own protocol, and a top five within 1.1 points (Section 13.1) cannot say whether headroom is gone or the protocols vary.

The handcrafted-versus-learned question is open and benchmark-conditional (Section 4): FPFH holds on the color-paired MVTec 3D-AD, while on the geometry-only, few-shot Real3D-AD the best value, 90.8% O-AUROC from the preprint MVFM-3DAD against a peer-reviewed ceiling of 84.4%, is low enough on its own to say that high-precision geometric analysis from four reference samples is unsolved.

Three constraints would change what the field can claim rather than how well it performs, and Section 15 states them: cross-dataset evaluation routine enough to separate generalization from benchmark fitting, pretrained 3D encoders at the scale of their 2D counterparts, and speed and accuracy measured on one pipeline. Sensing conditions bound all of it: the acquisition characteristics that determine what is detectable are reported inconsistently or not at all (Section 11), and no work in this corpus states a coverage interval on any measurement. We intend this survey as a reference for what the reported evidence does and does not support.

## Figures and Tables

**Table 1:** The executed search by tier. Counts are records returned before de-duplication across queries, so they sum to more than the 1726 unique records screened. The queries themselves are given verbatim in the Supplementary Materials (Table S1), together with the 23 further queries executed as a test and not adopted (Section S2 there).

Tier	Queries	arXiv	OpenAlex	What the Tier Targets
1	8	91	293	Names the 3D task directly: the modality and the anomaly or defect term in one phrase.
2	8	444	2109	Widens to the surrounding task vocabulary, added after an earlier recovery test.
	16	535	2402	

**Table 2:** Positioning of this survey relative to existing anomaly detection surveys. ✓ = present, ∘ = partial/limited, — = absent. IAD = industrial anomaly detection; RUL = remaining useful life. “2D→3D” denotes an explicit analysis of which 2D paradigms transfer to 3D; “X-bench.” denotes consolidated cross-benchmark result tables. Entries reflect each survey’s stated scope.

Survey	Primary Focus	Organized by	2D → 3D	X-Bench.	’25–’26	Peer-Rev.
**This survey**	3D AD (PC/RGB-D/multi-modal)	Algorithmic paradigm	✓	✓ (verified)	✓	under review
Liang et al. [12]	3D AD	Method classification	∘	leaderboard list	✓	preprint
Rani et al. [10]	Point-cloud defect + RUL	Application	—	—	∘	✓
Lin et al. [9]	RGB/3D/multi-modal unsup. AD	Modality	∘	∘	∘	preprint
Du et al. [11]	3D vision AD in manufacturing	Application	—	∘	∘	✓
Liu et al. [6]	2D industrial AD	Method	—	2D only	—	✓
Cui et al. [7]	Unsupervised 2D IAD	Algorithm	—	2D only	—	✓
Hojjati et al. [8]	Self-supervised AD (CV)	Method	—	—	—	✓

**Table 3:** The 2D-to-3D paradigm-transfer lens: for each scoring mechanism of Axis A, plus the fusion dimension of Axis B, the 2D anomaly detection ancestor (if any), representative 3D instantiations, and our assessment of the transfer, justified in the referenced sections.

3D Mechanism or Dimension	2D Ancestor	3D Instantiations	Transfer Assessment
Feature embedding & memory bank (Section 4)	PatchCore, SPADE, PaDiM	BTF, M3DM, Shape-Guided	The 2D pipeline transfers intact. What limits it is the 3D backbone, not the matching step
Reconstruction (Section 5)	Autoencoders, DRAEM, 2D diffusion	IMRNet, R3D-AD, 3DSR/DADA	Transfers after two 3D-specific repairs: establishing set-to-set correspondence, and controlling the shortcut that lets a network copy its input
Teacher–student (Section 6)	STFPM, Reverse Distillation, EfficientAD	3D-ST, AST	Works on RGB-D. On raw point clouds, distillation still trails the other mechanisms
Density & likelihood estimation (Section 7)	CFlow-AD, FastFlow, CS-Flow	HGAD, VAE3D, MVFM-3DAD	Strong in 2D; one 2026 result under a comparable protocol in 3D, too little to judge (Section 7)
Multi-modal fusion (*dimension*) (Section 10)	— (RGB-only in the 2D IAD benchmarks considered here)	M3DM, CFM, DAS3D	No ancestor in the 2D anomaly-detection literature. These methods import 2D extractors as components and supply the fusion themselves
Registration & alignment (Section 8)	— (classical geometric vision, not 2D IAD)	Reg3D-AD, Template3D-AD, SplatPose	Inherited from geometric vision rather than from 2D anomaly detection. Pose variation is intrinsic in 3D, which promotes alignment from a preprocessing step to a scoring mechanism
Semantic & foundation-model transfer (Section 9)	AnomalyCLIP, AnomalyGPT	PointAD, MVP-PCLIP, BTP	Projection transfers the 2D model but discards geometry. Point-native language alignment is the alternative to it

**Table 4:** Summary of evaluation metrics for 3D anomaly detection, with the names under which the surveyed papers print each; the harmonization rules are in Section 13.1.

Metric	Level	Threshold-Free	Size-Independent	Primary Use	Printed as
I-AUROC/O-AUROC	Image/Object	✓	N/A	Detection	I-AUC, Image AUROC, AUROC
P-AUROC	Pixel/Point	✓	×	Localization	P-AUC, Pixel AUROC
AUPRO	Region	✓	✓	Localization	AUPRO@30%, AUPRO(0.3), P-AUPRO_0.3_, AUPRO@1%; “PRO” with no limit
F1-max	Image or Pixel	×	×	Detection/Localization	F1, max-F1 (not tabulated)
AP	Image or Pixel	✓	×	Detection/Localization	AP, mAP (not tabulated)
V-AUPRO	Voxel	✓	✓	3D Segmentation	V-AUPRO

✓ = yes, × = no, N/A = not applicable.

**Table 5:** Representative methods placed on both axes, grouped by primary scoring mechanism (Axis A). The Fusion, Supervision, Scope and Pose columns record positions on the cross-cutting axis (Axis B), and “2°” marks a genuine secondary mechanism.

Method	Venue	Year	Input	2°	Fus.	Sup.	Scope	Pose	Repr.	Key Idea
*(A1) Feature distance and memory matching*: 27 of 94 detectors, scores by Equation (2)										
BTF/3D-ADS [23]	CVPRW	2023	RGB+PC	–	F	OC	per	A	H+P2	FPFH + 2D features, memory bank
M3DM [22]	CVPR	2023	RGB+PC	–	F, D	OC	per	A	L3+P2	One memory bank per modality and scale
Shape-Guided [46]	ICML	2023	RGB+PC	–	F, D	OC	per	A	L3+IF	Dual-memory with shape priors
CPMF [17]	PR	2024	PC	–	F	OC	per	A	H+P2	Pseudo multi-modal from 3D alone
Reg3D-AD [19]	NeurIPS	2023	PC	A5	–	FS	per	R	L3	Registration + memory bank
MISDD [47]	IEEE T-Mech	2025	RGB+PC	A6	F	OC	per	A	P2	Prompts compensate a missing sensor
*(A2) Reconstruction and restoration residuals*: 25 of 94 detectors, scores by Equation (3)										
3DSR/DADA [48]	WACV	2024	RGB-D	–	F	Syn	per	A	L3	Depth-aware discrete autoencoder
R3D-AD [36]	ECCV	2024	PC	–	–	Syn	per	A	L3	Diffusion-based reconstruction
IMRNet [49]	CVPR	2024	PC	–	–	OC	per	A	L3	Iterative masked reconstruction
MC3D-AD [18]	IJCAI	2025	PC	–	–	OC	uni	A	H+L3	Reconstructs from a shared decoder
PASDF [50]	ICCV	2025	PC	–	–	OC	per	R	IF	Registers to a canonical frame, then SDF
*(A3) Teacher–student and representation discrepancy*: 13 of 94 detectors, scores by Equation (4)										
AST [29]	WACV	2023	RGB+PC	A4	D	OC	per	A	P2	Teacher and student differ in capacity
3D-ST [51]	WACV	2023	PC	–	–	OC	per	A	L3	Deep geometric descriptors
MDSS [52]	PRCV	2024	RGB+PC	–	D	OC	per	–	IF	Memoryless distillation + SDF
MMRD [53]	AAAI	2024	RGB+PC	–	F	OC	per	A	P2	Student restores a fused teacher target
*(A4) Density and likelihood estimation*: 5 of 94 detectors, scores by Equation (6)										
HGAD [33]	ECCV	2024	RGB+PC	–	F	OC	uni	–	P2	Hierarchical Gaussian mixture flow
VAE3D [34]	ICIP	2021	PC	–	–	OC	per	–	L3	Variational likelihood on point sets
MVFM-3DAD [54]	arXiv	2026	PC	–	–	OC	per	A	P2	Flow matching, multi-view density proxy
MMFlows [55]	J. Image Graph.	2025	RGB+PC	–	F	OC	per	A	L3+P2	Multi-scale flow over fused features
*(A5) Registration and template deviation*: 9 of 94 detectors, scores by Equation (7)										
Template3D-AD [56]	IJCAI	2025	PC	–	–	FS	per	R	H	Template matching via center points
3DKeyAD [57]	IEICE Trans.	2026	PC	A1	–	FS	per	R	H+L3	Keypoint-guided clustering
SplatPose [58]	CVPRW	2024	Multi-view	–	–	OC	per	PA	IF	Renders a splat model to compare views
PO3AD [59]	CVPR	2025	PC	A2	–	Syn	per	R	L3	Point-offset prediction
*(A6) Semantic and foundation-model transfer*: 15 of 94 detectors, scores by similarity in a pretrained semantic space (Section 9)										
PointAD [20]	NeurIPS	2024	PC	–	–	ZS	uni	A	P2	Scores points by CLIP text similarity
3DzAL [60]	WACV	2025	PC	–	–	ZS	uni	–	–	Contrasts patches against a learned prior
MVP-PCLIP [61]	IEEE TSMC	2025	PC	–	–	ZS	uni	–	P2	Multi-view projection + CLIP
GS-CLIP [62]	CVPR	2026	PC	–	–	ZS	uni	–	P2+IF	Gaussian-splat language alignment
PointAD+ [63]	arXiv	2025	RGB+PC	–	F	ZS	uni	A	P2	Point learning beside rendered views
MCL-AD [64]	arXiv	2025	RGB+PC	–	F	ZS	uni	A	P2	Point, image and text collaboration
*Related techniques (2D baselines, not counted among the 94 detectors)*										
AnomalyGPT [39]	AAAI	2024	RGB	–	–	ZS	uni	–	P2	Answers about defects in natural language
AnomalyCLIP [38]	ICLR	2024	RGB	–	–	ZS	uni	–	P2	Learns prompts that carry no object name

Input-modality abbreviations follow Section 2. PR = Pattern Recognition. Fusion: F = feature level, D = decision level, – = single modality. Supervision: OC = one-class, Syn = synthetic-anomaly supervised, FS = few-shot, ZS = zero-shot. Scope: per = one model per category, uni = unified. Pose: A = assumes aligned input, R = registers explicitly, PA = pose-agnostic. Repr.: H = handcrafted descriptors, L3 = learned 3D features, P2 = pretrained 2D features on projected views or images, IF = implicit or volumetric field, – = not stated in the released inventory. The Pose column records the regime a method was *evaluated* under, which for most entries follows from the benchmark (Section 3.2); where a paper states a pose property of its own, that statement governs. AnomalyGPT and AnomalyCLIP are 2D baselines included for context, carry no 3D pose regime, and are not counted among the detectors.

**Table 6:** Feature-distance and memory-matching methods for which a collated benchmark value exists. Detection and localization are separate columns, each naming its metric and benchmark. Group3AD’s empty localization cell means its paper reports no point-level value on Real3D-AD. The remaining methods of this section carry no collated value and appear in Table S4 of the Supplementary Materials instead; the rows are not a ranking, and the values are not comparable across benchmarks.

Method	Features	Detection	Localization
BTF [23]	FPFH+2D	86.5 I-AUROC (MVTec 3D-AD)	96.4 AUPRO (MVTec 3D-AD)
M3DM [22]	MAE+CNN	94.5 I-AUROC (MVTec 3D-AD)	96.4 AUPRO (MVTec 3D-AD)
Shape-G. [46]	Dual	94.7 I-AUROC (MVTec 3D-AD)	97.6 AUPRO (MVTec 3D-AD)
CPMF [17]	FPFH+Proj.	95.2 I-AUROC (MVTec 3D-AD)	92.9 AUPRO (MVTec 3D-AD)
LSFA [84]	Adapted 2D+3D	97.1 I-AUROC (MVTec 3D-AD)	96.8 AUPRO (MVTec 3D-AD)
Reg3D-AD [19]	MAE+Raw	70.4 O-AUROC (Real3D-AD)	70.5 P-AUROC (Real3D-AD)
PointCore [71]	Local-Global	82.9 O-AUROC (Real3D-AD)	73.1 P-AUROC (Real3D-AD)
Group3AD [72]	Group	75.4 O-AUROC (Real3D-AD)	

Metric definitions and their harmonization: Section 2.5 and Section 13.1. Values are as each paper reports them and are not comparable across benchmarks.

**Table 7:** Axis-A placement of the methods discussed in this section. Fusion is an Axis-B dimension, so every method here also occupies one of the six scoring mechanisms of Section 3.1; this table is the mapping, and Table S3 of the Supplementary Materials groups the full inventory the same way.

Scoring Mechanism (Axis A)	Methods
(A1) Feature distance and memory matching	CDRS, CMSA, G2SF, HFMM-Net, ISMP, LM3M, M3DM-NR, MulSen-AD
(A2) Reconstruction and restoration residuals	BridgeNet, DAS3D, DBRN, DGCF, FMFR, MAFR
(A3) Teacher–student and representation discrepancy	CFM, CMDIAD, CMDR-IAD, TRD, UCF
(A4) Density and likelihood estimation	2M3DF
(A6) Semantic and foundation-model transfer	Text-Guided, UniMF

**Table 13:** Operating situations, the method properties each implies, and the evidence this survey collates for it. The table names properties, not a method family: rows draw on different papers under different protocols, so a method in two rows has not been compared across them. Method lists and counts are generated from the result ledger of Section 13.

Situation	Modality	Normal Refs.	Pose	Latency	Implied Method Properties	Works with Collated Evidence	What That Evidence Does Not Establish
In-line inspection of a fixtured part, colour and geometry both captured	RGB + organized 3D	Hundreds	Fixed	Cycle time	Point-level output; feature-level or decision-level fusion of the two modalities; one model per category is affordable	highest collated I-AUROC 98.4% (CMSA, peer-reviewed); 33 works with a collated result; held out of the table on protocol grounds (Table S6): MC4AD 95.4%, MP3D 95.0%, UniMF 76.9%	Values here are compressed into 1.1 points across the top five, so the collated evidence does not separate the leading candidates; choose on the other columns
A new product line, geometry only, few reference parts, part presented in a repeatable pose	Point cloud	About four	Registrable	Softer	Few-shot supervision; explicit registration to a template, or pose-invariant descriptors	highest collated O-AUROC 90.8% (MVFM-3DAD, preprint); peer-reviewed ceiling 84.4% (Template3D-AD); 17 works with a collated result	The reported level is far below the RGB-paired case, and no work reports how it degrades with a different scanner
Many part families, geometry only, defect types known in advance	Point cloud	About four	Pre-aligned	Softer	Unified category scope; representations that transfer across geometry	highest collated O-AUROC 92.1% (LDSD, preprint); peer-reviewed ceiling 90.0% (PASDF); 8 works with a collated result; held out of the table on protocol grounds (Table S6): SeDiR 93.3%	Defects are synthetic and the objects are CAD-derived, so transfer to captured scans is untested here
Part arrives at arbitrary orientation, colour cameras only	Multi-view images	Many views	Arbitrary	Grasp cycle	Pose-agnostic scoring; geometry reconstructed from views rather than measured	highest collated I-AUROC 97.9% (R^3^-PAD, preprint); peer-reviewed ceiling 96.4% (SplatPose+); 3 works with a collated result	The benchmark’s objects are toy assemblies and its 3D is reconstructed, so metric geometric accuracy is not assessed
No normal samples available for the target category	Any	None	Assumed aligned	Softer	Zero-shot supervision; semantic or foundation-model transfer	13 works with a collated result (inventory, supervision regime)	Reported values sit well below the supervised range, and the gap is not closed by prompting
Articulated part whose joint configuration varies between samples	Point cloud	Per configuration	Articulated	Softer	Pose conditioning rather than canonicalization; the rigid prior fails	highest collated O-AUROC 88.4% (SPA-SDF, preprint); 1 work with a collated result	One simulated benchmark and one method, so this is an opening result rather than a basis for selection
The instrument may change between commissioning and operation	Point cloud or RGB-D	Any	Any	Any	Robustness to a change of sensor principle, noise characteristic and point density	none in this survey	No collated result exists. The practical route is to validate the candidate on 3D-ADAM, the one benchmark captured with four sensors, before commissioning; drift between recalibrations appears in no benchmark at all
A hard per-object cycle-time budget	Any	Any	Any	Hard	Mechanisms without retrieval or iterative inference; the whole pipeline, not the forward pass	70 works with a collated result (runtime table)	Reported cost covers different pipeline stages on different hardware, so no figure transfers: time the whole pipeline on the target hardware yourself (Section 13.6)
Medical volumetric imaging	CT or MR volume	A population, not a specification	Anatomical	Soft	Outside this survey’s evidence: normality is a population with large healthy variation, not a nominally identical part	none in this survey	No clinical evidence was assessed, so no mechanism is recommended or excluded (Section 14.2)
Large-scale LiDAR scenes	Scene geometry	No reference object	Unbounded	Hard, safety-critical	Rules out template registration; needs sub-linear scaling in point count	none in this survey	No object-level method here has been evaluated at scene scale; the premise of a reference object is absent (Section 14.2)
Robotic bin picking	RGB-D	Many	Arbitrary	Grasp cycle	Pose-agnostic or explicitly registering; tolerant of partial views	highest collated I-AUROC 97.9% (R^3^-PAD, preprint); peer-reviewed ceiling 96.4% (SplatPose+); 3 works with a collated result	Only the pose-agnostic benchmark speaks to it, and it is not a bin (Section 14.2)

## Data Availability

No new experimental data were generated. Every benchmark result reported here is reproduced from the originating publication and attributed there. The complete executed search, all 16 queries with their per-database yields, is given verbatim as Table S1 of the Supplementary Materials, so the identification stage can be re-executed from the article and its supplement. Everything derived from the search is released with the article: the query set, the extraction schema, the complete inventory, the full-text exclusions, the protocol measurements and the per-value source locations are the Supplementary Materials listed above. The raw screening and eligibility logs those artifacts are derived from are Supplementary File S3. The scripts that execute the protocol and generate every count in this survey are available from the corresponding author on request.

## Supplementary Materials

The following supporting information can be downloaded at https://www.mdpi.com/article/10.3390/s26216717/s1, Supplementary File S1 (PDF): Section S1, the executed search, with every query verbatim (Table S1); Section S2, the widened-search test and its full query set (Table S2); Section S3, the extraction schema (Table S3); Section S4, the complete inventory of 94 detectors and 8 related techniques with venue and peer-review status (Table S4); Section S5, every record excluded at full text (Table S5); Section S6, the protocol measurements, including the single-reviewer limitation; Section S7, the audit of the collated results, covering the values held out on protocol grounds (Table S6) and the evidence-strength grading (Table S7); Section S8, the source-location ledger giving where in its originating publication each reported value was read (Table S8); and Section S9, the per-work code-availability verdicts (Table S9); Section S10, extended descriptions of the 25 methods the article names once and does not use again; Section S11, charted views of data the article tabulates (Figures S1 and S2); Section S12, the evaluation protocol behind every tabulated result, in each source’s own wording and with where it was read (Table S10); Section S13, what each surveyed work reports about computational cost (Table S11); and Section S14, every experiment in the corpus that varies a sensing condition and reports the effect (Table S12). Supplementary File S2: the same inventory as a machine-readable table, in CSV and XLSX, one row per work and one column per charted field. Supplementary File S3 (ZIP): the three extraction tables behind Tables S10–S12, the result ledger, and the raw search, screening, chaining and eligibility logs, as CSV. Reference numbers in Supplementary File S1 are those of this article’s reference list. References [171,172,173,174,175,176,177,178,179,180,181,182,183,184,185,186] are cited in the Supplementary Materials.

## References

[1] Bergmann P., Fauser M., Sattlegger D., Steger C. MVTec AD—A Comprehensive Real-World Dataset for Unsupervised Anomaly Detection. Proceedings of the IEEE/CVF Conference on Computer Vision and Pattern Recognition (CVPR).

[2] Roth K., Pemula L., Zepeda J., Schölkopf B., Brox T., Gehler P. Towards Total Recall in Industrial Anomaly Detection. Proceedings of the IEEE/CVF Conference on Computer Vision and Pattern Recognition (CVPR).

[3] Defard T., Setkov A., Loesch A., Audigier R. (2021). PaDiM: A Patch Distribution Modeling Framework for Anomaly Detection and Localization. Proceedings of the International Conference on Pattern Recognition (ICPR) Workshops.

[4] Zavrtanik V., Kristan M., Skočaj D. DRAEM: A Discriminatively Trained Reconstruction Embedding for Surface Anomaly Detection. Proceedings of the IEEE/CVF International Conference on Computer Vision (ICCV).

[5] Bergmann P., Jin X., Sattlegger D., Steger C. The MVTec 3D-AD Dataset for Unsupervised 3D Anomaly Detection and Localization. Proceedings of the International Conference on Computer Vision Theory and Applications (VISAPP).

[6] Liu J., Xie G., Wang J., Li S., Wang C., Zheng F., Jin Y. (2024). Deep Industrial Image Anomaly Detection: A Survey. Mach. Intell. Res..

[7] Cui Y., Liu Z., Lian S. (2023). A Survey on Unsupervised Anomaly Detection Algorithms for Industrial Images. IEEE Access.

[8] Hojjati H., Ho T.K.K., Armanfard N. (2024). Self-Supervised Anomaly Detection in Computer Vision and Beyond: A Survey and Outlook. Neural Netw..

[9] Lin Y., Chang Y., Tong X., Yu J., Liotta A., Huang G., Song W., Zeng D., Wu Z., Wang Y. (2024). A Survey on RGB, 3D, and Multimodal Approaches for Unsupervised Industrial Anomaly Detection. arXiv.

[10] Rani A., Ortiz-Arroyo D., Durdevic P. (2024). Advancements in Point Cloud-Based 3D Defect Detection and Classification for Industrial Systems: A Comprehensive Survey. arXiv.

[11] Du J., Tao C., Cao X., Tsung F. (2025). 3D Vision-Based Anomaly Detection in Manufacturing: A Survey. Front. Eng. Manag..

[12] Liang H., Guo B., Huang Y., Lyu J., Gao C., Cao Y., Wang J., Yu R., Shen L., Li P. (2025). 3D Anomaly Detection: A Survey. https://www.researchgate.net/publication/398334588_3D_Anomaly_Detection_A_Survey?channel=doi&linkId=6931d2157e61d05b530bc812&showFulltext=true.

[13] Qi C.R., Su H., Mo K., Guibas L.J. PointNet: Deep Learning on Point Sets for 3D Classification and Segmentation. Proceedings of the IEEE/CVF Conference on Computer Vision and Pattern Recognition (CVPR).

[14] Qi C.R., Yi L., Su H., Guibas L.J. PointNet++: Deep Hierarchical Feature Learning on Point Sets in a Metric Space. Proceedings of the Advances in Neural Information Processing Systems (NeurIPS).

[15] Choy C., Gwak J., Savarese S. 4D Spatio-Temporal ConvNets: Minkowski Convolutional Neural Networks. Proceedings of the IEEE/CVF Conference on Computer Vision and Pattern Recognition (CVPR).

[16] Wu Z., Song S., Khosla A., Yu F., Zhang L., Tang X., Xiao J. 3D ShapeNets: A Deep Representation for Volumetric Shapes. Proceedings of the IEEE/CVF Conference on Computer Vision and Pattern Recognition (CVPR).

[17] Cao Y., Xu X., Shen W. (2024). Complementary Pseudo Multimodal Feature for Point Cloud Anomaly Detection. Pattern Recognit..

[18] Cheng J., Gao C., Zhou J., Wen J., Dai T., Wang J. MC3D-AD: A Unified Geometry-aware Reconstruction Model for Multi-Category 3D Anomaly Detection. Proceedings of the International Joint Conference on Artificial Intelligence (IJCAI).

[19] Liu J., Xie G., Chen R., Li X., Wang J., Liu Y., Wang C., Zheng F. Real3D-AD: A Dataset of Point Cloud Anomaly Detection. Proceedings of the Advances in Neural Information Processing Systems (NeurIPS), Datasets and Benchmarks Track.

[20] Zhou Q., Yan J., He S., Meng W., Chen J. PointAD: Comprehending 3D Anomalies from Points and Pixels for Zero-shot 3D Anomaly Detection. Proceedings of the Advances in Neural Information Processing Systems (NeurIPS).

[21] Cohen N., Hoshen Y. (2020). Sub-Image Anomaly Detection with Deep Pyramid Correspondences. arXiv.

[22] Wang Y., Peng J., Zhang J., Yi R., Wang Y., Wang C. Multimodal Industrial Anomaly Detection via Hybrid Fusion. Proceedings of the IEEE/CVF Conference on Computer Vision and Pattern Recognition (CVPR).

[23] Horwitz E., Hoshen Y. Back to the Feature: Classical 3D Features Are (Almost) All You Need for 3D Anomaly Detection. Proceedings of the CVPR Workshop on Visual Anomaly and Novelty Detection (VAND).

[24] Liu Z., Zhou Y., Xu Y., Wang Z. SimpleNet: A Simple Network for Image Anomaly Detection and Localization. Proceedings of the IEEE/CVF Conference on Computer Vision and Pattern Recognition (CVPR).

[25] You Z., Cui L., Shen Y., Yang K., Lu X., Zheng Y., Le X. A Unified Model for Multi-class Anomaly Detection. Proceedings of the Advances in Neural Information Processing Systems (NeurIPS).

[26] Wang G., Han S., Ding E., Huang D. Student-Teacher Feature Pyramid Matching for Anomaly Detection. Proceedings of the British Machine Vision Conference (BMVC).

[27] Deng H., Li X. Anomaly Detection via Reverse Distillation from One-Class Embedding. Proceedings of the IEEE/CVF Conference on Computer Vision and Pattern Recognition (CVPR).

[28] Batzner K., Heckler L., K"onig R. EfficientAD: Accurate Visual Anomaly Detection at Millisecond-Level Latencies. Proceedings of the IEEE/CVF Winter Conference on Applications of Computer Vision (WACV).

[29] Rudolph M., Wehrbein T., Rosenhahn B., Wandt B. Asymmetric Student-Teacher Networks for Industrial Anomaly Detection. Proceedings of the IEEE/CVF Winter Conference on Applications of Computer Vision (WACV).

[30] Gudovskiy D., Ishizaka S., Kozuka K. CFLOW-AD: Real-Time Unsupervised Anomaly Detection with Localization via Conditional Normalizing Flows. Proceedings of the IEEE/CVF Winter Conference on Applications of Computer Vision (WACV).

[31] Yu J., Zheng Y., Wang X., Li W., Wu Y., Zhao R., Wu L. (2021). FastFlow: Unsupervised Anomaly Detection and Localization via 2D Normalizing Flows. arXiv.

[32] Rudolph M., Wehrbein T., Rosenhahn B., Wandt B. Fully Convolutional Cross-Scale-Flows for Image-Based Defect Detection. Proceedings of the IEEE/CVF Winter Conference on Applications of Computer Vision (WACV).

[33] Yao X., Li R., Qian Z., Wang L., Zhang C. Hierarchical Gaussian Mixture Normalizing Flow Modeling for Unified Anomaly Detection. Proceedings of the European Conference on Computer Vision (ECCV).

[34] Masuda M., Hachiuma R., Fujii R., Saito H., Sekikawa Y. Toward Unsupervised 3D Point Cloud Anomaly Detection using Variational Autoencoder. Proceedings of the IEEE International Conference on Image Processing (ICIP).

[35] Li C.L., Sohn K., Yoon J., Pfister T. CutPaste: Self-Supervised Learning for Anomaly Detection and Localization. Proceedings of the IEEE/CVF Conference on Computer Vision and Pattern Recognition (CVPR).

[36] Zhou Z., Wang L., Fang N., Wang Z., Qiu L., Zhang S. R3D-AD: Reconstruction via Diffusion for 3D Anomaly Detection. Proceedings of the European Conference on Computer Vision (ECCV).

[37] Li K., Dai B., Fu J., Hou X. DAS3D: Dual-modality Anomaly Synthesis for 3D Anomaly Detection. Proceedings of the Computer Vision—ECCV 2024 Workshops.

[38] Zhou Q., Pang G., Tian Y., He S., Chen J. AnomalyCLIP: Object-agnostic Prompt Learning for Zero-shot Anomaly Detection. Proceedings of the 12th International Conference on Learning Representations (ICLR).

[39] Gu Z., Zhu B., Zhu G., Chen Y., Tang M., Wang J. AnomalyGPT: Detecting Industrial Anomalies Using Large Vision-Language Models. Proceedings of the AAAI Conference on Artificial Intelligence (AAAI).

[40] Rusu R.B., Blodow N., Beetz M. Fast Point Feature Histograms (FPFH) for 3D Registration. Proceedings of the IEEE International Conference on Robotics and Automation (ICRA).

[41] Wang Y., Sun Y., Liu Z., Sarma S.E., Bronstein M.M., Solomon J.M. (2019). Dynamic Graph CNN for Learning on Point Clouds. ACM Trans. Graph..

[42] Yu X., Tang L., Rao Y., Huang T., Zhou J., Lu J. Point-BERT: Pre-Training 3D Point Cloud Transformers with Masked Point Modeling. Proceedings of the IEEE/CVF Conference on Computer Vision and Pattern Recognition (CVPR).

[43] Pang Y., Wang W., Tay F.E., Liu W., Tian Y., Yuan L. Masked Autoencoders for Point Cloud Self-supervised Learning. Proceedings of the European Conference on Computer Vision (ECCV).

[44] Zhao H., Jiang L., Jia J., Torr P., Koltun V. Point Transformer. Proceedings of the IEEE/CVF International Conference on Computer Vision (ICCV).

[45] Vaswani A., Shazeer N., Parmar N., Uszkoreit J., Jones L., Gomez A.N., Kaiser Ł., Polosukhin I. Attention Is All You Need. Proceedings of the Advances in Neural Information Processing Systems (NeurIPS).

[46] Chu Y.M., Liu C., Hsieh T.I., Chen H.T., Liu T.L. Shape-Guided Dual-Memory Learning for 3D Anomaly Detection. Proceedings of the International Conference on Machine Learning (ICML).

[47] Jiang S., Ma Y., Zhou J., Bian Y., Wang Y., Liu M. (2025). Resilient Multimodal Industrial Surface Defect Detection With Uncertain Sensors Availability. IEEE/ASME Trans. Mechatron..

[48] Zavrtanik V., Kristan M., Skočaj D. Cheating Depth: Enhancing 3D Surface Anomaly Detection via Depth Simulation. Proceedings of the IEEE/CVF Winter Conference on Applications of Computer Vision (WACV).

[49] Li W., Xu X., Gu Y., Zheng B., Gao S., Wu Y. Towards Scalable 3D Anomaly Detection and Localization: A Benchmark via 3D Anomaly Synthesis and A Self-Supervised Learning Network. Proceedings of the IEEE/CVF Conference on Computer Vision and Pattern Recognition (CVPR).

[50] Zheng B., Gan J., Xu X., Chen X., Li W., Huang X., Ni N., Wu Y. Bridging 3D Anomaly Localization and Repair via High-Quality Continuous Geometric Representation. Proceedings of the IEEE/CVF International Conference on Computer Vision (ICCV).

[51] Bergmann P., Sattlegger D. Anomaly Detection in 3D Point Clouds Using Deep Geometric Descriptors. Proceedings of the IEEE/CVF Winter Conference on Applications of Computer Vision (WACV).

[52] Sun Z., Li X., Li Y., Ma Y. Memoryless Multimodal Anomaly Detection via Student-Teacher Network and Signed Distance Learning. Proceedings of the Pattern Recognition and Computer Vision (PRCV 2024).

[53] Gu Z., Zhang J., Liu L., Chen X., Peng J., Gan Z., Jiang G., Shu A., Wang Y., Ma L. Rethinking Reverse Distillation for Multi-Modal Anomaly Detection. Proceedings of the AAAI Conference on Artificial Intelligence (AAAI).

[54] Li L., Liu L., Zhang J., Du X., Hao R., Li X., Liang H., Liu J. (2026). MVFM-3DAD: Multi-view Flow Matching for 3D Anomaly Detection via Density Proxy Estimation. arXiv.

[55] Qu H., Lin J. (2025). Multimodal multiscale industrial anomaly detection via flows. J. Image Graph..

[56] Liu Y., Zhang C., Yang Y. Template3D-AD: Point Cloud Template Matching Method Based on Center Points for 3D Anomaly Detection. Proceedings of the International Joint Conference on Artificial Intelligence (IJCAI).

[57] Wang Z., Hotta K., Kamide K., Zou Y., Zhang C., Yu J. (2026). 3DKeyAD: High-Resolution 3D Point Cloud Anomaly Detection via Keypoint-Guided Point Clustering. IEICE Trans. Inf. Syst..

[58] Kruse M., Rudolph M., Woiwode D., Rosenhahn B. SplatPose & Detect: Pose-Agnostic 3D Anomaly Detection. Proceedings of the CVPR Workshop on Visual Anomaly and Novelty Detection (VAND).

[59] Ye J., Zhao W., Yang X., Cheng G., Huang K. PO3AD: Predicting Point Offsets toward Better 3D Point Cloud Anomaly Detection. Proceedings of the IEEE/CVF Conference on Computer Vision and Pattern Recognition (CVPR).

[60] Wang Y., Peng K.C., Fu Y. Towards Zero-shot 3D Anomaly Localization. Proceedings of the IEEE/CVF Winter Conference on Applications of Computer Vision (WACV).

[61] Cheng Y., Cao Y., Xie G., Lu Z., Shen W. (2026). Toward Zero-Shot Point Cloud Anomaly Detection: A Multiview Projection Framework. IEEE Trans. Syst. Man Cybern. Syst..

[62] Deng Z., Liu A., Wang Y. GS-CLIP: Zero-shot 3D Anomaly Detection by Geometry-Aware Prompt and Synergistic View Representation Learning. Proceedings of the IEEE/CVF Conference on Computer Vision and Pattern Recognition (CVPR).

[63] Zhou Q., He S., Yan J., Meng W., Chen J. (2025). PointAD+: Learning Hierarchical Representations for Zero-shot 3D Anomaly Detection. arXiv.

[64] Li G., Chen T., Zhou M., Li M., Han D., Wan J. (2025). MCL-AD: Multimodal Collaboration Learning for Zero-Shot 3D Anomaly Detection. arXiv.

[65] Asad M., Azeem W., Jiang H., Mustafa H.T., Yang J., Liu W. (2025). 2M3DF: Advancing 3D Industrial Defect Detection With Multi-Perspective Multimodal Fusion Network. IEEE Trans. Circuits Syst. Video Technol..

[66] Li K., Li G., Zhou M., Li M., Han D., Wan J. Back to Point: Exploring Point-Language Models for Zero-Shot 3D Anomaly Detection. Proceedings of the IEEE/CVF Conference on Computer Vision and Pattern Recognition (CVPR).

[67] Xiang A., Huang Z., Gao X., Ye K., Xu C.z. BridgeNet: A Unified Multimodal Framework for Bridging 2D and 3D Industrial Anomaly Detection. Proceedings of the ACM International Conference on Multimedia (ACM MM).

[68] Cheng Y., Du J. (2025). 3D-PNAS: 3D Industrial Surface Anomaly Synthesis with Perlin Noise. arXiv.

[69] Sun Y., Cheng Y., Zu J., Tan Y., Xie G., Wang Y., Cao Y., Shen W. (2026). Synthesis4AD: Synthetic Anomalies are All You Need for 3D Anomaly Detection. arXiv.

[70] Wang Y., Xu X., Liu J., Lei X., Xie G., Jiang G., Lu Z. (2025). A Survey on Industrial Anomalies Synthesis. arXiv.

[71] Zhao B., Xiong Q., Zhang X., Guo J., Liu Q., Xing X., Xu X. (2024). PointCore: Efficient Unsupervised Point Cloud Anomaly Detector Using Local-Global Features. arXiv.

[72] Zhu H., Xie G., Hou C., Dai T., Gao C., Wang J., Shen L. Towards High-resolution 3D Anomaly Detection via Group-Level Feature Contrastive Learning. Proceedings of the ACM International Conference on Multimedia (MM).

[73] Chen T., Kornblith S., Norouzi M., Hinton G. A Simple Framework for Contrastive Learning of Visual Representations. Proceedings of the 37th International Conference on Machine Learning (ICML).

[74] Chang A.X., Funkhouser T., Guibas L., Hanrahan P., Huang Q., Li Z., Savarese S., Savva M., Song S., Su H. (2015). ShapeNet: An Information-Rich 3D Model Repository. arXiv.

[75] Uy M.A., Pham Q.H., Hua B.S., Nguyen D.T., Yeung S.K. Revisiting Point Cloud Classification: A New Benchmark Dataset and Classification Model on Real-World Data. Proceedings of the IEEE/CVF International Conference on Computer Vision (ICCV).

[76] Radford A., Kim J.W., Hallacy C., Ramesh A., Goh G., Agarwal S., Sastry G., Askell A., Mishkin P., Clark J. Learning Transferable Visual Models From Natural Language Supervision. Proceedings of the 38th International Conference on Machine Learning (ICML).

[77] Yu Y., Chen Z., Xu X., Zhang L., Yang H., Nie Y., He S. Registration is a Powerful Rotation-Invariance Learner for 3D Anomaly Detection. Proceedings of the Advances in Neural Information Processing Systems (NeurIPS).

[78] Liang H., Zhou J., Gao C., Guo B., Wang J., Shen L. (2026). A Lightweight 3D Anomaly Detection Method with Rotationally Invariant Features. Pattern Recognit..

[79] Cheng Y., Cao Y., Wang D., Shen W., Li W. (2025). Boosting Global-Local Feature Matching via Anomaly Synthesis for Multi-Class Point Cloud Anomaly Detection. IEEE Trans. Autom. Sci. Eng..

[80] Fichtel L., Seuß D., Timofte R. Anomaly-to-Normal Translation: A Training Paradigm for Feature-Based 3D Point Cloud Anomaly Detection. Proceedings of the CVPR Workshop on Visual Anomaly and Novelty Detection (VISION26).

[81] Cheng Y., Sun Y., Zhang H., Shen W., Cao Y. Towards High-Resolution 3D Anomaly Detection: A Scalable Dataset and Real-Time Framework for Subtle Industrial Defects. Proceedings of the AAAI Conference on Artificial Intelligence (AAAI).

[82] Liang H., Zhou J., Chen X., Dai T., Wang J., Gao C. (2025). Fence Theorem: Towards Dual-Objective Semantic-Structure Isolation in Preprocessing Phase for 3D Anomaly Detection. arXiv.

[83] Hoang D.C., Tan P.X., Nguyen A.N., Le M., Duong T.H.A., Huynh T.M., Nguyen D.M., Cao M.D., Ngo D.H., Vu M.Q. (2026). Efficient Industrial Point Cloud Anomaly Detection via Spatial Context Aggregation and Selective Anomalous Feature Generation. Sci. Rep..

[84] Tu Y., Zhang B., Liu L., Li Y., Chen X., Zhang J., Wang Y., Wang C., Zhao C.R. Self-supervised Feature Adaptation for 3D Industrial Anomaly Detection. Proceedings of the European Conference on Computer Vision (ECCV).

[85] Liu S., Luan X., Li Y. (2026). Multimodal Industrial Anomaly Detection via Attention-Enhanced Memory-Guided Network. IEEE Trans. Multimed..

[86] Liu S., Luan X., Li Y. (2026). APG-Net: Adaptive Prototype Guidance Network for Multi-Sensor Industrial Anomaly Detection. IEEE Trans. Circuits Syst. Video Technol..

[87] Li W., Zheng B., Xu X., Gan J., Lu F., Li X., Ni N., Tian Z., Huang X., Gao S. Multi-Sensor Object Anomaly Detection: Unifying Appearance, Geometry, and Internal Properties. Proceedings of the IEEE/CVF Conference on Computer Vision and Pattern Recognition (CVPR).

[88] Liu Y., Zhang C., Dong X., Yang Y. (2026). A 3D anomaly detection method based on multiscale point features for defects in vehicle stamped parts. J. Manuf. Process..

[89] Hoang D.C., Tan P.X., Nguyen A.N., Ngo D.H., Cao M.D., Vu M.Q. (2026). Multi-scale token fusion with Mamba adapters for efficient 3D industrial defect detection. Array.

[90] Kingma D.P., Welling M. Auto-Encoding Variational Bayes. Proceedings of the 2nd International Conference on Learning Representations (ICLR).

[91] Chen R., Xie G., Liu J., Wang J., Luo Z., Wang J., Zheng F. EasyNet: An Easy Network for 3D Industrial Anomaly Detection. Proceedings of the ACM International Conference on Multimedia (MM).

[92] Pranav A., B S., Siddappa P., Seuss D., Moharir M., KN S. Two Steps Are All You Need: Efficient 3D Point Cloud Anomaly Detection with Consistency Models. Proceedings of the IEEE/CVF Conference on Computer Vision and Pattern Recognition (CVPR) Workshops.

[93] Ho J., Jain A., Abbeel P. Denoising Diffusion Probabilistic Models. Proceedings of the Advances in Neural Information Processing Systems (NeurIPS).

[94] Song Y., Sohl-Dickstein J., Kingma D.P., Kumar A., Ermon S., Poole B. Score-Based Generative Modeling through Stochastic Differential Equations. Proceedings of the 9th International Conference on Learning Representations (ICLR).

[95] van den Oord A., Vinyals O., Kavukcuoglu K. Neural Discrete Representation Learning. Proceedings of the Advances in Neural Information Processing Systems (NeurIPS).

[96] Kim S., Lee W., Cho M. A Semantically Disentangled Unified Model for Multi-category 3D Anomaly Detection. Proceedings of the IEEE/CVF Conference on Computer Vision and Pattern Recognition (CVPR).

[97] Gan J., Zheng B., Xu X., Ren J., Zhang Z., Ni N., Wu Y. (2026). Breaking the Rigid Prior: Towards Articulated 3D Anomaly Detection. arXiv.

[98] Hoang D.C., Tan P.X., Nguyen A.N., Tran D.T., Duong V.H., Mai A.T. (2025). Unsupervised Visual-to-Geometric Feature Reconstruction for Vision-Based Industrial Anomaly Detection. IEEE Access.

[99] Tang K., Li Y., Zi B., Feng K. (2025). A Multimodal Unsupervised Coating Defect Detection Method Based on Dual-Branch Hybrid CNN-Mamba U-Net. IEEE Trans. Instrum. Meas..

[100] Bi C., Li Y., Luo H. Dual-Branch Reconstruction Network for Industrial Anomaly Detection with RGB-D Data. Proceedings of the International Conference on Image, Signal Processing, and Pattern Recognition (ISPP).

[101] Lhoste R., Vacavant A., Delhay D. MAESTRO: A Full Point Cloud Approach for 3D Anomaly Detection Based on Reconstruction. Proceedings of the International Conference on Computer Vision Theory and Applications (VISAPP).

[102] Liang H., Zhang J., Dai T., Shen L., Wang J., Gao C. Taming Anomalies with Down-Up Sampling Networks: Group Center Preserving Reconstruction for 3D Anomaly Detection. Proceedings of the 33rd ACM International Conference on Multimedia (MM).

[103] Li Z., Ge Y., Wang X., Meng L. (2024). 3D Industrial Anomaly Detection via Dual Reconstruction Network. Appl. Intell..

[104] Lu H., Liang H., Zhang J., Hu C., Wang J., Gao C. (2025). C3D-AD: Toward Continual 3D Anomaly Detection via Kernel Attention with Learnable Advisor. arXiv.

[105] Xiao H., Liang H., Zhou J., Wang J., Gao C. (2026). Learning Discriminative Signed Distance Functions from Multi-scale Level-of-detail Features for 3D Anomaly Detection. arXiv.

[106] Hinton G., Vinyals O., Dean J. (2015). Distilling the Knowledge in a Neural Network. arXiv.

[107] Liu X., Wang J., Leng B., Zhang S. (2026). Tuned Reverse Distillation: Enhancing Multimodal Industrial Anomaly Detection with Crossmodal Tuners. IEEE Trans. Multimed..

[108] Cheng Y., Chen J., Wen G., Tan X., Liu X. (2025). LPFSTNet: A lightweight and parameter-free head attention-based student–teacher network for fast 3D industrial anomaly detection. Neurocomputing.

[109] Wang C., Chen J., Zhang H. (2026). DCRDF-Net: A Dual-Channel Reverse-Distillation Fusion Network for 3D Industrial Anomaly Detection. Sensors.

[110] He K., Zhang X., Ren S., Sun J. Deep Residual Learning for Image Recognition. Proceedings of the IEEE Conference on Computer Vision and Pattern Recognition (CVPR).

[111] Dosovitskiy A., Beyer L., Kolesnikov A., Weissenborn D., Zhai X., Unterthiner T., Dehghani M., Minderer M., Heigold G., Gelly S. An Image is Worth 16x16 Words: Transformers for Image Recognition at Scale. Proceedings of the 9th International Conference on Learning Representations (ICLR).

[112] Rezende D.J., Mohamed S. Variational Inference with Normalizing Flows. Proceedings of the 32nd International Conference on Machine Learning (ICML).

[113] Papamakarios G., Nalisnick E., Rezende D.J., Mohamed S., Lakshminarayanan B. (2021). Normalizing Flows for Probabilistic Modeling and Inference. J. Mach. Learn. Res..

[114] Dinh L., Sohl-Dickstein J., Bengio S. Density Estimation Using Real-NVP. Proceedings of the 5th International Conference on Learning Representations (ICLR).

[115] Fischler M.A., Bolles R.C. (1981). Random Sample Consensus: A Paradigm for Model Fitting with Applications to Image Analysis and Automated Cartography. Commun. ACM.

[116] Liang H., Wang A., Zhou J., Jin X., Gao C., Wang J. (2025). Examining the Source of Defects from a Mechanical Perspective for 3D Anomaly Detection. arXiv.

[117] Wang Y., Yi R., Wang X., Hao J., Wang Y., Fan K., Chen Y., Zhang Z., Jiang G., Zhu W. (2026). Pose-Agnostic Anomaly Detection with Retinex-based Illumination Alignment. SSRN.

[118] Kirillov A., Mintun E., Ravi N., Mao H., Rolland C., Gustafson L., Xiao T., Whitehead S., Berg A.C., Lo W.Y. Segment Anything. Proceedings of the IEEE/CVF International Conference on Computer Vision (ICCV).

[119] Oquab M., Darcet T., Moutakanni T., Vo H., Szafraniec M., Khalidov V., Fernandez P., Haziza D., Massa F., El-Nouby A. (2024). DINOv2: Learning Robust Visual Features without Supervision. Trans. Mach. Learn. Res..

[120] Le-Gia T., Ahn J. (2026). Training-Free Zero-Shot Anomaly Detection in 3D Brain MRI with 2D Foundation Models. arXiv.

[121] Ma Y., Liu M., Jiang S., Zhou J., Bian Y., Wang X., Wang Y. (2026). ZUMA: Training-Free Zero-Shot Unified Multimodal Anomaly Detection. IEEE Trans. Pattern Anal. Mach. Intell..

[122] Huang Z., Song Y., Zhang H. Where & What Anomaly? A Framework for Pose-Agnostic Anomaly Detection and Zero-Shot Semantic Classification. Proceedings of the International Conference on Multimedia Retrieval (ICMR).

[123] Wang C., Zhu H., Peng J., Wang Y., Yi R., Wu Y., Ma L., Zhang J. (2025). M3DM-NR: RGB-3D Noisy-Resistant Industrial Anomaly Detection via Multimodal Denoising. IEEE Trans. Pattern Anal. Mach. Intell..

[124] Li B., Zhang T., Zuo Z., Wu Z. Cross-Modal Semantic Alignment for Efficient Unsupervised Multimodal Anomaly Detection. Proceedings of the Frontiers in Artificial Intelligence and Applications (ECAI).

[125] Lee T., Jeong S., Lee S.J. (2026). Discrepancy-Guided Complementary Fusion for Unsupervised Multimodal Anomaly Detection. Sensors.

[126] Wang C., Zhang H. (2026). FMFR: Feature-level multi-stage fusion and remapping for multi-modal industrial anomaly detection. J. Comput. Des. Eng..

[127] Ali U., Zia A., Rehman A., Ramzan U., Hassan Z., Sattar T., Wang J., Xiang W. (2025). 2D-3D Feature Fusion via Cross-Modal Latent Synthesis and Attention Guided Restoration for Industrial Anomaly Detection. arXiv.

[128] Cheng H., Luo J., Zhang X. (2025). Multimodal Industrial Anomaly Detection via Uni-Modal and Cross-Modal Fusion. IEEE Trans. Ind. Inform..

[129] Jiang S., Ma Y., Zhou J., Wang Y., Liu M. (2026). Unified Multimodal Industrial Anomaly Detection via Few Normal Samples. IEEE Trans. Ind. Inform..

[130] Zhang H. (2026). A Lightweight Multi-3D-Memory Extension for RGB-3D Industrial Anomaly Detection via Transformer Compression. IEEE Access.

[131] Xu J., Xiu P., Shi K., Xu L., Wang H. (2026). Cross-Modal Disagreement-Guided Reliability-Aware Scoring for RGB-3D Industrial Anomaly Detection. Appl. Sci..

[132] Zhao G., Tan L., He M., Wu Q. (2025). HFMM-Net: A Hybrid Fusion Mamba Network for Efficient Multimodal Industrial Defect Detection. Information.

[133] Costanzino A., Zama Ramirez P., Lisanti G., Di Stefano L. Multimodal Industrial Anomaly Detection by Crossmodal Feature Mapping. Proceedings of the IEEE/CVF Conference on Computer Vision and Pattern Recognition (CVPR).

[134] Long K., Xie G., Ma L., Liu J., Lu Z. Revisiting Multimodal Fusion for 3D Anomaly Detection from an Architectural Perspective. Proceedings of the AAAI Conference on Artificial Intelligence (AAAI).

[135] Liang H., Xie G., Hou C., Wang B., Gao C., Wang J. Look Inside for More: Internal Spatial Modality Perception for 3D Anomaly Detection. Proceedings of the AAAI Conference on Artificial Intelligence (AAAI).

[136] Salvi J., Fernandez S., Pribanic T., Llado X. (2010). A state of the art in structured light patterns for surface profilometry. Pattern Recognit..

[137] Blais F. (2004). Review of 20 years of range sensor development. J. Electron. Imaging.

[138] Sansoni G., Trebeschi M., Docchio F. (2009). State-of-the-Art and Applications of 3D Imaging Sensors in Industry, Cultural Heritage, Medicine, and Criminal Investigation. Sensors.

[139] Foix S., Alenya G., Torras C. (2011). Lock-in Time-of-Flight (ToF) Cameras: A Survey. IEEE Sens. J..

[140] Remondino F., El-Hakim S. (2006). Image-based 3D Modelling: A Review. Photogramm. Rec..

[141] Bonfiglioli L., Toschi M., Silvestri D., Fioraio N., De Gregorio D. The Eyecandies Dataset for Unsupervised Multimodal Anomaly Detection and Localization. Proceedings of the Asian Conference on Computer Vision (ACCV).

[142] Zhou Q., Li W., Jiang L., Wang G., Zhou G., Zhang S., Zhao H. PAD: A Dataset and Benchmark for Pose-agnostic Anomaly Detection. Proceedings of the Advances in Neural Information Processing Systems (NeurIPS), Datasets and Benchmarks Track.

[143] (2012). International Vocabulary of Metrology—Basic and General Concepts and Associated Terms.

[144] (2008). Evaluation of Measurement Data—Guide to the Expression of Uncertainty in Measurement.

[145] McHard P., Audonnet F.P., Summerell O., Andraos S., Henderson P., Aragon-Camarasa G. (2025). 3D-ADAM: A Dataset for 3D Anomaly Detection in Additive Manufacturing. arXiv.

[146] Costanzino A., Zama Ramirez P., Lella L., Ragaglia M., Oliva A., Lisanti G., Di Stefano L. SiM3D: Single-instance Multiview Multimodal and Multisetup 3D Anomaly Detection Benchmark. Proceedings of the IEEE/CVF International Conference on Computer Vision (ICCV).

[147] Wang J., Wu M. (2026). ActiveInspect: GRPO-Optimized Multi-Sensor Evidence Selection for Industrial Defect Detection. Sensors.

[148] Zeng J., Meng Y., Huang J. (2026). Defect inspection of highly reflective objects using adaptive-projection structured light and PointNet++. Opt. Express.

[149] Mildenhall B., Srinivasan P.P., Tancik M., Barron J.T., Ramamoorthi R., Ng R. NeRF: Representing Scenes as Neural Radiance Fields for View Synthesis. Proceedings of the European Conference on Computer Vision (ECCV).

[150] Liu Y., Hu Y.S., Chen Y., Zelek J. SplatPose+: Real-time Image-Based Pose-Agnostic 3D Anomaly Detection. Proceedings of the Computer Vision—ECCV 2024 Workshops.

[151] Qin J., Gu C., Yu J., Zhang C. PD-REAL: A 3D Anomaly Detection Dataset with Play-Doh and RealSense. Proceedings of the 7th Asian Conference on Artificial Intelligence Technology (ACAIT).

[152] Zhu W., Wang L., Zhou Z., Wang C., Pan Y., Zhang R., Chen Z., Cheng L., Gao B.B., Zhang J. Real-IAD D3: A Real-World 2D/Pseudo-3D/3D Dataset for Industrial Anomaly Detection. Proceedings of the IEEE/CVF Conference on Computer Vision and Pattern Recognition (CVPR).

[153] Guo B., Li H., Yu R., Liang H., Wang J. (2026). IEC3D-AD: A 3D Dataset of Industrial Equipment Components for Unsupervised Point Cloud Anomaly Detection. IEEE Trans. Circuits Syst. Video Technol..

[154] Liang H., Zhang L., Xia J., Zhou H., Guo B., Xie Y., Gao C., Yu R., Wang J., Li P. (2026). Open-Set Supervised 3D Anomaly Detection: An Industrial Dataset and a Generalisable Framework for Unknown Defects. arXiv.

[155] Zhu W., Liang J., Cheng L., Pan Y., Chen Z., Yan Q., Cheng Y., Zhang J., Chi M., Peng B. (2026). Real-IAD MVN: A Multi-View Normal Vector Dataset and Benchmark for High-Fidelity Industrial Anomaly Detection. arXiv.

[156] Wang M., Yue M., Guo B., Yu R. IEC3D-IMAD: A Real-World 3D Dataset of Industrial Equipment Components for Imbalanced Micro Anomaly Detection. Proceedings of the 41st Youth Academic Annual Conference of Chinese Association of Automation (YAC).

[157] Qin J., Zhang C., Gu C., Wang Z., Yu J., Wei Y., Xiao H., Yu X. (2023). Multi-Scale Distillation for RGB-D Anomaly Detection on the PD-REAL Dataset. arXiv.

[158] Czimmermann T., Ciuti G., Milazzo M., Chiurazzi M., Roccella S., Oddo C.M., Dario P. (2020). Visual-Based Defect Detection and Classification Approaches for Industrial Applications—A Survey. Sensors.

[159] Tabernik D., Šela S., Skvarč J., Skočaj D. (2020). Segmentation-Based Deep-Learning Approach for Surface-Defect Detection. J. Intell. Manuf..

[160] Scime L., Beuth J. (2018). Anomaly Detection and Classification in a Laser Powder Bed Additive Manufacturing Process Using a Trained Computer Vision Algorithm. Addit. Manuf..

[161] Litjens G., Kooi T., Bejnordi B.E., Setio A.A.A., Ciompi F., Ghafoorian M., van der Laak J.A.W.M., van Ginneken B., Sánchez C.I. (2017). A Survey on Deep Learning in Medical Image Analysis. Med. Image Anal..

[162] Fernando T., Gammulle H., Denman S., Sridharan S., Fookes C. (2022). Deep Learning for Medical Anomaly Detection—A Survey. ACM Comput. Surv..

[163] Baur C., Denner S., Wiestler B., Navab N., Albarqouni S. (2021). Autoencoders for Unsupervised Anomaly Segmentation in Brain MR Images: A Comparative Study. Med. Image Anal..

[164] Bogdoll D., Nitsche M., Zöllner J.M. Anomaly Detection in Autonomous Driving: A Survey. Proceedings of the IEEE/CVF Conference on Computer Vision and Pattern Recognition Workshops (CVPRW).

[165] Kosel M., Schreiber M., Ulrich M., Glaser C., Dietmayer K. Revisiting Out-of-Distribution Detection in LiDAR-based 3D Object Detection. Proceedings of the IEEE Intelligent Vehicles Symposium (IV).

[166] Park D., Erickson Z., Bhattacharjee T., Kemp C.C. Multimodal Execution Monitoring for Anomaly Detection During Robot Manipulation. Proceedings of the IEEE International Conference on Robotics and Automation (ICRA).

[167] Zhou K., Liu Z., Qiao Y., Xiang T., Loy C.C. (2022). Domain Generalization: A Survey. IEEE Trans. Pattern Anal. Mach. Intell..

[168] Bommasani R., Hudson D.A., Adeli E., Altman R., Arora S., von Arx S., Bernstein M.S., Bohg J., Bosselut A., Brunskill E. (2022). On the Opportunities and Risks of Foundation Models. arXiv.

[169] Zia A., Ali U., Ramzan U., Rehman A., Khamis A., Xiang W. (2026). Test-Time Adaptation for Anomaly Segmentation via Topology-Aware Optimal Transport Chaining. arXiv.

[170] McMahan B., Moore E., Ramage D., Hampson S., Arcas B.A.y. Communication-Efficient Learning of Deep Networks from Decentralized Data. Proceedings of the 20th International Conference on Artificial Intelligence and Statistics (AISTATS).

[171] Bhunia A.K., Li C., Bilen H. Looking 3D: Anomaly Detection with 2D-3D Alignment. Proceedings of the IEEE/CVF Conference on Computer Vision and Pattern Recognition (CVPR).

[172] Daci R., Renò V., Patruno C., Cardellicchio A., Taleb-Ahmed A., Leo M., Distante C. (2026). Cross-Modal Mapping and Dual-Branch Reconstruction for 2D-3D Multimodal Industrial Anomaly Detection. arXiv.

[173] Hoang D.C., Tan P.X., Nguyen A.N., Duong T.H.A., Huynh T.M., Nguyen M.A., Nguyen D.M., Cao M.D., Ngo D.H., Vu M.Q. (2026). Vote3D-AD: Unsupervised Point Cloud Anomaly Localization via Varied Defect Synthesis and Differentiable Vote-Clustering. Alex. Eng. J..

[174] Kerbl B., Kopanas G., Leimkühler T., Drettakis G. (2023). 3D Gaussian Splatting for Real-Time Radiance Field Rendering. ACM Trans. Graph..

[175] Li Z., Ye S., Yu Z., Xie W., Shen L. (2026). Text-Guided Multimodal Unified Industrial Anomaly Detection. arXiv.

[176] Marchi E., Fučka M., Skočaj D., Foresti G.L. (2026). Unsupervised Pose-Agnostic Visual Anomaly Detection in Realistic Industrial Scenes. Integr.-Comput.-Aided Eng..

[177] Qin J., Gu C., Yu J., Zhang C. (2023). Teacher-Student Network for 3D Point Cloud Anomaly Detection with Few Normal Samples. Expert Syst. Appl..

[178] Sui W., Lichau D., Lefèvre J., Phelippeau H. (2026). Incomplete Multimodal Industrial Anomaly Detection via Cross-Modal Distillation. Inf. Fusion.

[179] Tao C., Cao X., Du J. G2SF: Geometry-Guided Score Fusion for Multimodal Industrial Anomaly Detection. Proceedings of the IEEE/CVF International Conference on Computer Vision (ICCV).

[180] Tian C., Pang S., Du A. (2025). Hierarchical Asymmetric Student-Teacher Framework for 3D Point Cloud Anomaly Detection. Proceedings of the Neural Information Processing (ICONIP), Auckland, New Zealand, 2–6 December 2024.

[181] Wang J., Xu H., Chen X., Xu H., Huang Y., Ding X., Tu X. (2026). Exploiting Point-Language Models with Dual-Prompts for 3D Anomaly Detection. Expert Syst. Appl..

[182] Wang Z., Hotta K., Kamide K., Zou Y., Qin J., Zhang C., Yu J. (2026). DMP-3DAD: Cross-Category 3D Anomaly Detection via Realistic Depth Map Projection with Few Normal Samples. IEICE Trans. Inf. Syst..

[183] Wu L., Zou Q., Yue Y., Wang Z. Geometry-Aligned and Anomaly-Aware Reconstruction for 3D Anomaly Detection. Proceedings of the IEEE/CVF Conference on Computer Vision and Pattern Recognition (CVPR).

[184] Wu L., Zou Q., Lu J., Li Q. (2026). Point Cloud Diffusion with Global and Local Reconstruction for Instance-Level 3D Anomaly Detection. arXiv.

[185] Xu K., Wang X., Hou Y., Ma X., Xie J., Qiu J. (2026). CoGeoAD: Hierarchical Color-Geometric Fusion with Multi-View Attention for Zero-Shot 3D Anomaly Detection. arXiv.

[186] Zuo Z., Dong J., Wu Y., Qu Y., Wu Z. (2024). CLIP3D-AD: Extending CLIP for 3D Few-Shot Anomaly Detection with Multi-View Images Generation. arXiv.

