# Supplementary material for: 3D Anomaly Detection for Sensor-Based Industrial Inspection: A Critical Survey of Methods, Benchmarks, and Deployment Constraints
Source: Sensors (Basel). 2026 Oct 23;26(21):6717. doi: 10.3390/s26216717 (PMC13765651; doi:10.3390/s26216717)
Supplement: Supplementary file 1 [file sensors-26-06717-s001.zip › supplementary-file-s1-Sensors_fichtel_22_10_sup.pdf]

Supplementary Materials: 3D Anomaly Detection for Sensor-Based Industrial Inspection: A Critical Survey of Methods, Benchmarks, and Deployment Constraints

Lars Fichtel [1,2,*], Radu Timofte [2] and Dominik Seuß [1,3]

This document accompanies the article named above. It holds the material that supports the survey's claims without forming part of its argument: the search as executed, the schema by which every work was charted, the complete inventory of methods with peer-review status, the records excluded at full text, the measurements taken against the protocol, and the extended descriptions of methods discussed only briefly in the article. Section and table numbers prefixed with S refer to this document; unprefixed numbers refer to the article.

S1. The Executed Search

Table S1 gives every query the reported search executed, verbatim, with what each returned from either database. Section 1 summarises the same search by tier. The strings are here rather than there because a reader re-executing the identification stage needs them exactly, and a reader following the argument does not.

Table S1. The search as executed. Every query is a phrase match over title and abstract, restricted to work published from 2020 onward. Tier 1 targets the 3D task directly; tier 2 widens to the surrounding task vocabulary and was added after an earlier recovery test, which is why its recovery figure is not uniformly held out (Section S6). Counts are records per database before de-duplication across queries, so they sum to more than the 1,726 unique records screened; queries returning nothing are listed rather than dropped. A further 23 queries were executed as a test (Section S2).

Query	Tier	arXiv	OpenAlex
`3D anomaly detection`	1	62	153
`point cloud anomaly detection`	1	13	39
`point cloud defect detection`	1	0	3
`3D defect detection`	1	2	42
`RGB-D anomaly detection`	1	1	2
`depth map anomaly detection`	1	0	0
`multimodal industrial anomaly detection`	1	13	54
`3D industrial inspection anomaly`	1	0	0
`industrial anomaly detection`	2	187	967
`surface anomaly detection`	2	24	192
`multimodal anomaly detection`	2	28	172
`zero-shot anomaly detection`	2	79	285
`few-shot anomaly detection`	2	65	253
`unified anomaly detection`	2	17	97
`anomaly synthesis`	2	40	134
`3D anomaly localization`	2	4	9

S2. The Widened-Search Test

The search reported in Section 1 is the 16 queries frozen on 21 August 2026. It retrieves 93 of the 102 works in our inventory, and Section 1 attributes part of the shortfall to phrase matching rather than to what the databases index. That attribution is testable, so we tested it.

On 23 August 2026 we executed 23 further queries, taking the set to 39. Table S2 lists every query executed. They were derived from the frozen grid rather than from the missed works: reading tiers 1 and 2 as task phrase by qualifier shows eleven qualifiers crossed with "anomaly detection" and two with "defect detection". R1 completes that cross for

the modality and domain qualifiers, R2 adds the orthographic variant of each compound this literature writes two ways, and R3 extends the two task phrases that carried almost no qualifiers. The list was committed before the search ran.

Recovery rose from 93 to 96 of 102: the widened set reaches MISDD, MMRD, ActiveInspect, which the 16 frozen queries do not. It added no work to the corpus, and it screened 6,162 unique records to do it, 4,436 more than the reported search. Roughly nine in ten of those additions are two-dimensional surface-inspection literature that the modality screen cannot exclude, because its term list contains the very word the query searched for.

The decisive result is not the precision cost but the redundancy. Citation chaining, which this protocol already requires, recovers MISDD, MMRD, ActiveInspect on its own. Both searches therefore end at the same place: Shape-Guided, LCN, AARD are absent under the 16-query protocol and Shape-Guided, LCN, AARD under the 39-query one. Widening the vocabulary moved no endpoint. It reached earlier, by a more expensive route, works that a required stage of the protocol reaches anyway.

We therefore report the 16-query search as the protocol and this as a negative result. The vocabulary hypothesis is not wrong: those 3 works are reachable by phrase and the frozen queries were not reaching them. It is simply not worth acting on, because the stage that compensates for it is already in the protocol and costs 4,436 fewer records to run.

Table S2. Every query executed, including the 16 reported in Section 1; tier 3 is the widened set tested here, each query labeled with the rule that produced it. Counts are records returned by each database before de-duplication across queries. Queries that returned nothing are listed rather than dropped, since a rule applied only where it pays is not a rule.

Query	Tier	Rule	arXiv	OpenAlex
`3D anomaly detection`	1	frozen	62	153
`point cloud anomaly detection`	1	frozen	13	39
`point cloud defect detection`	1	frozen	0	3
`3D defect detection`	1	frozen	2	43
`RGB-D anomaly detection`	1	frozen	1	2
`depth map anomaly detection`	1	frozen	0	0
`multimodal industrial anomaly detection`	1	frozen	13	54
`3D industrial inspection anomaly`	1	frozen	0	0
`industrial anomaly detection`	2	frozen	187	978
`surface anomaly detection`	2	frozen	24	192
`multimodal anomaly detection`	2	frozen	28	173
`zero-shot anomaly detection`	2	frozen	79	287
`few-shot anomaly detection`	2	frozen	65	254
`unified anomaly detection`	2	frozen	17	97
`anomaly synthesis`	2	frozen	40	135
`3D anomaly localization`	2	frozen	4	10
`surface defect detection`	3	R1	74	4,164
`multimodal defect detection`	3	R1	0	20
`industrial defect detection`	3	R1	41	669
`RGB-D defect detection`	3	R1	0	1
`depth map defect detection`	3	R1	0	0
`multi-modal industrial anomaly detection`	3	R2	3	11
`multi-modal anomaly detection`	3	R2	8	63
`multi-modal defect detection`	3	R2	1	12
`multi-modal anomaly localization`	3	R2	0	0
`multi-modal anomaly synthesis`	3	R2	1	1
`RGBD anomaly detection`	3	R2	0	1
`RGBD defect detection`	3	R2	0	0
`RGBD anomaly localization`	3	R2	0	0
`RGBD anomaly synthesis`	3	R2	0	0
`point cloud anomaly localization`	3	R3	0	1
`RGB-D anomaly localization`	3	R3	0	0
`depth map anomaly localization`	3	R3	0	0
`multimodal anomaly localization`	3	R3	0	0
`3D anomaly synthesis`	3	R3	2	4
`point cloud anomaly synthesis`	3	R3	0	0
`RGB-D anomaly synthesis`	3	R3	0	0
`depth map anomaly synthesis`	3	R3	0	0
`multimodal anomaly synthesis`	3	R3	0	0

S3. Extraction Schema

Table S3 lists the 18 fields recorded for every work in the released inventory. The field list is read from the inventory file itself, so a column cannot be added to the data without being described here.

Two fields are marked as human verdicts and never extracted. Both were attempted by extraction first and both mis-attributed values, so they are typed from a reading of the source and the extraction path was removed rather than repaired.

Table S3. Extraction schema: the 18 fields recorded per work, what each records, and where its value comes from.

Field	Records	Source of the value
`method`	Short name used throughout the survey.	Assigned by us; the authors' name where they give one.
`publication_title`	Title of the originating publication.	Bibliography entry, checked against the source.
`version_status_date`	Date the peer-review status was checked.	Recorded when the status was verified.
`peer_review_status`	Peer-reviewed or preprint.	Publisher or repository record.

Field	Records	Source of the value
venue	Publication venue, abbreviated.	Bibliography entry, checked against the source.
doi	DOI where one exists.	Publisher record.
arxiv_id	arXiv identifier where one exists.	arXiv.
input_modality	Sensor data the method consumes.	Stated by the paper.
sensor_representation	Geometric representation the method operates on.	Human verdict; never extracted.
supervision	Supervision regime.	Stated by the paper.
primary_scoring_mechanism	Axis-A position: what produces the anomaly score.	Assigned by reading what the paper says produces its score.
fusion_type	Axis-B position: at what level modalities combine.	Stated by the paper, blank where single-modality.
pose_assumption	Pose regime the method was evaluated under.	Follows from the benchmark, or a property the paper states. Blank where neither supports a value.
benchmark	Benchmarks the work reports on.	Reported results table.
metric	Metrics reported.	Reported results table.
source_table_or_page	Where in the source each number was read.	Recorded during verification.
code_availability	Repository link, or a note that none exists.	Human verdict; never extracted.
verified_on	Date the numeric values were checked against the source.	Recorded during verification.

S4. Complete Method Inventory

Table S4 enumerates the 94 dedicated 3D anomaly detectors surveyed in this work together with 8 related techniques, grouped by primary paradigm, so that the coverage claim is fully auditable. The related techniques are listed in a separate block at the end and are deliberately *not* counted among the 94 detectors. What they share is that none contributes an anomaly-scoring mechanism: they are 2D foundation-model baselines, 3D anomaly-synthesis engines, a preprocessing method, an acquisition method, a test-time adaptation wrapper, and an active-sensing policy. The **Status** column applies the inclusion policy of Section 1: peer-reviewed works are marked *Pub.*, and works available only as preprints are marked *Pre.* Preprints that report results on an established benchmark appear in the comparison tables of Section 13, where the Status column marks them. All preprints are treated alike: they form a separate evidence tier and none is described as establishing a state of the art.

Table S4. Complete inventory of surveyed 3D anomaly detection methods. Pub. = peer-reviewed; Pre. = preprint. Venues abbreviated. Every venue, year, and peer-review status in this table was checked against the originating publication and is frozen as verified on 18 September 2026; works appearing here as preprints may have been published since that date.

Method	Venue	Year	Status
(A1) Feature distance and memory matching			
BTF [23]	CVPRW	2023	Pub.
M3DM [22]	CVPR	2023	Pub.
Shape-Guided [46]	ICML	2023	Pub.
CPMF [17]	Pattern Recognit.	2024	Pub.
Group3AD [72]	ACM MM	2024	Pub.
PointCore [71]	arXiv	2024	Pre.
Reg3D-AD [19]	NeurIPS	2023	Pub.
LSFA [84]	ECCV	2024	Pub.
Simple3D [81]	AAAI	2026	Pub.
Reg2Inv [77]	NeurIPS	2025	Pub.

Method	Venue	Year	Status
RIF [78]	Pattern Recognit.	2026	Pub.
GLFM [79]	IEEE TASE	2025	Pub.
LCN [80]	CVPRW	2026	Pub.
SCA [83]	Sci. Rep.	2026	Pub.
AEMG [85]	IEEE TMM	2026	Pub.
APG-Net [86]	IEEE TCSVT	2026	Pub.
MISDD [47]	IEEE T-Mech	2025	Pub.
MP3D [88]	J. Manuf. Process.	2026	Pub.
MambaAD3D [89]	Array	2026	Pub.
M3DM-NR [123]	TPAMI	2025	Pub.
ISMP [135]	AAAI	2025	Pub.
MulSen-AD [87]	CVPR	2025	Pub.
G2SF [179]	ICCV	2025	Pub.
CMSA [124]	ECAI	2025	Pub.
HFMM-Net [132]	Information	2025	Pub.
CDRS [131]	Appl. Sci.	2026	Pub.
LM3M [130]	IEEE Access	2026	Pub.
(A2) Reconstruction and restoration residuals			
IMRNet [49]	CVPR	2024	Pub.
EasyNet [91]	ACM MM	2023	Pub.
MC3D-AD [18]	IJCAI	2025	Pub.
R3D-AD [36]	ECCV	2024	Pub.
3DSR/DADA [48]	WACV	2024	Pub.
PASDF [50]	ICCV	2025	Pub.
MAESTRO [101]	VISAPP	2025	Pub.
DUS-Net [102]	ACM MM	2025	Pub.
DRAIN [103]	Applied Intell.	2024	Pub.
SeDiR [96]	CVPR	2026	Pub.
CM3D-AD [92]	CVPRW	2026	Pub.
AARD [183]	CVPR	2026	Pub.
LDSD [105]	arXiv	2026	Pre.
C3D-AD [104]	arXiv	2025	Pre.
Vote3D-AD [173]	Alexandria Eng. J.	2026	Pub.
PCDiff [184]	arXiv	2026	Pre.
SPA-SDF [97]	arXiv	2026	Pre.
V2G [98]	IEEE Access	2025	Pub.
DCMUNet [99]	IEEE TIM	2025	Pub.
DAS3D [37]	ECCVW	2024	Pub.
DBRN [100]	SPIE	2024	Pub.
BridgeNet [67]	ACM MM	2025	Pub.
MAFR [127]	arXiv	2025	Pre.
DGCF [125]	Sensors	2026	Pub.
FMFR [126]	JCDE	2026	Pub.
(A3) Teacher–student and representation discrepancy			
3D-ST [51]	WACV	2023	Pub.
AST [29]	WACV	2023	Pub.
MDSS [52]	PRCV	2024	Pub.
TS-Net [177]	ESWA	2023	Pub.
HierAST [180]	ICONIP	2025	Pub.
LPFSTNet [108]	Neurocomputing	2025	Pub.
DCRDF-Net [109]	Sensors	2026	Pub.
MMRD [53]	AAAI	2024	Pub.
CFM [133]	CVPR	2024	Pub.
CMDR-IAD [172]	arXiv	2026	Pre.
TRD [107]	IEEE TMM	2026	Pub.
CMDIAD [178]	Inf. Fusion	2026	Pub.

Method	Venue	Year	Status
UCF [128]	IEEE TII	2025	Pub.
(A4) Density and likelihood estimation			
HGAD [33]	ECCV	2024	Pub.
VAE3D [34]	ICIP	2021	Pub.
MVFM-3DAD [54]	arXiv	2026	Pre.
MMFlows [55]	J. Image Graph.	2025	Pub.
2M3DF [65]	IEEE TCSVT	2025	Pub.
(A5) Registration and template deviation			
MC4AD [116]	arXiv	2025	Pre.
Template3D-AD [56]	IJCAI	2025	Pub.
3DKeyAD [57]	IEICE Trans. Inf. Syst.	2026	Pub.
SplatPose [58]	CVPRW	2024	Pub.
Looking3D [171]	CVPR	2024	Pub.
PO3AD [59]	CVPR	2025	Pub.
Marchi *et al.* [176]	Integr. Comput.-Aided Eng.	2026	Pub.
SplatPose+ [150]	ECCVW	2024	Pub.
R^3-PAD [117]	SSRN	2026	Pre.
(A6) Semantic and foundation-model transfer			
Text-Guided [175]	arXiv	2026	Pre.
UniMF [129]	IEEE TII	2026	Pub.
PointAD [20]	NeurIPS	2024	Pub.
3DzAL [60]	WACV	2025	Pub.
MVP-PCLIP [61]	IEEE TSMC	2025	Pub.
GS-CLIP [62]	CVPR	2026	Pub.
BTP [66]	CVPR	2026	Pub.
CLIP3D-AD [186]	arXiv	2024	Pre.
PLANE [181]	Expert Syst. Appl.	2026	Pub.
DMP-3DAD [182]	IEICE Trans. Inf. Syst.	2026	Pub.
CoGeoAD [185]	arXiv	2026	Pre.
ZUMA [121]	IEEE TPAMI	2026	Pub.
Where&What [122]	ACM ICMR	2026	Pub.
MCL-AD [64]	arXiv	2025	Pre.
PointAD+ [63]	arXiv	2025	Pre.
Related Techniques (not counted in the detector total)			
Fence Theorem [82]	arXiv	2025	Pre.
3D-PNAS [68]	arXiv	2025	Pre.
Synthesis4AD [69]	arXiv	2026	Pre.
AnomalyGPT [39]	AAAI	2024	Pub.
AnomalyCLIP [38]	ICLR	2024	Pub.
ReflectSL [148]	Opt. Express	2026	Pub.
ActiveInspect [147]	Sensors	2026	Pub.
TopoOT [169]	arXiv	2026	Pre.

S5. Records Excluded at Full Text

All 138 records whose full text was retrieved and assessed against inclusion criterion (iii) failed it. 110 reached this stage from the database search and 28 from citation chaining. The reason is the same for every one of them: the retrieved full text contains no term naming an established 3D anomaly detection benchmark, so the record reports no quantitative result on one.

Records whose full text could not be retrieved are a different outcome, counted separately as undecided, since failing to obtain a text is a statement about retrieval rather than about the work.

Table S5 lists all 138. Because the rule that excluded them is one rule applied uniformly, the table has no reason column: every row failed the same test. What it does carry is the identifier, so any record can be retrieved and the test re-applied to it.

Table S5. The 138 records excluded after their full text was retrieved and assessed. All failed inclusion criterion (iii): the retrieved text names no established 3D anomaly detection benchmark. *Found by* records which arm of the protocol returned the record, the database search or citation chaining.

Title	Year	Found by	Identifier
A Generic Semi-Supervised Deep Learning-Based Approach for Automated Surface Inspection	2020	chaining	10.1109/access.2020.3003588
Distance-Based Anomaly Detection for Industrial Surfaces Using Triplet Networks	2020	search	10.1109/iemcon51383.2020.9284921
Interpretable, Multidimensional, Multimodal Anomaly Detection with Negative Sampling for Detection of Device Failure	2020	search	arXiv:2007.10088
Unsupervised anomaly segmentation via deep feature reconstruction	2020	chaining	10.1016/j.neucom.2020.11.018
Unsupervised Distribution Learning for Lunar Surface Anomaly Detection	2020	search	arXiv:2001.04634
A Mura Detection Model Based on Unsupervised Adversarial Learning	2021	search	10.1109/access.2021.3069466
DRAEM – A discriminatively trained reconstruction embedding for surface anomaly detection	2021	search	arXiv:2108.07610
Efficient Non-Compression Auto-Encoder for Driving Noise-based Road Surface Anomaly Detection	2021	search	10.1002/tee.23672
Multimedia Datasets for Anomaly Detection: A Review	2021	search	10.1007/s11042-023-17425-z
Noise Reduction and Driving Event Extraction Method for Performance Improvement on Driving Noise-based Surface Anomaly Detection	2021	search	arXiv:2112.07214
Non-Compression Auto-Encoder for Detecting Road Surface Abnormality via Vehicle Driving Noise	2021	search	10.1109/ICACEH54312.2021.9768853
OneFlow: One-class flow for anomaly detection based on a minimal volume region	2021	chaining	10.1109/tpami.2021.3108223
PaDiM: A Patch Distribution Modeling Framework for Anomaly Detection and Localization	2021	chaining	10.1007/978-3-030-68799-1_35
SegmentMeIfYouCan: A Benchmark for Anomaly Segmentation	2021	chaining	arXiv:2104.14812
Semi-Supervised Surface Anomaly Detection of Composite Wind Turbine Blades From Drone Imagery	2021	search	10.5220/0010842100003124
A Survey on Unsupervised Anomaly Detection Algorithms for Industrial Images	2022	search	10.1109/ACCESS.2023.3282993
Deep Learning Based Semantic Segmentation Technique for Anomaly Detection on Metal Surfaces Using High Calibre U-Shaped Network	2022	search	10.18280/ts.390614
Diamond Abrasive Electroplated Surface Anomaly Detection using Convolutional Neural Networks for Industrial Quality Inspection	2022	search	arXiv:2212.11122
Domain-Generalized Textured Surface Anomaly Detection	2022	search	10.1109/icme52920.2022.9859637
DSR – A dual subspace re-projection network for surface anomaly detection	2022	search	10.1007/978-3-031-19821-2_31
Frequency of Interest-based Noise Attenuation Method to Improve Anomaly Detection Performance	2022	search	10.1109/bigcomp57234.2023.00048
PNI : Industrial Anomaly Detection using Position and Neighborhood Information	2022	search	10.1109/iccv51070.2023.00586
Towards intelligent monitoring system in wire arc additive manufacturing: a surface anomaly detector on a small dataset	2022	search	10.1007/s00170-022-09076-5
Zero-Shot Out-of-Distribution Detection Based on the Pre-trained Model CLIP	2022	chaining	10.1609/aaai.v36i6.20610
A Weakly Supervised Gradient Attribution Constraint for Interpretable Classification and Anomaly Detection	2023	chaining	10.1109/tmi.2023.3282789
CLF-AIAD: A Contrastive Learning Framework for Acoustic Industrial Anomaly Detection	2023	search	10.1007/978-981-99-8126-7_10
Composite score for anomaly detection in imbalanced real-world industrial dataset	2023	chaining	10.1007/s10994-023-06415-9
Deep Learning for Automatic Vision-Based Recognition of Industrial Surface Defects: A Survey	2023	chaining	10.1109/access.2023.3271748
Few Shot Part Segmentation Reveals Compositional Logic for Industrial Anomaly Detection	2023	search	10.1609/aaai.v38i8.28703
Global Calibration of Double Sensors Vision Measurement System Based on Planar Mirrors	2023	search	10.1145/3604078.3604172
MemSeg: A semi-supervised method for image surface defect detection using differences and commonalities	2023	chaining	10.1016/j.engappai.2023.105835
mixed attention auto encoder for multi-class industrial anomaly detection	2023	search	10.1109/icassp48485.2024.10446794
Myriad: Large Multimodal Model by Applying Vision Experts for Industrial Anomaly Detection	2023	search	arXiv:2310.19070
PKU-GoodsAD: A Supermarket Goods Dataset for Unsupervised Anomaly Detection and Segmentation	2023	search	10.1109/LRA.2024.3352358
Robotic Defect Inspection with Visual and Tactile Perception for Large-scale Components	2023	search	10.1109/iros55552.2023.10341590
Segment Any Anomaly without Training via Hybrid Prompt Regularization	2023	chaining	arXiv:2305.10724

Title	Year	Found by	Identifier
TransFusion – A Transparency-Based Diffusion Model for Anomaly Detection	2023	search	10.1007/978-3-031-72761-0_6
U-Flow: A U-shaped Normalizing Flow for Anomaly Detection with Unsupervised Threshold	2023	chaining	10.21203/rs.3.rs-3367286/v1
3D-CSAD: Untrained 3D Anomaly Detection for Complex Manufacturing Surfaces	2024	search	arXiv:2404.07748
A hierarchical geometry-to-semantic fusion GNN framework for earth surface anomalies detection	2024	search	10.11834/jrs.20243301
AnomalyDINO: Boosting Patch-based Few-shot Anomaly Detection with DINOv2	2024	search	10.1109/wacv61041.2025.00136
Cosine similarity knowledge distillation for surface anomaly detection	2024	search	10.1038/s41598-024-58409-9
CSE: Surface Anomaly Detection with Contrastively Selected Embedding	2024	search	10.5220/0012546700003660
Effectiveness Assessment of Recent Large Vision-Language Models	2024	search	10.1007/s44267-024-00050-1
FiLo: Zero-Shot Anomaly Detection by Fine-Grained Description and High-Quality Localization	2024	search	10.1145/3664647.3680685
Global-Regularized Neighborhood Regression for Efficient Zero-Shot Texture Anomaly Detection	2024	search	10.1109/tsmc.2025.3595539
Marble Surface Anomaly Detection Using Autoencoder Architecture	2024	search	10.21776/jeeccis.v18i1.1685
Multi-Scale Rail Surface Anomaly Detection Based on Weighted Multivariate Gaussian Distribution	2024	search	10.1587/transinf.2024edp7049
Multimodal Anomaly Detection based on Deep Auto-Encoder for Object Slip Perception of Mobile Manipulation Robots	2024	search	10.1109/ICRA48506.2021.9561586
Multimodal anomaly detection for high-speed train control system based on attention mechanism	2024	search	10.21203/rs.3.rs-3974253/v1
Multiscale Prototype Fusion Network for Industrial Product Surface Anomaly Detection and Localization	2024	search	10.1109/jsen.2024.3450749
On The Relationship between Visual Anomaly-free and Anomalous Representations	2024	search	10.1007/978-3-031-92805-5_16
Progressive Boundary Guided Anomaly Synthesis for Industrial Anomaly Detection	2024	search	10.1109/TCSVT.2024.3479887
Steel Surface Defect Detection Using Improved Deep Learning Algorithm: ECA-SimSPPF-SIoU-Yolov5	2024	chaining	10.1109/access.2024.3371584
Towards VLM-based Hybrid Explainable Prompt Enhancement for Zero-Shot Industrial Anomaly Detection	2024	search	10.24963/ijcai.2024/80
Transfer Learning Based Hybrid Quantum Neural Network Model for Surface Anomaly Detection	2024	search	10.1109/isvlsi61997.2024.00121
A computer vision system for recognition and defect detection for reusable containers	2025	search	10.1007/s00138-024-01636-x
A Survey on Industrial Anomalies Synthesis	2025	search	arXiv:2502.16412
A systematic survey: role of deep learning-based image anomaly detection in industrial inspection contexts	2025	search	10.3389/frobt.2025.1554196
AD-DINOv3: Enhancing DINOv3 for Zero-Shot Anomaly Detection with Anomaly-Aware Calibration	2025	search	10.1109/icassp55912.2026.11464463
ADSeeker: A Knowledge-Grounded Reasoning Framework for Industry Anomaly Detection and Reasoning	2025	search	arXiv:2508.03088
Anomaly detection in medical via multimodal foundation models	2025	chaining	10.3389/fbioe.2025.1644697
Anomaly Detection of TP4056 Charging Module Based on Improved SimpleNet	2025	search	10.62517/jbdc.202501418
Bayesian Prompt Flow Learning for Zero-Shot Anomaly Detection	2025	search	10.1109/cvpr52734.2025.02830
Can Multimodal Large Language Models be Guided to Improve Industrial Anomaly Detection?	2025	search	10.1115/detc2025-168875
Computer Vision-Based Anomaly Detection in Industrial Components	2025	search	10.71097/ijsat.v16.i2.6154
CXR-AD: Component X-ray Image Dataset for Industrial Anomaly Detection	2025	search	arXiv:2505.03412
Deep learning-based anomaly detection for precision field crop protection	2025	chaining	10.3389/fpls.2025.1576756
Detect, Classify, Act: Categorizing Industrial Anomalies with Multi-Modal Large Language Models	2025	search	10.1109/cvprw67362.2025.00390
DZAD: Diffusion-based Zero-shot Anomaly Detection	2025	search	10.1609/aaai.v39i10.33099
Embodied Hazard Mitigation using Vision-Language Models for Autonomous Mobile Robots	2025	search	arXiv:2509.06768
Explainable Anomaly Detection in Multimodal Telemetry of Banking Microservices	2025	search	10.70315/uloap.ulbec.2025.0204015
FiLo++: Zero-/Few-Shot Anomaly Detection by Fused Fine-Grained Descriptions and Deformable Localization	2025	search	10.1109/tcsvt.2026.3671805
Foundation models and Transformers for anomaly detection: A survey	2025	chaining	10.1016/j.inffus.2025.103517
Frequency-Enhanced Dual-Layer Anomaly Synthesis for Real-Time Industrial Surface Inspection	2025	search	10.21203/rs.3.rs-7240661/v1
Hyperbolic geometry enhanced feature filtering network for industrial anomaly detection	2025	search	10.1038/s41598-025-07550-0

Title	Year	Found by	Identifier
IAD-GPT: Advancing Visual Knowledge in Multimodal Large Language Model for Industrial Anomaly Detection	2025	search	10.1109/tim.2025.3635334
LAD-Reasoner: Tiny Multimodal Models are Good Reasoners for Logical Anomaly Detection	2025	search	arXiv:2504.12749
Large language models for PHM: a review of optimization techniques and applications	2025	chaining	10.1007/s43684-025-00100-5
Light-MLLMAD: A Lightweight Multimodal Large Language Model for One-Shot Industrial Visual Anomaly Detection	2025	search	10.21203/rs.3.rs-7853870/v1
LR-IAD:Mask-Free Industrial Anomaly Detection with Logical Reasoning	2025	search	10.1109/icdm65498.2025.00097
MACHINE LEARNING-DRIVEN ANOMALY DETECTION IN INFORMATION SYSTEM VERIFICATION AND VALIDATION	2025	search	10.36074/grail-of-science.18.04.2025.077
Metal loss defect detection and depth estimation using multispectral image analysis of cooling excited steel specimen with corrosion	2025	chaining	10.1038/s41598-025-88528-w
MIAC-Flow: Multi-level Interactive Affine Coupling Flow withFrequency-Spatial Cooperative Attention for Industrial Anomaly Detection	2025	search	10.21203/rs.3.rs-7958772/v1
Multimodal anomaly detection framework in intelligent financial audit: based on LightGBM-SVM hybrid model and dynamic feature enhancement	2025	search	10.1145/3746469.3746597
Multimodal Anomaly Detection with a Mixture-of-Experts	2025	search	10.1109/iros60139.2025.11245878
Multimodal zero-shot anomaly detection using dual-experts for electrical power equipment inspection images	2025	search	10.11834/jig.240246
No label left behind: a unified surface defect detection model for all supervision regimes	2025	chaining	10.1007/s10845-025-02680-8
OmniAD: Detect and Understand Industrial Anomaly via Multimodal Reasoning	2025	search	arXiv:2505.22039
On the Problem of Consistent Anomalies in Zero-Shot Anomaly Detection	2025	search	arXiv:2512.02520
Optimized edge weighting in graph neural networks for server performance anomaly detection	2025	chaining	10.1007/s40747-025-02069-3
PA-CLIP: Enhancing Zero-Shot Anomaly Detection through Pseudo-Anomaly Awareness	2025	search	arXiv:2503.01292
PB-IAD: Utilizing multimodal foundation models for semantic industrial anomaly detection in dynamic manufacturing environments	2025	search	arXiv:2508.14504
Prescriptive Agents based on RAG for Automated Maintenance (PARAM)	2025	search	10.1109/aimlsystems67835.2025.11330545
Quantitative estimation method for complex part surface defects based on multimodal information fusion	2025	chaining	10.1007/s40747-025-01874-0
Research on the key techniques of 3D defect detection on aeroengine blade surface utilizing robotic vision	2025	search	10.33737/gpps25-tc-210
RobustA: Robust Anomaly Detection in Multimodal Data	2025	search	arXiv:2511.07276
Robustness of unsupervised methods for image surface-anomaly detection	2025	search	10.1007/s10044-025-01477-y
RSTD-YOLOv7: a steel surface defect detection based on improved YOLOv7	2025	chaining	10.1038/s41598-025-04811-w
SAFNet-IoT: Secure Adaptive and Cloud supported Federated Network for IoT-Based Industrial Anomaly Detection	2025	search	10.21203/rs.3.rs-6376809/v1
SAGE: A Visual Language Model for Anomaly Detection via Fact Enhancement and Entropy-aware Alignment	2025	search	10.1145/3746027.3755725
SALAD – Semantics-Aware Logical Anomaly Detection	2025	search	arXiv:2509.02101
SuperAD: A Training-free Anomaly Classification and Segmentation Method for CVPR 2025 VAND 3.0 Workshop Challenge Track 1: Adapt & Detect	2025	search	arXiv:2505.19750
Surface Anomaly Detection on Shock Absorber Connecting Rods Using an Enhanced PatchCore Algorithm	2025	search	10.1587/transinf.2025edl8047
Unsupervised Anomaly Detection on Implicit Shape Representations for Sarcopenia Detection	2025	chaining	10.1109/isbi60581.2025.10980714
Unsupervised Wood Surface Anomaly Detection via Enhanced GAN with Residual Dense and Attention Modules	2025	search	10.65286/icic.v21i4.61165
YOLO-MSD: a robust industrial surface defect detection model via multi-scale feature fusion	2025	chaining	10.1007/s10489-025-06739-0
3D-DefectBench: A Controlled Factorial Study of Vision-Language Model Evaluation Pipelines for Fine-Grained 3D Generation Defects	2026	search	arXiv:2607.10826
3D-FPFH-Int: a hybrid geometric–radiometric descriptor for structural surface anomaly detection in tropical heritage	2026	search	10.1038/s41598-026-59034-4
A Defect Dataset for Electrode Coating Manufacturing	2026	chaining	10.1038/s41597-025-06419-1
A Dual-Stream Physics-Augmented Unsupervised Architecture for Runtime Embedded Vehicle Health Monitoring	2026	search	arXiv:2602.10432
A Structured Benchmark for Text-Guided Anomaly Detection: When Language Stops Conditioning the Decision	2026	search	arXiv:2606.01992
A three-dimensional vision-based method for weak-texture defect detection and precise measurement in electrical automation manufacturing	2026	search	10.1186/s44147-026-00950-7

Title	Year	Found by	Identifier
A unified vision-language model for cross-product defect detection in glove manufacturing	2026	chaining	10.1371/journal.pone.0339867
AnomalyAgent: Training-Free Agentic Models for Zero-/Few-Shot Anomaly Detection	2026	search	arXiv:2605.30140
Beyond local receptive fields: vision transformers for real-time surface defect detection in FDM	2026	search	10.1007/s00170-026-17846-8
CLIP-MDC: CLIP encoder based multimodal defect classification with synthetic anomaly generation for real-time surface defect detection	2026	chaining	10.1007/s10845-025-02773-4
Distribution-free false-alarm calibration and chance-corrected spatial evaluation for industrial anomaly detection	2026	search	arXiv:2608.15090
Enhanced structural anomaly detection through improved image inpainting and feature-level discrimination	2026	search	10.1038/s41598-026-60212-7
Generalist Graph Anomaly Detection via Prototype-Based Distillation	2026	search	arXiv:2605.26857
Generalizing CLIP prompts for zero-shot anomaly detection	2026	search	10.1016/j.patcog.2026.113406
Global Logic and Local Search: Dual-Stream Multimodal In-Context Learning for Verifiable Industrial Anomaly Detection	2026	search	arXiv:2607.03817
GRD-Net: Generative-Reconstructive-Discriminative Anomaly Detection with Region of Interest Attention Module	2026	search	10.1155/2023/7773481
IstGPT: LLM-based Anomaly Detection for Spatial-Temporal Graph in Industrial Systems	2026	search	arXiv:2606.01691
Mahalanobis PatchCore: Covariance-Aware and Streaming-Compatible Industrial Anomaly Detection	2026	search	arXiv:2605.27748
MemeChain: A Multimodal Cross-Chain Dataset for Meme Coin Forensics and Risk Analysis	2026	search	arXiv:2601.22185
Multi-view Patch Inference with Frozen DINOv3 for Industrial Anomaly Detection	2026	search	10.21203/rs.3.rs-10224105/v1
Multimodal Anomaly Detection for Human-Robot Interaction	2026	search	arXiv:2604.09326
Myriad: a large multimodal model applying vision experts for industrial anomaly detection	2026	search	10.1007/s11432-025-4976-0
OSAGEN: Object-Aware Mask Priors and Multistage Decoupled Diffusion for Industrial Anomaly Generation	2026	search	arXiv:2607.29533
ReLU Networks for Exact Generation of Similar Graphs	2026	search	arXiv:2604.05929
Resource-Efficient Adaptation of Large Vision–Language Models for Multimodal Defect Inspection in LNG Tank Manufacturing	2026	chaining	10.1007/s00170-026-17818-y
Self-Supervised Learning Framework for Multimodal Environmental Pollution Assessment	2026	search	10.64137/31079377/ijmsd-v2i2p105
SubspaceAD: Training-Free Few-Shot Anomaly Detection via Subspace Modeling	2026	search	arXiv:2602.23013
ThermalCLIP: Hardware-Agnostic Industrial Anomaly Detection via Physics-Informed Thermal Proxies from RGB	2026	search	10.21203/rs.3.rs-9385007/v1
Towards Explainable Industrial Anomaly Detection via Knowledge-Guided Latent Reasoning	2026	search	arXiv:2602.09850
UltraVR: A Diagnostic Ultra-Resolution Image-VQA Benchmark for Evidence-Grounded Reasoning	2026	search	arXiv:2606.05576
VTFusion: A Vision-Text Multimodal Fusion Network for Few-Shot Anomaly Detection	2026	search	10.1109/tcyb.2026.3651630

S6. Protocol Measurements

Four properties of the search-and-selection protocol of Section 1 were measured against the corpus rather than assumed: how much of it the documented search recovers, what citation chaining adds, how sensitive the screening rules are, and what the abstract-level proxy costs. Each is reported here in full because each is an audit of the instrument rather than a finding about the field.

S6.1. Recovery and the Residual

The documented search queried both sources in one run on 21 August 2026. The executed strings differ by source and are given verbatim so the identification stage can be repeated rather than approximated: arXiv was queried as `all:"<phrase>"` and OpenAlex as `title_and_abstract.search:"<phrase>"` with `from_publication_date=2020-01-01`, and Table S1 supplies the phrases. No language restriction and no document-type restriction were applied. We report that as an absence rather than leave it to be inferred: the search was not limited to English, and preprints, conference papers and journal articles were all retained, with peer-review status recorded per work instead of used as a filter. Beyond citation chaining, no author searches, no repository or code-hosting searches, and no hand-searching of specific venues were performed. That date freezes

the identification search. The stages that run on what it returned, citation chaining, full-text eligibility, and the widened-query test of Section S2, were executed on 23 August 2026. Re-issuing a query after the freeze also returns records that did not exist at it, so the widened test is scored on which works its own queries reach rather than on a change in the total; it added 0 records to the screening pool. Table S2 lists all 23 queries of that test beside the 16 reported ones.

The corpus this survey had already assembled by expert reading serves as the test set. *Recovery*: the documented search retrieves 93 of the 102 works in our inventory (91%). That figure is not uniformly held out and we do not present it as one: eight of the 16 queries were added after an earlier recovery test, against an inventory of 70 works, so they were fitted to most of what they are scored on. Restricting to the 32 works added after that fit, which no query in the set could have been shaped by, recovery is 26 (81%). We take the lower figure as the honest one.

The 9 works the search does not reach fall into two kinds. Some resolve to no record in the index the chaining runs on, so no path in this protocol reaches them. The rest are indexed, but neither their titles nor their abstracts contain a phrase that any query in the released set matches; the limit reached there is set by phrase matching, not by what the databases hold. Citation chaining reaches exactly this second kind. Over 102 seeds it added 995 records the database search had not returned, of which 334 passed screening, and it recovered 6 of the 9; Shape-Guided, LCN, AARD remain. We report that measurement rather than let the stage rest on its rationale, because it locates the residual precisely. Whether a wider query vocabulary would close the second kind is a question we tested rather than argued, and Section S2 reports the result.

S6.2. Single-Reviewer Screening

Screening was carried out by a single reviewer. There was therefore no second screener, no inter-rater agreement statistic, and no disagreement-resolution procedure; dual independent screening is the standard and this survey does not meet it. What partly compensates is that the decisions are not discretionary: the rules are code, applied identically to every record, and each exclusion is written to the released log with the rule that caused it. Full-text eligibility was assessed by the same reviewer; it too is rule-based, testing the retrieved text for the name of an established benchmark, and Section S5 reports how many records it excluded.

Screening on title and abstract excluded 734 records, each attributed to one of three recorded rules: 83 for naming an out-of-scope domain, 13 for carrying no anomaly, defect, or inspection term, and 638 for carrying no 3D or depth-bearing modality term. Records without an abstract were carried forward rather than excluded, since absence of an abstract is not evidence of irrelevance.

S6.3. Screening Sensitivity and the Abstract-Level Proxy

Screening sensitivity: applied to the retrieved records for works already known to be in scope, the screening rules retain 86 of 87 (99%). The single false negative is HGAD [33], which names no 3D modality in either its title or its abstract. We report it rather than widen a rule to accommodate it, because the miss is informative: papers in this field do not reliably declare their modality where an automated screen can see it.

Eligibility criterion (iii) requires reading the full text, since a paper may report a benchmark in a table without naming it in its abstract. An abstract-level proxy orders that reading queue: 121 records name a known benchmark in title or abstract, and 871 do not. The proxy is not a substitute for the assessment, and we do not treat it as one: only 66 of the 87 known in-scope works pass it, so 24% of the papers we know qualify would be lost by a filter that stopped there.

The categorical fields of the inventory were extracted from the source and checked against it, but not exhaustively: the released inventory carries one source-location column, filled where a value was read from a table or page and blank otherwise. We make that narrower claim rather than the stronger one because the two kinds of extraction did not prove equally reliable.

S7. Audit of the Collated Results

Two properties of the comparison tables of Section 13 are reported here rather than in the body: which reported values the protocol-compatibility rule held back, and how far each work in the inventory can be followed back to its source.

S7.1. Values Held Out on Protocol Grounds

The ledger behind the comparison tables carries a protocol-compatibility flag on every result, and the flag is enforced: a value marked incompatible cannot be placed in a comparison table. Enforcing a rule silently is only half of stating one, so Table S6 lists what was held back and why.

None of these values is disputed. Each was read from its source and verified like every other number in this survey. Two kinds recur. A value may be an aggregate over a subset of the benchmark, as with the seven-of-ten-category means, in which case it is not the quantity the table's column reports. Or it may come from a different evaluation regime, as with a unified multi-category model or a one-shot result, in which case comparing it against per-category or full-data entries would reward the regime rather than the method.

Table S6. Reported values held out of Tables 10–12 on protocol grounds, with the reason recorded when each was checked against its source. These are not disputed numbers and no claim is made that they are wrong; each was produced under a protocol the corresponding table does not compare, so placing it there would assert a comparison the value does not support. 6 values across 4 methods.

Method	Benchmark	Metric	Value	Why it is not comparable
MC4AD	MVTec 3D-AD	I-AUROC	95.4	source Table 16 reports O-AUROC on only-3D MVTec 3D-AD; the same condition under which MVFM-3DAD is excluded from this table
MP3D	MVTec 3D-AD	I-AUROC	95.0	mean over seven of ten categories; dowel, foam and rope absent
MP3D	MVTec 3D-AD	P-AUROC	98.5	mean over the same seven categories
SeDiR	Anomaly-ShapeNet	O-AUROC	93.3	93.3% O-AUROC in a unified multi-category setting; the per-category tables compare methods trained one model per category, so this is cited in prose rather than tabulated. The exclusion was recorded here before the comparable flag existed and was overridden on 2026-08-23 by a guard that could only read flags
UniMF	MVTec 3D-AD	AUPRO	90.4	1-shot; the source gives the series as 76.87, 78.16 and 79.44 I-AUROC for 1, 2 and 4 shots. The table's other rows are full-data, and the whole result is one configuration, so every metric of it is excluded together
UniMF	MVTec 3D-AD	I-AUROC	76.9	1-shot; the source gives the series as 76.87, 78.16 and 79.44 I-AUROC for 1, 2 and 4 shots. The table's other rows are full-data, and the whole result is one configuration, so every metric of it is excluded together

S7.2. Evidence Strength

The risk-of-bias instruments used for clinical trials are built around randomization, blinding and outcome reporting, none of which transfers to algorithm papers. What can be assessed is how far a reported result can be followed back, so that is what Table S7 measures, on three axes complete for every work in the inventory.

Peer review records whether the work appeared in a reviewed venue at the date its status was checked. *Traceability* records whether at least one number we report for it was located in the originating publication and checked against it, with the location released alongside the inventory. *Breadth* counts the established benchmarks the work reports on, since a method evaluated on one benchmark supports a narrower claim than one evaluated on several.

Of the 102 works, 73 appeared in a reviewed venue and 57 have at least one source-checked result. 47 satisfy both and 19 satisfy neither, the latter being preprints whose reported numbers we could not place in the source. 13 report on two or more benchmarks.

None of these axes judges whether a method works, whether its protocol is sound, or whether its result would survive re-running. A preprint with a traceable number scores above a peer-reviewed paper whose numbers we could not place, which is a statement about our ability to audit the two rather than about their quality. Code availability is deliberately absent: it is verified for 30 of the 102 works (Section 15), and an axis populated for under a third of the corpus would rank works by how much attention they happened to receive.

Table S7. Evidence strength across the 102 works in the inventory. *Peer-reviewed* is the venue's status at the date recorded per work. *Traceable* means at least one reported number was located in the originating publication and checked against it, with the location released. *Benchmarks* counts the established benchmarks a work reports on. None of the three judges whether a method is good; they measure how far its evidence can be followed.

Axis	Works	Share
Peer-reviewed venue	73	72%
At least one traceable, source-checked result	57	56%
Both of the above	47	46%
Neither	19	19%
Reports on two or more benchmarks	13	13%
Reports on one benchmark	44	43%
No collated benchmark result	45	44%

S8. Source-Location Ledger

Table S8 lists every benchmark value this survey reports, with the place in the originating publication where it was read. It is the per-entry form of the traceability axis summarised in Table S7, and it is what makes the collated tables of Section 13 checkable one number at a time.

The locations are not uniform, and the table does not make them look uniform. 80 of the 143 rows name a table, figure, section or page; the rest record that the value was found in the source's running text without an anchor to it. Every row was checked against the source. The difference is in how precisely the check was written down, not in whether it happened.

Table S8. Every benchmark value reported in this survey and where it was read in the originating publication. Values are grouped benchmark, in the order the comparison tables of Section 13 present them. 143 values across 59 methods.

Method	Metric	Value	Where it was read
MVTec 3D-AD			
2M3DF [65]	AUPRO	97.5	Table I mean AUPRO 0.975
2M3DF [65]	I-AUROC	96.6	Abstract and results: mean I-AUROC
3D-ST [51]	AUPRO	83.3	reported in text: 0.833 mean AUPRO
3DSR/DADA [48]	AUPRO	97.2	Table 2, 3D+RGB mean AUPRO 97.2
3DSR/DADA [48]	I-AUROC	97.8	reported in text: 97.8 mean I-AUROC, rank 1 on 7/10 categories (3D setting)
AEMG [85]	AUPRO	94.4	Sec. V
AEMG [85]	I-AUROC	95.6	Sec. V
AST [29]	I-AUROC	93.7	reported in text: 93.7 mean I-AUROC
BTF [23]	AUPRO	96.4	Table 1 PRO 0.964
BTF [23]	I-AUROC	86.5	Table 1 I-ROC for RGB+PC(FPFH)
BTF [23]	P-AUROC	99.3	reported in text: pixel-wise ROCAUC 99.3%
CDRS [131]	AUPRO	92.6	released-code full setting, against baseline 91.5
CDRS [131]	I-AUROC	80.0	released-code full setting
CDRS [131]	P-AUROC	98.0	released-code full setting, against baseline 97.5
CFM [133]	AUPRO	97.1	Table 1 Mean: 0.971 AUPRO@30% (Ours, full model)
CFM [133]	I-AUROC	95.4	Table 1 Mean: 0.954 (Ours, full model)
CMSA [124]	AUPRO	97.6	Table 1 Mean, AUPRO@30%
CMSA [124]	AUPRO@1	46.3	Table 3, the stricter FPR 0.01 integration limit
CMSA [124]	I-AUROC	98.4	Table 1 Mean
CPMF [17]	AUPRO	92.9	Table: 0.9290 (abstract text: 92.93%)
CPMF [17]	I-AUROC	95.2	Table: 0.9520 (abstract text: 95.15%)
DAS3D [37]	AUPRO	98.2	Table 2, RGB+3D mean AUPRO 0.982
DAS3D [37]	I-AUROC	98.2	abstract: SOTA I-AUROC 0.982 on MVTec 3D-AD
DCMUNet [99]	AUPRO	96.9	Table XI P-AUPRO average
DCMUNet [99]	I-AUROC	94.1	Table XI multimodal average across 10 categories
DCRDF-Net [109]	I-AUROC	97.1	Sec. 4.2 class-wise mean I-AUROC
DCRDF-Net [109]	PRO	98.8	Sec. 4.2 class-wise mean PRO
DGCF [125]	AUPRO	97.6	Sec. 4 results
DGCF [125]	I-AUROC	97.3	Sec. 4 results
EasyNet [91]	AUPRO	82.1	Table 7, RGB+Depth mean AUPRO 0.821
EasyNet [91]	FPS	94.55	measured on Tesla V100
EasyNet [91]	I-AUROC	92.6	abstract: 92.6% AUROC, 94.55 FPS on Tesla V100

Method	Metric	Value	Where it was read
FMFR [126]	AUPRO	97.9	prose: mean AUPRO@30% 97.9%
FMFR [126]	I-AUROC	98.0	prose: mean I-AUROC 98.0%
HFMM-Net [132]	AUPRO	98.2	prose: AUPRO@30% mean 0.982 against TRD 0.979
HFMM-Net [132]	I-AUROC	95.6	Table 1 Mean
HFMM-Net [132]	P-AUROC	99.3	prose: 0.993 against TRD 0.992
LM3M [130]	AUPRO	95.6	compressed model
LM3M [130]	I-AUROC	92.9	compressed model
LM3M [130]	P-AUROC	98.5	compressed model
LPFSTNet [108]	I-AUROC	94.7	Sec. 4 highest I-AUROC of the compared variants
LPFSTNet [108]	P-AUROC	99.2	Sec. 4 results table
LSFA [84]	AUPRO	96.8	Table 2, RGB+3D mean AUPRO 0.968
LSFA [84]	I-AUROC	97.1	abstract + prose: 97.1% I-AUROC (+3.4% over prior SOTA)
M3DM [22]	AUPRO	96.4	reported in text: 0.964 mean AUPRO
M3DM [22]	I-AUROC	94.5	reported in text: 0.945 mean I-AUROC (RGB+3D)
M3DM-NR [123]	AUPRO	96.5	Table 2, RGB+3D mean AUPRO 96.5
M3DM-NR [123]	I-AUROC	94.5	Table 1 (clean setting, RGB+3D block): Mean 94.5
MAFR [127]	AUPRO	97.0	Table III Full column, AUPRO@30% 0.970
MAFR [127]	I-AUROC	97.2	abstract + prose: mean I-AUROC 0.972 on MVTec 3D-AD
MC4AD [116]	I-AUROC	95.4	source Table 16 reports O-AUROC on only-3D MVTec 3D-AD
MCL-AD [64]	AP	96.9	Table I image-level (AUROC, AP) Mean column
MCL-AD [64]	AUPRO	92.2	Table I pixel-level (AUROC, PRO) Mean column
MCL-AD [64]	I-AUROC	89.0	Table I image-level (AUROC, AP) Mean column
MCL-AD [64]	P-AUROC	97.7	Table I pixel-level (AUROC, PRO) Mean column
MISDD [47]	AUPRO	92.68	Table I, 3D modality missing at eta=30%
MISDD [47]	I-AUROC	81.97	Table I, 3D modality missing at eta=30%
MISDD [47]	I-AUROC	77.71	Table I, both modalities missing at eta=30%
MISDD [47]	P-AUROC	98.16	Table I, 3D modality missing at eta=30%
MMFlows [55]	AUPRO	96.1	English abstract: segmentation AUPRO
MMFlows [55]	I-AUROC	93.3	English abstract, Result section
MMFlows [55]	P-AUROC	98.8	English abstract: segmentation AUCROC
MMRD [53]	AUPRO	97.6	Table 1(a) RGB+3D row Mean
MMRD [53]	I-AUROC	95.0	Table 1(a) RGB+3D row Mean
MMRD [53]	I-AUROC	89.8	Table 1(a) RGB-only row Mean
MP3D [88]	I-AUROC	95.0	mean over seven of ten categories
MP3D [88]	P-AUROC	98.5	mean over the same seven categories
MVFM-3DAD [54]	O-AUROC	95.9	Table 2 Mean
MVFM-3DAD [54]	P-AUROC	95.3	Table 2 Mean
PointAD [20]	AUPRO	84.4	Table 1, MVTec3D-AD point-only (L.) AUPRO 84.4, the row matching the 82.0 I-AUROC already collated
PointAD [20]	I-AUROC	82.0	Table: 82.0 I-AUROC on MVTec3D-AD
PointAD+ [63]	AUPRO	88.7	Table 1 one-vs-rest, point-only ZS 3D
PointAD+ [63]	AUPRO	93.1	multimodal 3D table (ML. level)
PointAD+ [63]	I-AUROC	82.5	Table 1 one-vs-rest, point-only ZS 3D
PointAD+ [63]	I-AUROC	87.7	multimodal 3D table (MG. level)
PointAD+ [63]	P-AUROC	96.9	Table 1 one-vs-rest, point-only ZS 3D
PointAD+ [63]	P-AUROC	98.2	multimodal 3D table (ML. level)
Shape-Guided [46]	AUPRO	97.6	ICML'23 PDF (PMLR v202): per-category AUPRO row averages to 0.976
Shape-Guided [46]	I-AUROC	94.7	ICML'23 PDF (PMLR v202): per-category I-AUROC row averages to 0.947
UCF [128]	AUPRO	97.5	abstract, P-AUPRO at integration limit 0.3 = 0.975
UCF [128]	I-AUROC	96.3	Table I and the contribution list
UniMF [129]	AUPRO	90.4	1-shot
UniMF [129]	I-AUROC	76.9	1-shot
V2G [98]	AUPRO	97.8	Table 2 average AUPRO(0.3) 0.978
V2G [98]	I-AUROC	96.8	abstract and results: mean image-level AUROC 0.968
ZUMA [121]	I-AUROC	84.0	Table I multimodal setting
ZUMA [121]	P-AUROC	97.6	Table I multimodal setting
ZUMA [121]	PRO	92.5	Table I multimodal setting
Real3D-AD			

Method	Metric	Value	Where it was read
3DKeyAD [57]	O-AUROC	80.1	prose: best average O-AUROC 0.801 (Raw+FPFH+ISS+FS variant)
3DKeyAD [57]	P-AUROC	86.1	prose: highest mean P-AUROC 0.861
DUS-Net [102]	O-AUROC	79.9	abstract: Real3D-AD O-AUROC 79.9
DUS-Net [102]	P-AUROC	71.2	abstract: Real3D-AD P-AUROC 71.2
Group3AD [72]	O-AUROC	75.4	Table: 0.754
IMRNet [49]	O-AUROC	72.5	abstract + prose: 72.5% I-AUC on Real3D-AD
ISMP [135]	O-AUROC	76.7	Table 1 mean 0.767 (paper prose states 0.757
ISMP [135]	P-AUROC	83.6	prose: P-AUROC 0.836
LDSD [105]	O-AUROC	85.9	p.5 experiments: "average O-AUROC of 0.921 and an average P-AUROC of 0.924" (Anomaly-ShapeNet), "average O-AUROC and P-AUROC performance of 0.859 and 0.852" (Real3D-AD)
LDSD [105]	P-AUROC	85.2	p.5 experiments: "average O-AUROC of 0.921 and an average P-AUROC of 0.924" (Anomaly-ShapeNet), "average O-AUROC and P-AUROC performance of 0.859 and 0.852" (Real3D-AD)
MC3D-AD [18]	O-AUROC	78.2	prose: AUROC 0.782 at object level
MC3D-AD [18]	P-AUROC	76.8	prose: 0.768 at pixel level
MVFM-3DAD [54]	O-AUROC	90.8	Table 1 Mean column
MVFM-3DAD [54]	P-AUROC	96.0	Table 1 Mean column
MambaAD3D [89]	O-AUROC	78.4	abstract: 86.8% and 78.4% object-level AUROC on Anomaly-ShapeNet and Real3D-AD
MambaAD3D [89]	P-AUROC	76.6	abstract
PASDF [50]	O-AUROC	80.2	abstract: 80.2% object-level AUROC on Real3D-AD
PO3AD [59]	O-AUROC	76.5	Table 3 Average: 76.5 (runner-up 75.1)
PointAD+ [63]	O-AUROC	83.5	Table 1 one-vs-rest
PointAD+ [63]	P-AUROC	76.0	Table 1 one-vs-rest, point-only ZS 3D
PointCore [71]	O-AUROC	82.9	Table III: O-AUROC 0.829
PointCore [71]	P-AUROC	73.1	Table III: P-AUROC 0.731
R3D-AD [36]	O-AUROC	73.4	abstract: 73.4% image-level AUROC on Real3D-AD
ReflectSL [148]	mIoU	81.18	segmentation metrics, not AUROC
Reg3D-AD [19]	O-AUROC	70.4	Real3D-AD paper table: 0.704
Reg3D-AD [19]	P-AUROC	70.5	Real3D-AD paper table: 0.705
Template3D-AD [56]	O-AUROC	84.4	Table 1: 84.4% mean I-AUROC (object level) on Real3D-AD
Anomaly-ShapeNet			
DUS-Net [102]	O-AUROC	79.5	abstract: Anomaly-ShapeNet O-AUROC 79.5
DUS-Net [102]	P-AUROC	84.7	abstract: Anomaly-ShapeNet P-AUROC 84.7
IMRNet [49]	O-AUROC	66.1	abstract + prose + ar5iv table Mean 0.661
LDSD [105]	O-AUROC	92.1	p.5 experiments: "average O-AUROC of 0.921 and an average P-AUROC of 0.924" (Anomaly-ShapeNet), "average O-AUROC and P-AUROC performance of 0.859 and 0.852" (Real3D-AD)
LDSD [105]	P-AUROC	92.4	p.5 experiments: "average O-AUROC of 0.921 and an average P-AUROC of 0.924" (Anomaly-ShapeNet), "average O-AUROC and P-AUROC performance of 0.859 and 0.852" (Real3D-AD)
MC3D-AD [18]	O-AUROC	84.2	prose: object-level AUROC 0.842, +9.3% over second-best single-category method
MambaAD3D [89]	O-AUROC	86.8	results section
MambaAD3D [89]	P-AUROC	91.2	abstract
PASDF [50]	O-AUROC	90.0	abstract: 90.0% object-level AUROC on Anomaly-ShapeNet
R3D-AD [36]	O-AUROC	74.9	abstract + Table 2: 74.9% image-level AUROC on Anomaly-ShapeNet
SeDiR [96]	O-AUROC	93.3	93.3% O-AUROC in a unified multi-category setting
Template3D-AD [56]	O-AUROC	86.5	Table 2: 86.5% mean I-AUROC (object level)
ArtiAD			
SPA-SDF [97]	O-AUROC	88.43	Table 3, seen articulation split, mean over 39 categories
SPA-SDF [97]	O-AUROC	87.40	Table 3, unseen articulation split
SPA-SDF [97]	P-AUROC	86.51	Table 3, seen split, point-level AUROC
SPA-SDF [97]	P-AUROC	84.10	Table 3, unseen split, point-level AUROC

Method	Metric	Value	Where it was read
EyeCandies			
CMSA [124]	I-AUROC	90.3	Table 4
HFMM-Net [132]	I-AUROC	88.7	prose: 0.887, second to CFM 0.891
MAFR [127]	I-AUROC	90.1	abstract + prose: mean I-AUROC 0.901 on Eyecandies
PointAD+ [63]	I-AUROC	69.9	Table 1 one-vs-rest, point-only ZS 3D
IEC3D-IMAD			
M3DM [156]	O-AUROC	94.0	Table II Mean, M3DM with Point-MAE features
PatchCore [156]	O-AUROC	52.0	Table II Mean, PatchCore with FPFH
MulSen-AD			
APG-Net [86]	O-AUROC	97.4	Abstract and results
PAD			
R^3-PAD [117]	I-AUROC	97.9	Table 1 mean image-level AUROC over 5 runs on MAD-Sim (20 LEGO categories)
SplatPose+ [150]	AUPRO	96.9	Sec. 4
SplatPose+ [150]	I-AUROC	96.4	Sec. 4
Where&What [122]	AUPRO	97.1	Table on LEGO/MAD dataset
Where&What [122]	P-AUROC	99.7	Table on LEGO/MAD dataset

S9. Code Availability, Per Work

Section 15 reports that of the 30 candidate repository links extracted from the surveyed papers and opened, 27 were the authors' own and 4 of those announce code that is not there. Table S9 gives the verdict per link, so the claim can be rechecked against the repositories rather than taken on the count.

Two states are distinguished and should not be merged. A link that resolves to a repository belonging to a method the paper compares against is a mis-attribution by the extraction, not a statement about the authors; those rows are marked as such and excluded from the announced-but-absent count. A repository that is the authors' own and empty is the consequential case, because a reader planning a comparison reads the announcement as a commitment.

4 extracted links carry no verdict and are omitted: they were not opened, and an unopened link is not evidence either way.

Table S9. Code availability per surveyed work, as read from the repository on the date recorded in Section 15. Repository state perishable and these verdicts are frozen at that date. 30 links opened across 30 works.

Method	Repository	Verdict
3D-ST	github.com/XuyangBai/PPF-FoldNet	not the authors' own repository
3DSR/DADA	github.com/VitjanZ/3DSR	authors' own, code present
AST	github.com/marco-rudolph/ast	authors' own, code present
BridgeNet	github.com/Xantastic/BridgeNet	authors' own, code present
CMDIAD	github.com/evenrose/CMDIAD	authors' own, code present
CMDR-IAD	github.com/ECGAIResearch/CMDR-IAD	not the authors' own repository
CPMF	github.com/caoyunkang/CPMF	authors' own, code present
DAS3D	github.com/SunnierLee/DAS3D	authors' own, code present
DCMUNet	github.com/TK941025/DCMUNet	repository announced but holds only a README and an images folder, no code
G2SF	github.com/ctaoaa/G2SF	authors' own, code present
GLFM	github.com/hustCYQ/GLFM-Multi-class-3DAD	authors' own, code present
Group3AD	github.com/M-3LAB/Group3AD	No, only empty repo
GS-CLIP	github.com/zhushengxinyue/GS-CLIP	authors' own, code present
HGAD	github.com/xcyao00/HGAD	authors' own, code present
IMRNet	github.com/Chopper-233/Anomaly-ShapeNet	authors' own, code present
LPFSTNet	github.com/YahuiCheng/LPFSTNet	Not uploaded yet
MAFR	github.com/adabrh/MAFR	authors' own, code present
MC4AD	github.com/eliahuhorwitz/3D-ADS	not the authors' own repository
MISDD	github.com/SvyJ/MISDD-MM	authors' own, code present
MVFM-3DAD	github.com/lil-wayne-0319/MV3D-AD	authors' own, code present
MVP-PCLIP	github.com/hustCYQ/MVP-PCLIP	authors' own, code present

Method	Repository	Verdict
PASDF	github.com/ZZZBBBZZZ/PASDF	authors' own, code present
PointAD	github.com/zqhang/PointAD	authors' own, code present
Reg2Inv	github.com/CHen-ZH-W/Reg2Inv	authors' own, code present
Reg3D-AD	github.com/M-3LAB/Real3D-AD	Linkt to the dataset
Simple3D	github.com/hustCYQ/MiniShift-Simple3D	authors' own, code present
SplatPose	github.com/m-kruse98/SplatPose	authors' own, code present
SplatPose+	github.com/Yizhe-Liu/SplatPosePlus	authors' own, code present
TRD	github.com/hito2448/TRD	authors' own, code present
ZUMA	github.com/yif-ma/ZUMA	authors' own, code present

S10. Extended Method Descriptions

The mechanism sections of the article carry the claims the survey goes on to use. The 25 descriptions here carry the architecture behind them, and they arrive by two routes. Most describe a work the article names in one place and nowhere else, so the description is the only thing that would be lost by leaving it out. The rest belong to works the article does use, in a comparison table or a later argument, where the article states what the method contributes in a sentence and the mechanism is set out here rather than twice.

Either way, nothing in the article's argument is completed in this section, and every work appears in Table S4 with its venue and peer-review status like any other. They are grouped by the Axis-A mechanism of the section each came from.

S10.1. Feature Distance and Memory Matching (A1)

Group3AD: Group-Level Contrastive Grouping.

Group3AD [72] learns its grouping with a group-level contrastive objective [73] that pulls geometrically similar patches together and pushes dissimilar ones apart. The resulting structured memory bank is what supports retrieval over groups rather than over individual patches.

S10.2. Reconstruction and Restoration Residuals (A2)

From Section 5 of the article.

AARD: Geometry-Aligned and Anomaly-Aware Reconstruction.

Wu *et al.* [183] address the reconstruction shortcut problem with geometry-aligned and anomaly-aware constraints. The method aligns the reconstruction process to the underlying geometric structure of the 3D surface, preventing the network from taking trivial identity shortcuts that would pass anomalous inputs unchanged. An anomaly-awareness component provides explicit supervision on synthetic anomalous samples, training the network to distinguish between normal and anomalous reconstructions. Together, these constraints improve sensitivity to subtle geometric deformations that naive autoencoder approaches smooth over.

Vote3D-AD: Synthesis-Supervised Localization.

Vote3D-AD [173] trains a differentiable vote-clustering localizer on normal samples alone, supervised by a Varied Defect Synthesis generator that produces bulges, dents, holes, cracks, surface roughness, and simulated sensor artifacts. The synthesized defects supply dense point-level supervision, and the method reports improved localization over prior reconstruction baselines.

PCDiff: Instance-Level Diffusion.

PCDiff [184] couples a diffusion model with joint global and local reconstruction for instance-level detection. It conditions generation on multi-modal instance cues such as texture gradients, image patches, text, and masks, so that subtle deviations that escape category-level models still produce detectable reconstruction discrepancies.

EasyNet: A Reconstruction Pipeline Built for Speed.

For EasyNet [91], avoiding the computational overhead of feature extraction and nearest-neighbour search is what buys the throughput the article reports; the cost is that the representation is learned from scratch on each category rather than inherited from a pretrained backbone.

S10.3. Teacher–Student and Representation Discrepancy (A3)

From Section 6 of the article.

TS-Net: Few-Normal-Sample Distillation.

TS-Net [177] is an early PointNet-based teacher–student framework that operates directly on point clouds and is trained with only a few normal samples. A teacher pretrained on anomaly-free data supplies geometric descriptors that the student is distilled to reproduce, with the teacher–student discrepancy serving as the per-point anomaly signal. The few-shot training regime targets industrial settings where large collections of defect-free scans are unavailable.

Hierarchical Asymmetric Student–Teacher.

The Hierarchical Asymmetric Student–Teacher framework [180] builds asymmetry into a masked-autoencoder student–teacher pair through a hierarchical structure. A contrastive loss further discourages the student from trivially replicating the teacher on anomalies, sharpening the feature discrepancy used for scoring. The method is evaluated on MVTec 3D-AD [5].

MDSS: Memoryless Multi-Modal Distillation.

The MDSS framework [52] extends the teacher–student paradigm to multi-modal 3D anomaly detection while explicitly avoiding memory banks. It combines knowledge distillation with signed distance function (SDF) learning, where the SDF component provides geometric reasoning about surface deviations. The anomaly maps from both the distillation discrepancy and the SDF branch are fused using a product-of-maxima strategy, yielding a joint anomaly map that captures both appearance and geometric anomalies.

Dropping the memory bank removes the retrieval term from MDSS inference, with the caveat Section 7 sets out for the same property in flows.

S10.4. Registration and Template Deviation (A5)

From Section 8 of the article.

Looking3D.

Looking3D [171] inverts the direction of alignment: 2D query images are aligned to reference 3D shapes. Using transformer-based feature alignment, the method establishes viewpoint-invariant local patch correspondences between 2D images and 3D models. Training on a large synthetic dataset (BrokenChairs-180K with 180K anomalous images and 8K 3D shapes) enables generalization to diverse object categories.

Pose-Agnostic Detection in Cluttered Scenes.

Pose-Agnostic Detection in Cluttered Scenes [176] extends pose-agnostic alignment, building on the SplatPose family of Gaussian-splatting pose-estimation pipelines, to realistic settings with background clutter, and introduces a synthetic conveyor-belt dataset for evaluation. Unlike most methods in this section, it is an image-based pipeline that operates on rendered RGB views rather than raw point clouds. At the time of writing, it remains a preprint.

SplatPose+: Real-Time Pose-Agnostic Detection.

SplatPose+ [150] succeeds SplatPose [58] and targets the cost that made its predecessor impractical inline. Where the earlier method fits a Gaussian-splat representation and solves for pose per query, SplatPose+ restructures that pipeline for real-time operation while keeping the pose-agnostic property, and reports 96.4% image-level AUROC and 96.9% AUPRO on the pose-agnostic benchmark. It is not in Table 10 or Table 11 because it evaluates on neither: the pose-agnostic family uses its

own benchmark, which is the point of that family and also a reason its methods are hard to compare against the rest of this survey.

SplatPose.

SplatPose [58] brings 3D Gaussian Splatting [174], a technique for real-time novel view synthesis, to anomaly detection. Given multi-view training images, a 3D Gaussian Splatting model is trained to represent the normal object. At test time, the model is used for differentiable pose estimation, aligning arbitrary test views to the learned 3D representation. Anomalies are detected by comparing rendered normal views with actual test images at the estimated pose. Detection is therefore pose-agnostic without an explicit registration step.

3DKeyAD: Keypoint-Guided Clustering.

3DKeyAD [57] preprocesses normal prototypes into keypoint-centred clusters that serve as structured reference units, and the cluster-wise comparison at inference uses both raw coordinates and FPFH descriptors.

S10.5. Semantic and Foundation-Model Transfer (A6)

From Section 9 of the article.

CLIP3D-AD.

In the few-shot setting, CLIP3D-AD [186] extends CLIP by rendering each point cloud into multi-view normal and anomalous images and adapting the frozen CLIP features with lightweight adapters. The synthesized anomalous renderings supervise the adapter layers, so visual features align with anomaly-aware text prompts from only a handful of normal samples on MVTec 3D-AD.

PLANE: Point-Language Dual-Prompts.

For multi-class detection, PLANE [181] adapts pretrained point-language models by learning paired text and point prompts that jointly specify normal and anomalous appearance. To supervise the prompts, it introduces an Ano3D pseudo-anomaly generator that synthesizes diverse anomalies directly on the point cloud, supplying the contrastive signal without real defect annotations. One model therefore covers many object categories, where the field's default is a separate detector per class.

DMP-3DAD.

DMP-3DAD [182] is training-free and cross-category, working from only a few normal samples by projecting point clouds into realistic multi-view depth maps. A frozen CLIP encoder scores anomalies directly over these depth renderings, sidestepping the per-category prompt tuning that other CLIP-based methods rely on. Because the score is computed in depth space, no per-category prompt tuning is needed and the model transfers across object types without retraining.

CoGeoAD.

CoGeoAD [185] fuses paired color and geometry renderings of the same object through a data-driven multi-view attention mechanism. The attention module learns to weight and combine the appearance and shape cues across views, producing an anomaly signal that neither modality yields alone on MVTec 3D-AD or Eyecandies.

GS-CLIP: Geometry-Aware Prompts and Synergistic View Representations.

GS-CLIP [62] targets two weaknesses of projection-based zero-shot detection: the geometric detail lost in 3D-to-2D projection and the reliance on a single 2D representation. Its first stage dynamically generates text prompts embedded with 3D geometric priors, combining global shape context with local defect information distilled by a Geometric Defect Distillation Module, so the CLIP text–image matching becomes sensitive to geometric anomalies that appearance-only prompts miss. Its second stage processes rendered and depth images in parallel and fuses the two streams with a Synergistic Refinement Module, exploiting their complementary strengths.

S10.6. Modality and Fusion (Axis B)

From Section 10 of the article.

DBRN: Dual-Branch Reconstruction.

The Dual-Branch Reconstruction Network [100] reconstructs RGB and depth in separate branches linked by cross-branch connections, with learned importance weights that rebalance each modality's contribution according to the input.

G2SF: Geometry-Guided Score Fusion.

G2SF [179] improves multi-modal fusion by using local geometric structure to guide the combination of anomaly scores from different modalities. Rather than treating RGB and 3D scores as equal contributors, G2SF adapts fusion weights based on local surface properties, upweighting geometric evidence in regions with complex curvature and appearance evidence in texture-rich, geometrically simple areas. This geometry-aware score fusion improves robustness to sensor noise and surface reflectance variation that unweighted fusion strategies handle poorly.

CMDR-IAD: Cross-Modal Mapping and Dual-Branch Reconstruction.

Daci *et al.* [172] address the misalignment between 2D image and 3D point cloud feature spaces by mapping both into a shared representation, then reconstructing each modality in its own branch. Anomalies show up as reconstruction error in the mapped and the original feature spaces alike.

TRD: Tuned Reverse Distillation.

TRD [107] extends reverse distillation to the multi-modal setting with per-modality student–teacher branches, linked by cross-modal tuners (a Crossmodal Filter and Amplifier) that exchange RGB and 3D cues during distillation so each modality reinforces the other's reconstruction signal.

CMDIAD: Cross-Modal Distillation.

CMDIAD [178] distils knowledge across modalities at training time so that inference can proceed on fewer of them, which is what lets it survive a sensor stream going missing.

Text-Guided Unified Detection.

Li *et al.* [175] introduce a unified RGB-3D-text model that breaks the conventional one-model-per-class setting. A geometry-aware cross-modal mapper aligns appearance and geometry, while an object-conditioned textual feature adaptor injects category semantics, allowing a single model to detect anomalies across multiple object classes.

M3DM-NR: CLIP-Based Denoising.

In M3DM-NR [123] the corrupted depth arises on reflective surfaces, transparent materials, and at the limits of the sensor's range. CLIP [76] features identify the affected regions and down-weight them before the memory bank is populated.

S11. Charted Views of the Tabulated Data

Neither figure here carries a number the article does not print. Figure S1 charts the category and sample counts of Table 9, and Figure S2 charts the reported values of Tables 10 and 11. They are kept because a chart makes the spread across benchmarks visible at a glance in a way a column of numbers does not, and moved because that is a convenience rather than evidence.

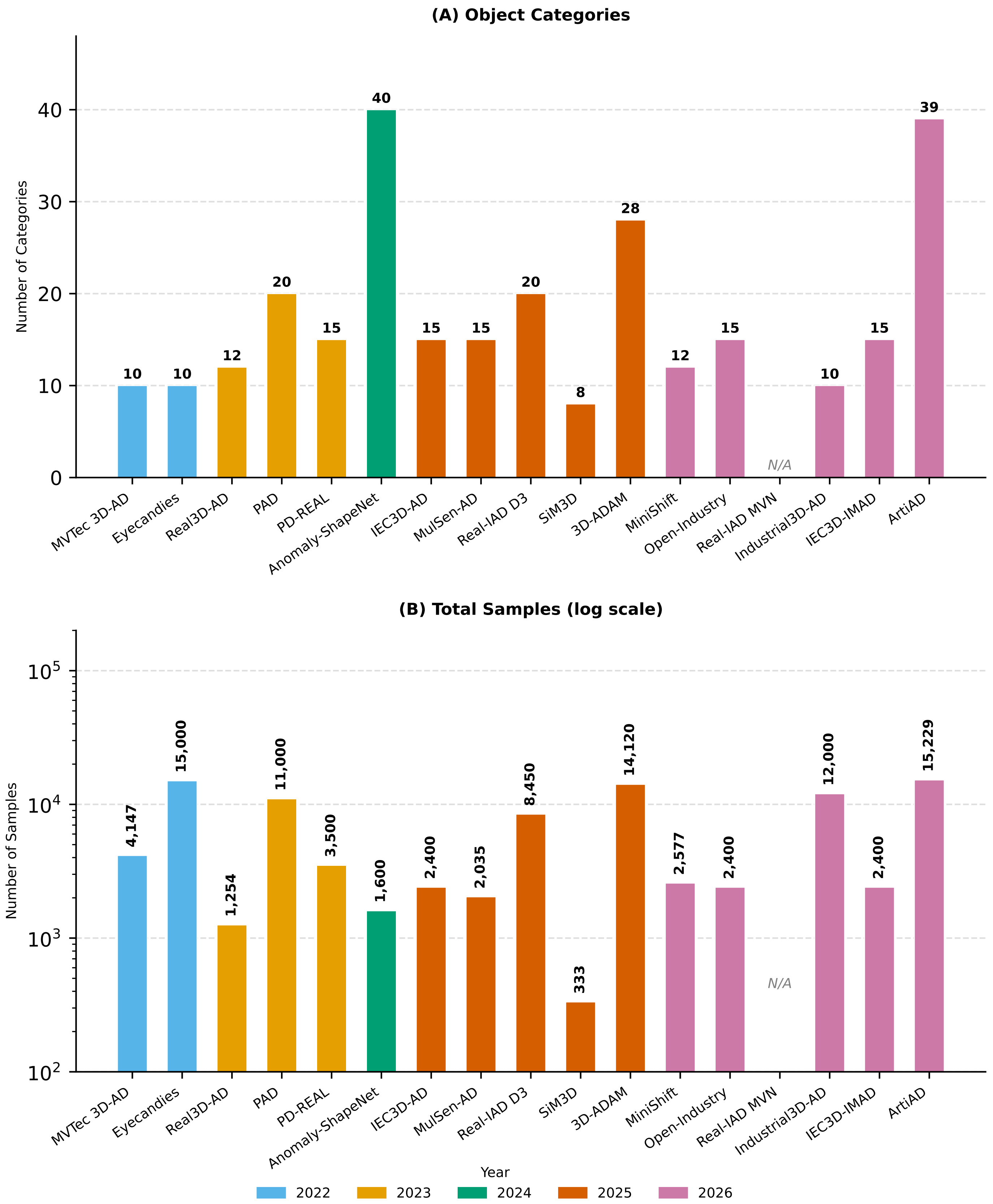

Figure S1. Comparison of 3D anomaly detection datasets by (A) number of object categories and (B) total number of samples (log scale). Colors indicate the year of publication. N/A marks a count the source does not state; Real-IAD MVN is the only such entry, and it states neither. Anomaly-ShapeNet provides the broadest category coverage (40), and ArtiAD the largest sample count (15,229).

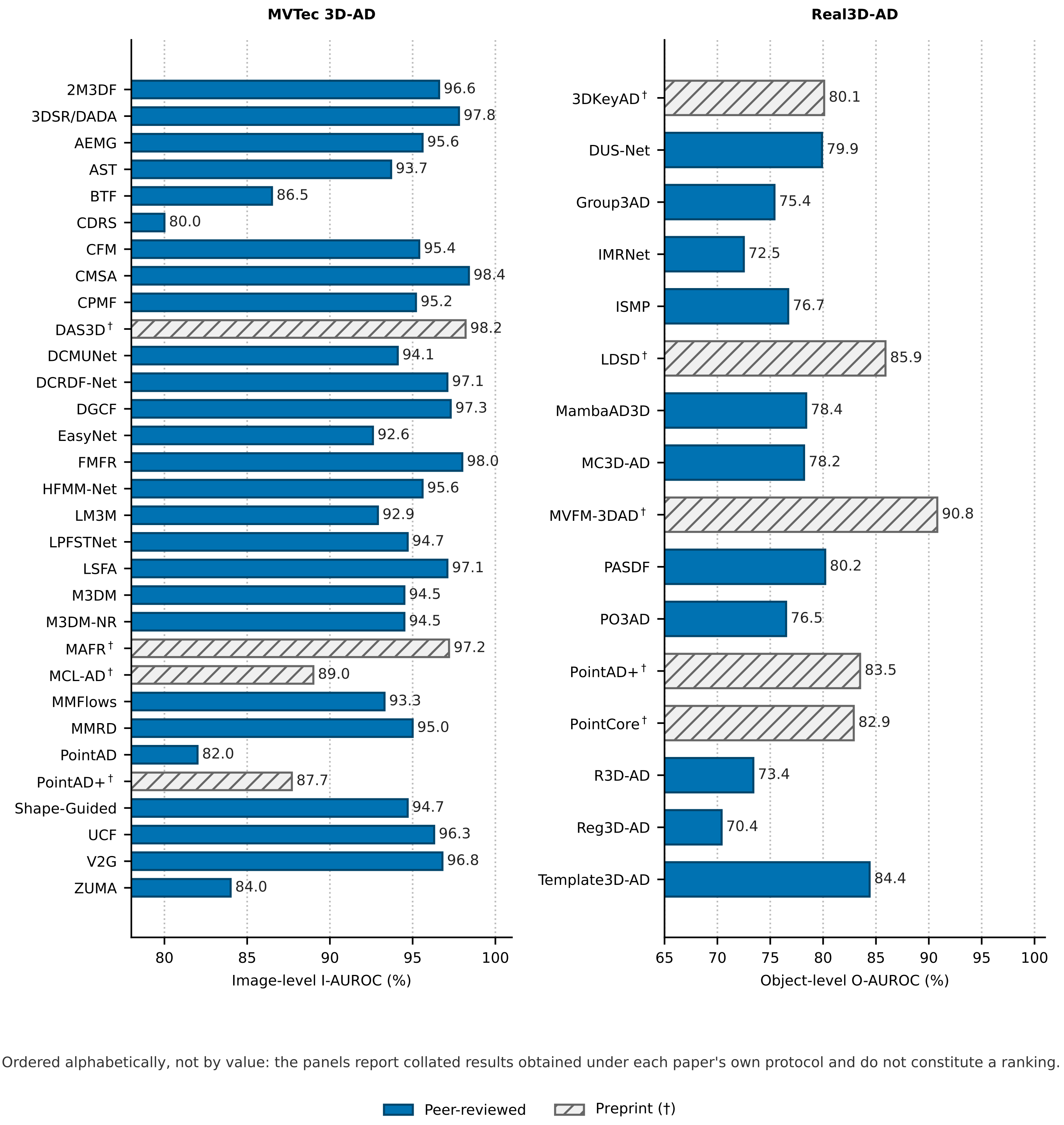

Figure S2. Reported values on MVTec 3D-AD (image-level I-AUROC, left) and Real3D-AD (object-level O-AUROC, right), collated from the original publications (Tables 10 and 11). Methods are ordered *alphabetically, not by value*, since each number was obtained under its own paper's protocol. Solid bars are peer-reviewed; hatched bars marked with a dagger are preprints, so the distinction survives grayscale reproduction.

S12. Evaluation Protocol per Reported Result

Table S10 carries, for every work tabulated in Section 13 of the article, the evaluation protocol as its own paper states it. The article's result tables print a compressed form of the preprocessing column so that they stay readable; this is what that column was compressed from, together with the fields the article has no room for. Every row records where in the source it was read. A blank cell means the source does not state the field, which is a property of the reporting rather than of our

reading, and the density of blanks in the metric and entity columns is why the article declines to treat identically named metrics as one quantity.

Table S10. Evaluation protocol per work and benchmark, quoted from each source. *Metrics*: the labels the paper prints, with the per-region integration limit where it states one. *Entity*: what a localisation score is computed over. *Preprocessing*: registration, resampling and input sizing before scoring. An en dash marks a field the source does not state.

Method	Metrics as printed	Entity	Backbone	Preprocessing	Read from
Anomaly-ShapeNet					
DUS-Net [102]	O-AUROC; O-AUPR; P-AUROC; P-AUPR	point	PointNet encoder + transformer decoder; Down-Net pre-trained on ShapeNetPart, Up-Net on a Visionair subset	G = 8192 groups of K = 640 points via FPS + KNN; Up-Net up-sampling rate gamma = 8	Sec. 4.1 p. 4; Tables 1 and 3
Group3AD [72]	O-AUROC	point	Point-MAE pretrained encoder, reinforced by IUN and IAN	unstated (4096 groups of 128 points is given only for the Real3D-AD airplane, App. C p. 12)	Table 8 p. 12; App. D p. 12
IMRNet [49]	I-AUROC (limit unstated)	pixel	Point-MAE (pretrained on ShapeNet-55)	GPS to 256 patch centres x 64 KNN neighbours; samples have 8,000-30,000 points	Sec. 5.1 p. 7; Table 3 p. 7
ISMP [135]	P-AUROC	pixel	PointMAE; EfficientNet; FPFH	FPS to a set of centre points with k-nearest-neighbour patches; number of centres not stated	Sec. Implementation p. 5; Table 2
LDSD [105]	O-AUROC; P-AUROC	point	none (learnable LOD feature volumes + 4-layer MLP SDF-Net)	100,000 surface points (base number in NPG)	Sec. 4.1 p. 5; Tables 1-2 p. 6-7; Sec. 3.3 p. 4
MambaAD3D [89]	AUROC (object level); AUPR (object level); AUROC (point level); AUPR (point level)	point	Point-MAE pretrained on ShapeNet, frozen (L = 6 layers, D = 768)	voxel-grid filtering at 0.001 m; FPS to M = 256 patch centers, K_max = 512 neighbours	Sec. 4.2 p. 9; Table 1
MC3D-AD [18]	O-AUROC; P-AUROC	unstated	PointMAE (pre-trained on ModelNet40)	FPS to group centers (count unstated); KNN groups	Table 2 p. 842; Sec. 4.2
PASDF [50]	O-AUROC; P-AUROC	point	none (SDF MLP trained per category; FPFH used for RANSAC)	voxel-grid downsampling inside PAM (voxel size not stated)	Tables 2-3 p. 6; Sec. 4.3
PO3AD [59]	object-level AUC-ROC; point-level AUC-ROC	point	MinkUNet34C (pretraining not stated)	none (paper: no point cloud downsampling); voxel size 0.03 for MinkUNet	Tables 1-2 p. 5-6; Sec. 4.2
R3D-AD [36]	I-AUROC	point	none	randomly downsampled to a fixed size of 2048 points	Table 2 p. 11; Table 7 (appendix A.3) p. 21; Sec. 4.3 p. 10
Template3D-AD [56]	I-AUROC	point	PointMAE (ShapeNet-pretrained, layers {3,7,11}); FPFH	voxel-grid downsampling (voxel size 0.05), then FPS to N=4096 center points; S=128 nearest points per group	Table 2 p. 1634; Sec. 4.3 p. 1634
MVTec 3D-AD					
2M3DF [65]	I-AUROC; AUPRO (limit unstated)	pixel	WideResNet-50 (third layer block, ImageNet); PointNet++ (segmentation network, d3D=128)	RANSAC background removal (points within distance 0.005 of plane eliminated). Input 224 × 244 (as printed)	Sec. IV-B, IV-D p. 6809; Table I p. 6810

Method	Metrics as printed	Entity	Backbone	Preprocessing	Read from
3D-ST [51]	AU-PRO (limit both)	pixel	none (own teacher network self-supervised on synthetic ModelNet10 scenes)	farthest point sampling to n = 64000 points	Sec. 4.1 p. 9-10; Table 1 p. 10; Table 2 p. 19
3DSR/DADA [48]	image-level AUROC; PRO; mean pixel-level AUROC (limit unstated)	pixel	DADA (pretrained on simulated depth + ImageNet RGB); VQ codebooks 2048 x 256	sorted point cloud used as a depth image; missing values filled from 3x3 valid-pixel average	Sec. 4 p. 5; Sec. 4.1 p. 5
AEMG [85]	I-AUROC; P-AUROC; AUPRO@30%; AUPRO@1% (limit both)	pixel	Wide-ResNet50-2 (RGB); PointNet SDF/NIF (3D)	FPS local patches, K = 500 points per patch, overlap rate 10, ~155 patches for a ~7,750-point sample. Input 224 x 224	Sec. IV-B p. 1139; Table VI p. 1143
AST [29]	AUROC; pixel-AUROC	pixel	EfficientNet-B5 layer 36 (ImageNet)	Input 768x768 (RGB); depth masks resized to 192x192 then pixel-unshuffled (d=8)	Table 3 p. 7; Table 4 p. 7; Sec. 4.2.1-4.2.2 p. 5
BTF [23]	I-ROC; P-ROC; PRO (limit 0.3)	pixel	FPFH (hand-crafted, Open3D normals); ImageNet-pretrained WideResNet50 blocks 2-3 via PatchCore	organized point cloud and RGB downsampled to 224x224 (nearest-neighbour / bicubic); 28x28 = 784 patches per sample. Input 224x224	Sec. 3.4 p. 4; Sec. 4.5 p. 8; App. E.1
CDRS [131]	image-level ROCAUC; pixel-level ROCAUC; AU-PRO (limit unstated)	pixel	DINO ViT-B/8 (RGB); Point-MAE (3D); released UFF fusion module	1024 groups with group size 128; at least 1024 valid points after preprocessing. Input 224 x 224	Sec. 3.8 p. 7-8; Table 2 p. 9; Sec. 4.1 p. 8
CFM [133]	I-AUROC; P-AUROC; AUPRO@30%; AUPRO@1% (limit both)	pixel	DINO ViT-B/8 (ImageNet); Point-MAE (ShapeNet), both frozen	1024 groups of 32 points obtained with FPS. Input 224x224	Sec. 4 p. 5; Tables 1-2, 5
CMSA [124]	I-AUROC; AUPRO@30%; AUPRO@1% (limit both)	pixel	DINO ViT-B/8 (ImageNet); Point Transformer (ShapeNet-pretrained)	FPS into 1024 groups of 32 points; point-cloud descriptors downsampled to a 56 x 56 grid. Input 224 x 224	Implementation Details p. 1595; Table 1
CPMF [17]	I-AUC; P-PRO (limit unstated)	pixel	ResNet18 (first three blocks, ImageNet); FPFH handcrafted	PCD downsampled before FPFH computation (factor not stated). Input 224 × 224 (rendered multi-view images)	Table I-II p. 7 (pdf); Sec. IV-A.2-3 p. 6-7
DAS3D [37]	I-AUROC; P-AUROC; AUPRO (limit unstated)	pixel	none named; three UNet-like networks (two reconstruction + one discriminator)	Input 256x256	Sec. 4.1 Data Preprocess p. 11; Tables 1-2, 5
DCMUNet [99]	I-AUROC; P-AUPRO (limit unstated)	pixel	none	Input 256x256	Table XI p. 14; Sec. IV-B p. 9; Sec. IV-F p. 14
DCRDF-Net [109]	I-AUROC; PRO (limit unstated)	pixel	ResNet-50 (ImageNet, frozen teacher)	Input 256x256	Table 1 p. 11; Table 2 p. 11; Sec. 4.1 p. 9
DGCF [125]	I-AUROC; P-AUROC; AUPRO (limit 0.3)	pixel	WideResNet50	Input 256 x 256	Sec. 4.3 p. 9; Table 1
EasyNet [91]	I-AUROC; Pixel AUC; Pixel AP; AUPRO (limit unstated)	pixel	none	RANSAC background plane removal (points within 0.005 distance eliminated). Input 256 × 256	Table 1 p. 6; Table 4 p. 7; Table 7 (supplement) p. 11; Sec. 7.4 p. 10
FMFR [126]	I-AUROC; AUPRO@30%; AUPRO@1% (limit both)	pixel	DINO ViT-B/8 (ImageNet); PointMAE (ShapeNet)	FPS with 1024 groups, group size 128. Input 224x224	Tables 1-3 pp. 240-241; Sec. 4 p. 239

Method	Metrics as printed	Entity	Backbone	Preprocessing	Read from
Group3AD [72]	O-AUROC	point	M3DM (PointMAE) with IUN and IAN added	–	Table 7 p. 12; App. D p. 12
HFMM-Net [132]	I-AUROC; P-AUROC; AUPRO@30%; AUPRO@1% (limit both)	pixel	DINO patch partitioning/projection (RGB); lightweight PointNet patch embedding (3D); bidirectional Mamba encoders (no pretrained weights stated)	RANSAC background plane removed (points within 0.005 m); point cloud and RGB resampled or cropped to 224 x 224. Input 224 x 224	Sec. 4.1 p. 10-11
LM3M [130]	image-level AUROC; pixel-level AUROC; AU PRO (limit unstated)	pixel	self-supervised Vision Transformer (RGB, structurally pruned); Point Transformer (3D, 4-bit PTQ)	local groups by farthest point sampling and nearest-neighbour grouping (no counts printed)	Sec. IV-A p. 5-6
LPFSTNet [108]	I-AUROC; P-AUROC	pixel	EfficientNet-B5 (ImageNet) as teacher feature extractor	Input 768 × 768	Sec. 4.3 p. 7; Table 1 p. 8; Table 2 p. 8
LSFA [84]	I-AUROC; AUPRO; P-AUROC (limit unstated)	pixel	DINO ViT-B/8 (ImageNet); point transformer / PointMAE pre-trained on ShapeNet (layers 3/7/11)	Input 224x224	Table 1 p. 6; Table 2 p. 7; Sec. 4.1 p. 6
M3DM [22]	I-AUROC; AUPRO; P-AUROC (limit unstated)	pixel	DINO ViT-B/8 (ImageNet); Point Transformer / Point-MAE pre-trained on ShapeNet (layers 3,7,11)	FPS into M=1024 point groups of S=128 points. Input 224x224	Table 1 p. 6; Table 2 p. 7; Table 3 p. 7; Sec. 4.1 p. 5
M3DM-NR [123]	I-AUROC; AUPRO; P-AUROC (limit unstated)	pixel	CLIP ViT-H/14 (LAION-2B); Point-BIND; DINO ViT-B/8 (ImageNet); Point Transformer (ShapeNet), layers {3,7,11}	FPS-based multi-scale point-cloud patches; minimum threshold theta = 128 points per patch. Input 224 x 224	Sec. 4.1 p. 8-9; Tables 1-2
MAFR [127]	I-AUROC; P-AUROC; AUPRO@30%; AUPRO@1% (limit both)	pixel	DINO ViT-B/8; PointMAE	Input 224 × 224	Sec. IV-A p. 5; Table I p. 6; Table III p. 7
MCL-AD [64]	I-AUROC; P-AUROC; AUPRO (limit unstated)	point	CLIP image and text encoders (layers 6/12/18/24 used)	point cloud rendered into 9 multi-view depth images by rotating about the X and Y axes. Input 336 x 336	Sec. IV-A implementation details p. 8; Table I
MMFlows [55]	I-AUCROC; P-AUPRO; P-AUCROC (limit unstated)	pixel	DINO ViT-B/8 (ImageNet); Point Transformer (Pang et al. 2022) pre-trained on ShapeNet	FPS; Point Transformer groups 1 024 of size 128	Table 1 p. 460; Tables 2-3 p. 461; Sec. 2 (Chinese) and English abstract
MMRD [53]	Image-level AUROC; ROC_AD; Pixel-level PRO; ROC_AL; PRO (limit unstated)	pixel	pre-trained WideResNet50 teacher; student as in RD	Input 256x256	Implemental Details p. 8450; Tables 1(a) and 3
MVFM-3DAD [54]	O-AUROC; P-AUROC	point	DINOv2 ViT-B/14 (frozen)	Input 224x224 (rendered views)	Table 2 p. 9; Sec. 4.1
PointAD [20]	I-AUROC; P-AUROC; AUPRO (limit unstated)	point	CLIP ViT-L/14@336px (open_clip; frozen)	Input 336x336	Table 1 p. 6; Sec. 4.2-4.3; Appendix A

Method	Metrics as printed	Entity	Backbone	Preprocessing	Read from
PointAD+ [63]	I-AUROC; AP; P-AUROC; AUPRO (limit unstated)	point	CLIP ViT-L/14@336px (open_clip), frozen	9 views rendered by rotating along the X-axis; background plane of point clouds removed. Input 336 x 336	Sec. 4.1 p. 9-10; Table 1 p. 10
Shape-Guided [46]	Img-AUROC; Pix-AUROC; AUPRO (limit both)	pixel	simplified PointNet + Neural Implicit Function (SDF); Wide ResNet-50-2	local patches of 500 points (K=500) via FPS + K-nearest neighbors; about 7,500 points per sample. Input 224x224	Sec. 4.1-4.3 pp. 5-6; Tables 1-3
UCF [128]	I-AUROC; P-AUPRO0.3; P-AUPRO0.01 (limit both)	pixel	DINO ViT-B/8 (ImageNet); Point-MAE (ShapeNet)	RANSAC-fitted plane background removal (points closer than 0.005 treated as background)	Sec. IV-A.2-3 p. 5005; Table I-II p. 5005
V2G [98]	I-AUROC; AUPRO; AUPRO(0.3); AUPRO(0.05) (limit 0.3)	pixel	DINO ViT-B/8 (ImageNet); Point-MAE (ShapeNet), both frozen	1024 groups of 32 points sampled with FPS then KNN; features interpolated to 224x224x1152. Input 224x224	Sec. IV-A2 p. 3674; Sec. IV-A5 p. 3675; Table 3
ZUMA [121]	I-AUC; P-AUC; PRO (limit unstated)	pixel	CLIP ViT-L/14@336px (frozen)	–	Table I p. 6607; Sec. IV-A
Real3D-AD					
3DKeyAD [57]	O-AUROC; P-AUROC	point	none (raw coordinates + FPFH handcrafted; PointMAE only in ablation variants)	prototypes downsampled to 20,000 points; test sample to 1,000 points	Sec. 4.2 p. 5; Tables 1-2 p. 6
DUS-Net [102]	O-AUROC; O-AUPR; P-AUROC; P-AUPR	point	PointNet encoder + transformer decoder; Down-Net pre-trained on ShapeNetPart, Up-Net on a Visionair subset	G = 8192 groups of K = 640 points via FPS + KNN; Up-Net up-sampling rate gamma = 8	Sec. 4.1 p. 4; Table 2, Table 4
Group3AD [72]	O-AUROC; O-AUPR; P-AUROC; P-AUPR	point	Point-MAE pretrained encoder, reinforced by IUN and IAN	4096 groups per point cloud, 128 neighbouring points each; 524288 available points (airplane example)	Sec. 4.1 p. 6; App. A p. 11; App. C p. 12
IMRNet [49]	I-AUROC (limit unstated)	pixel	Point-MAE (pretrained on ShapeNet-55)	GPS to 256 patch centres x 64 KNN neighbours; backbone pretrained at point size 8192	Sec. 5.3 p. 7; Table 2 p. 7
ISMP [135]	O-AUROC; P-AUROC	pixel	PointMAE; EfficientNet; FPFH	FPS to a set of centre points with k-nearest-neighbour patches; number of centres not stated	Sec. Implementation p. 5; Table 1; Fig. 2 caption
LDSD [105]	O-AUROC; P-AUROC	point	none (learnable LOD feature volumes + 4-layer MLP SDF-Net)	100,000 surface points (base number in NPG)	Sec. 4.1 p. 5; Tables 3-4 p. 7-8
MambaAD3D [89]	AUROC (object level); AUPR (object level); AUROC (point level); AUPR (point level)	point	Point-MAE pretrained on ShapeNet, frozen (L = 6 layers, D = 768)	voxel-grid filtering at 0.0005 m; FPS to M = 256 patch centers, K_max = 512 neighbours	Sec. 4.1 p. 8; Sec. 4.2 p. 9; Table 2
MC3D-AD [18]	O-AUROC; P-AUROC	unstated	PointMAE (pre-trained on ModelNet40)	FPS to group centers (count unstated); KNN groups	Table 1 p. 841; Sec. 4.1-4.2
MVFM-3DAD [54]	O-AUROC; P-AUROC	point	DINOv2 ViT-B/14 (frozen)	Input 224x224 (rendered views)	Table 1 p. 8; Sec. 4.1
PASDF [50]	O-AUROC; P-AUROC	point	none (SDF MLP trained per category; FPFH used for RANSAC)	voxel-grid downsampling inside PAM (voxel size not stated)	Table 1 p. 5; Sec. 4.1-4.2
PO3AD [59]	object-level AUC-ROC	–	MinkUNet34C (pretraining not stated)	none (paper: no point cloud downsampling); voxel size 0.03 for MinkUNet	Table 3 p. 7; Sec. 4.4.2

Method	Metrics as printed	Entity	Backbone	Preprocessing	Read from
PointAD [20]	I-AUROC; P-AUROC; AUPRO (limit unstated)	point	CLIP ViT-L/14@336px (open_clip; frozen)	Input 336x336	Table 1 p. 6; Appendix A
PointAD+ [63]	I-AUROC; AP; P-AUROC	point	CLIP ViT-L/14@336px (open_clip), frozen	9 views rendered by rotating along the X-axis. Input 336 x 336	Table 1 caption p. 10
PointCore [71]	O-AUROC; P-AUROC	point	PointMAE	greedy down-sampling to Smax points (Smax not stated)	Table II p. 4; Sec. III-A
R3D-AD [36]	I-AUROC; P-AUROC	point	none	randomly downsampled to a fixed size of 4096 points	Table 1 p. 10; Table 3 p. 12; Sec. 4.3 p. 10
Reg3D-AD [19]	object-level AUROC (O-AUROC); point-level AUROC (P-AUROC); AUPR	point	PointMAE features plus raw xyz coordinate features	point groups 16384; coreset-sampled memory bank of size 10000	Sec. 3.2 Data Statistics p. 6; Sec. 4.1 p. 7; Table 4; Table 8 (appendix)
Template3D-AD [56]	I-AUROC; P-AUROC	point	PointMAE (ShapeNet-pretrained, layers {3,7,11}); FPFH	voxel-grid downsampling (voxel size 0.25), then FPS to N=4096 center points; S=128 nearest points per group	Table 1 p. 1634; Table 4 p. 1636; Sec. 4.3 p. 1634

S13. Computational Cost as Each Paper Reports It

Table S11. Computational cost as reported by the surveyed papers for their own methods. Every cell is quoted from the source; an en dash marks a field the paper does not state, and the density of en dashes is the finding (Section 13.6). *P, R, F, M*: whether the timed span includes preprocessing, registration, feature extraction and memory search: a tick where the paper says it does, a cross where it says it does not, an en dash where it does not say. *Scope*: whether the figure is model-only or end-to-end. Figures are not converted between units, so a paper reporting throughput and one reporting latency stay in their own columns. Long values are abbreviated to fit; the complete extraction, one row per figure with its location in the source and a supporting quote, is released with this article.

Method	Hardware	Batch	Points / res.	P	R	F	M	Scope	Latency	Throughput	Params	Memory
2M3DF [65]	NVIDIA RTX 4090	–	224 × 244 (as…	✓	–	✓	–	end-to-end	–	29.8 (FPS)	–	578.46 (MBs)
3D-ST [51]	NVIDIA Tesla V100	–	64000 points (64k)…	–	–	–	–	–	0.049 s	–	–	2.71 GB…
3D-ST [51]	NVIDIA Tesla V100	–	64000 points (64k)…	–	–	–	–	–	0.093 s	–	–	5.14 GB…
3DSR/DADA [48]	NVIDIA RTX A4500 GPU	–	–	–	–	–	–	–	–	33 FPS	–	–
3DzAL [60]	–	–	H=W=224 (28x28=784…	–	–	–	–	–	–	–	13.6M	234M (memory…
ActiveInspect [147]	eight A100 80 GB GPUs	–	–	–	–	–	–	–	–	–	–	–
ActiveInspect [147]	single A100 80 GB	1	–	✓	–	–	–	end-to-end	3.2 s	–	–	peak memory…
ActiveInspect-adaptive [147]	single A100 80 GB	1	–	✓	–	–	–	end-to-end	2.9 s	–	–	peak memory…
AEMG [85]	NVIDIA GeForce RTX…	–	224 x 224; K = 500…	–	–	–	–	–	–	0.632 FPS	–	3820.00 MB
AEMG [85]	NVIDIA GeForce RTX…	–	224 x 224	–	–	–	–	–	–	3.667 FPS	–	1742.00 MB
AEMG [85]	NVIDIA GeForce RTX…	–	K = 500 points per…	–	–	–	–	–	–	0.719 FPS	–	2104.00 MB
AEMG [85]	NVIDIA GeForce RTX…	–	K = 500 points per…	–	–	–	–	–	1.58 (unit…	–	–	–
APG-Net [86]	single NVIDIA GeForce…	–	224 x 224 images…	–	–	–	–	–	–	1.18 FPS	107.03	–
AST [29]	NVIDIA RTX 1080 Ti	–	768x768	–	–	–	–	–	3.4 ms	–	26.0 M	–
AST [29]	NVIDIA RTX 1080 Ti	–	768x768	–	–	–	–	–	6.1 ms	–	44.8 M	–
AST [29]	NVIDIA RTX 1080 Ti	–	768x768	–	–	–	–	–	10.4 ms	–	82.6 M	–
AST [29]	NVIDIA RTX 1080 Ti	–	768x768	–	–	–	–	–	19.8 ms	–	151.1 M	–
AST [29]	NVIDIA RTX 1080 Ti	–	768x768	–	–	–	–	–	29.4 ms	–	233.6 M	–
AST [29]	NVIDIA RTX 1080 Ti	–	768x768	–	–	–	–	–	4.5 ms	–	3.8 M	–
baselines (PatchCore/EfficientAD/AST/BTF/CFM/M3DM) [146]	–	–	1540x1540x3	–	–	–	–	–	–	–	–	–
BridgeNet [67]	NVIDIA Tesla V100	–	–	–	–	–	–	–	–	25 FPS	–	2289 MB
BTP [66]	a single NVIDIA RTX…	–	1024 points (FPS…	–	–	–	–	–	–	75.3 FPS	–	–
BTP [66]	a single NVIDIA RTX…	–	2048 points (FPS…	–	–	–	–	–	–	73.8 FPS	–	–
BTP [66]	a single NVIDIA RTX…	–	4096 points (FPS…	–	–	–	–	–	–	71.9 FPS	–	–
BTP [66]	a single NVIDIA RTX…	–	8192 points (FPS…	–	–	–	–	–	–	69.8 FPS	–	–
C3D-AD [104]	NVIDIA A100-PCIE-40GB…	–	–	–	–	–	–	–	0.281 (s)	–	–	4.431 (GB)
CFM (Ours) [133]	NVIDIA GeForce RTX…	–	224x224 images…	✓	–	✓	–	end-to-end	–	21.755 fps	–	437.91 MB
CFM (Ours-M) [133]	NVIDIA GeForce RTX…	–	224x224 images…	✓	–	✓	–	end-to-end	–	24.146 fps	–	295.81 MB
CFM (Ours-S) [133]	NVIDIA GeForce RTX…	–	224x224 images…	✓	–	✓	–	end-to-end	–	24.527 fps	–	211.09 MB
CFM (Ours-T) [133]	NVIDIA GeForce RTX…	–	224x224 images…	✓	–	✓	–	end-to-end	–	42.818 fps	–	48.12 MB

Method	Hardware	Batch	Points / res.	P	R	F	M	Scope	Latency	Throughput	Params	Memory
CM3D-AD [92]	Raspberry Pi 4 ...	–	–	–	–	✓	–	–	6.341s	–	20.91 MB ...	305.52 MB ...
CM3D-AD [92]	Jetson Nano (2 GB)	–	–	–	–	✓	–	–	1.905s	–	20.91 MB ...	555 MB (RAM)
CM3D-AD [92]	2x NVIDIA A100 (40GB) ...	128	–	–	–	–	–	end-to-end ...	approximately ...	–	20.91 MB	–
CMDR-IAD [172]	NVIDIA A100 GPU ...	–	–	✓	–	✓	–	end-to-end	–	2.710 (FPS)	–	2797.65 (MB)
CMDR-IAD [172]	NVIDIA A100 GPU ...	4	9216 points per ...	✓	–	✓	–	end-to-end	–	24.63 (FPS)	–	465.68 (MB)
CMSA [124]	–	–	224 x 224	–	–	–	–	–	–	36.807 fps	–	157.23 MB
CMT [171]	4 Titan RTX GPUs	8	256x256 (random ...	–	–	–	–	–	–	–	–	–
CoGeoAD [185]	NVIDIA RTX 3090 GPU ...	–	336 x 336; 9 views	–	–	–	–	–	–	1.28 FPS	–	4445MB (GPU ...
CoGeoAD [185]	NVIDIA RTX 3090 GPU ...	–	336 x 336 and 224 x ...	–	–	–	–	–	–	2.70-0.98 FPS ...	–	–
DAS3D [37]	–	–	256x256 depth and ...	–	–	–	–	–	0.041 (inferen ...	–	–	3936 MB
DBRN [100]	NVIDIA GeForce RTX ...	–	–	–	–	–	–	–	–	48.93 (FPS)	–	–
DCMUNet [99]	–	–	256x256	–	–	–	–	–	–	–	32.8 M	–
DCRDF-Net [109]	NVIDIA RTX 2080 (12 ...	1	256x256	–	–	–	–	–	–	about 3 FPS	–	around 3.2 ...
DGCF [125]	NVIDIA RTX 3090	1	256 x 256	–	–	–	–	–	114.1 +/- 0.7 ...	8.8 +/- 0.1 ...	528.8 M	2222.9 MB ...
DRAIN [103]	NVIDIA GeForce GTX ...	4	image size 256 x 256	–	–	–	–	–	–	86.60 FPS	–	–
DUS-Net [102]	RTX3090 (24GB)	–	G = 8192, K = 640 ...	–	–	–	–	–	–	2.1 FPS	–	–
EasyNet [91]	Tesla V100	–	256 × 256	–	–	–	–	–	–	94.55 FPS	–	–
FMFR [126]	NVIDIA GeForce RTX ...	1	224x224	–	–	–	–	end-to-end	–	5.15 fps	–	627.9 MB ...
G2SF-MIAD [179]	Nvidia RTX 4090	–	–	–	–	–	–	–	–	6.04 frames/sa ...	–	–
GLFM [79]	GPU RTX A6000	–	–	✓	–	✓	✓	end-to-end	1.38 (s/per)	–	–	–
GMANet [153]	Xeon 4110 processor ...	–	–	–	–	–	–	–	–	1.34 FPS	–	2.79 GB
GS-CLIP [62]	single NVIDIA RTX ...	–	336x336; 9-view 2D ...	–	–	–	–	model-only ...	0.51 s	1.96 FPS	–	5872 MB
HFMM-Net [132]	same hardware/software ...	–	224 x 224	–	–	✓	✓	end-to-end	40.3 ms	24.80 FPS	–	594.70 MB ...
HFMM-Net [132]	same hardware/software ...	–	224 x 224	–	–	✓	✓	end-to-end	38.5 ms	26.1 FPS	–	524.3 MB ...
HFMM-Net [132]	same hardware/software ...	–	224 x 224	–	–	✓	✓	end-to-end	188.7 ms	5.3 FPS	–	780.2 MB ...
HGAD [33]	–	–	256 x 256	–	–	–	–	–	–	24.3fps	30.8M	–
hoang2026efficient [83]	NVIDIA GeForce RTX ...	–	voxel-grid 0.001 m ...	✓	–	✓	×	end-to-end	–	13.52 FPS	–	–
hoang2026efficient [83]	NVIDIA GeForce RTX ...	–	voxel-grid 0.0005 ...	✓	–	✓	×	end-to-end	–	13.52 FPS	–	–
ISMP [135]	RTX 3090 (24GB)	–	–	–	–	–	–	–	–	2.78 FPS ...	–	–
ISMP [135]	RTX 3090 (24GB)	–	–	–	–	–	–	–	–	4.37 FPS ...	–	–
LCN [80]	single NVIDIA A100 ...	16	N = 2048 points	–	–	–	–	–	–	–	–	–
li2026textguided [175]	–	–	224 x 224 RGB; 1024 ...	–	–	–	–	–	–	10.1 FPS	–	1089.8 MB
LM3M [130]	NVIDIA RTX 3090 GPU ...	1	–	–	–	✓	✓	model-only	13.05 s	–	–	–
LM3M [130]	NVIDIA RTX 3090 GPU ...	1	–	–	–	✓	✓	model-only	9.33 s	–	–	–
LM3M [130]	NVIDIA RTX 3090 GPU ...	1	–	–	–	✓	✓	model-only	12.03 s	–	–	–
LM3M [130]	NVIDIA RTX 3090 GPU ...	1	–	–	–	✓	✓	model-only	10.43 s	–	–	–
LM3M [130]	NVIDIA RTX 3090 GPU ...	1	–	–	–	✓	–	model-only	9.39 s -> ...	–	–	–

Method	Hardware	Batch	Points / res.	P	R	F	M	Scope	Latency	Throughput	Params	Memory
LM3M [130]	NVIDIA RTX 3090 GPU . . .	1	–	–	–	✓	–	model-only	5.53 s -> . . .	–	–	–
LPFSTNet [108]	NVIDIA GeForce RTX . . .	–	768 × 768	–	–	–	–	–	–	38.5 FPS	38.1 (M)	–
M3DM [22]	Nvidia Tesla V100	–	224x224	–	–	–	–	–	–	–	–	at most 50 . . .
MAESTRO [101]	NVIDIA RTX 4070 Ti . . .	–	8192 points	–	–	✓	–	–	195ms	–	–	2GB
MAESTRO [101]	NVIDIA RTX 4070 Ti . . .	–	Multi (16+32+64) . . .	–	–	✓	–	model-only	13.05 ms	–	–	–
MambaAD3D [89]	RTX 3090	1	2048 input points	✓	–	✓	–	end-to-end	41.9 ms	23.9 FPS	–	2.7 GB
MambaAD3D [89]	RTX 3090	1	4096 input points	✓	–	✓	–	end-to-end	57.8 ms	17.3 FPS	–	3.2 GB
MambaAD3D [89]	RTX 3090	1	8192 input points	✓	–	✓	–	end-to-end	79.4 ms	12.6 FPS	–	4.1 GB
MambaAD3D [89]	RTX 3090	1	16,384 input points	✓	–	✓	–	end-to-end	109.6 ms	9.1 FPS	–	5.5 GB
MambaAD3D [89]	RTX-3090 workstation	–	–	✓	–	✓	–	end-to-end	–	17.31 FPS	–	–
MambaAD3D [89]	–	–	–	✓	–	✓	–	end-to-end	–	17.3 FPS	–	3.2 GB; 120 . . .
MC4AD [116]	NVIDIA RTX3090 and 12 . . .	32	All samples were . . .	–	–	–	–	–	–	25.0 (inferenc . . .	14 M	1.3 (inferen . . .
MC4AD [116]	NVIDIA RTX3090 and 12 . . .	32	All samples were . . .	–	–	–	–	–	–	28.1 (inferenc . . .	14 M	2.1 (inferen . . .
MC4AD [116]	NVIDIA RTX3090 and 12 . . .	32	All samples were . . .	–	–	–	–	–	–	50.8 (inferenc . . .	14 M	1.9 (inferen . . .
MC4AD [116]	NVIDIA RTX3090 and 12 . . .	32	All samples were . . .	–	–	–	–	–	–	14.8 FPS	14 M	2.9 (inferen . . .
MCL-AD [64]	NVIDIA RTX 4090 24GB	–	336 x 336	–	–	–	–	–	–	–	–	–
MFEM [156]	Xeon 4110 processor . . .	–	–	–	–	–	–	–	–	–	–	–
MISDD [47]	NVIDIA GeForce RTX . . .	–	–	–	–	–	–	–	–	–	120.43M . . .	–
MMRD [53]	–	–	256x256	–	–	–	–	–	–	10.2 FPS	–	–
MP3D [88]	NVIDIA RTX3090	–	each sample resized . . .	–	–	–	–	–	–	–	–	–
MTFI (cross-modal distillation) [178]	NVIDIA RTX A6000	–	1024 groups x 128 . . .	–	–	✓	✓	–	1.74 s	–	–	–
MTFI (cross-modal distillation) [178]	NVIDIA RTX A6000	–	1024 groups x 128 . . .	–	–	–	✓	–	1.02 s - 2.90 . . .	–	–	coreset . . .
MTFI (cross-modal distillation) [178]	–	–	RGB (224,224,3)	–	–	–	–	model-only	–	–	RGB featur . . .	–
MTFI (cross-modal distillation) [178]	NVIDIA RTX A6000 + . . .	–	–	–	–	–	–	end-to-end	–	–	–	maximum 50 . . .
MulSen-TripleAD [87]	a Tesla V100 GPU	–	224 x 224 images . . .	–	–	–	–	–	–	–	–	–
MVP-PCLIP [61]	GPU RTX A6000	–	224-pixel input . . .	–	–	–	–	–	–	5.1 fps and . . .	–	–
OmniposeAD [142]	–	4096	400x400 (MAD-Sim) . . .	–	–	–	–	–	–	–	–	–
OmniposeAD [142]	single NVIDIA Tesla . . .	3072	–	–	–	–	–	end-to-end	–	–	–	–
Open3D-AD (M1 Correspondence Distributions Subsampling) [154]	NVIDIA RTX 4090 GPU . . .	–	N = 1000 target . . .	–	–	✓	✓	–	–	8.4	–	–

Method	Hardware	Batch	Points / res.	P	R	F	M	Scope	Latency	Throughput	Params	Memory
Patch3D [82]	two RTX 2080Ti	–	–	–	–	–	–	–	–	–	–	–
PCDiff [184]	–	–	–	×	–	–	–	–	2.92 s	–	–	–
PCDiff [184]	–	–	–	–	–	–	–	–	–	–	–	17,312 MB . . .
PCDiff [184]	–	–	–	✓	–	✓	–	–	1.35 s	–	–	–
PLANE [181]	single NVIDIA RTX . . .	–	2048 points	–	–	–	–	–	–	FPS of 1.53	–	–
PointAD [20]	NVIDIA RTX 3090 24GB	1	9 rendered views at . . .	–	–	–	–	–	0.40 s	2.52 FPS	–	4275MB
PointAD [63]	NVIDIA RTX 3090 24GB . . .	1	336 x 336 renderings . . .	–	–	✓	–	–	–	2.52 FPS	1656.34 . . .	4275MB GPU . . .
PointAD+ [63]	NVIDIA RTX 3090 24GB . . .	1	336 x 336 renderings . . .	–	–	✓	–	–	–	2.18 FPS	1658.34 . . .	5811MB GPU . . .
PointCore [71]	12th Gen Intel Core . . .	–	greedy down-sampling . . .	–	✓	–	✓	–	4.282 s	–	–	–
proposed pipeline (SplatPose+ with BiRefNet and LaMa) [176]	NVIDIA RTX-A4500 GPU	–	800 x 800	✓	✓	✓	–	end-to-end	1.7 +/- 0.0 s . . .	–	–	–
proposed pipeline without BiRefNet and LaMa [176]	NVIDIA RTX-A4500 GPU	–	800 x 800	×	✓	✓	–	end-to-end	0.9 +/- 0.0 s	–	–	–
R^3-PAD [117]	single NVIDIA RTX . . .	–	448x448 (Retinex-UNe . . .	✓	✓	✓	–	end-to-end	70.7 ms	FPS from 10 . . .	–	–
R3D-AD [36]	NVIDIA RTX 3090	–	4096 points	–	–	–	–	–	–	–	–	–
ReflectSL [148]	–	–	–	✓	–	–	–	–	390 ms	–	–	–
ReflectSL [148]	–	–	–	✓	–	–	–	–	several . . .	–	–	–
Reg2Inv [77]	RTX 3090 GPU	1	–	–	–	–	–	–	–	–	–	–
Reg2Inv [77]	RTX 3090 GPU	1	8,192 sampled . . .	–	✓	✓	✓	–	2.53 seconds	–	–	–
Reg3D-AD [19]	–	–	point groups 16384	–	–	–	–	–	–	–	–	memory bank . . .
RIF (CTF-Net) [78]	RTX3090 (24GB)	–	G = 512 groups, K = . . .	–	–	–	–	–	–	0.31 FPS	4.1M	6.20 GB . . .
SeDiR [96]	NVIDIA GeForce RTX . . .	–	1024 groups via FPS	–	–	–	–	–	–	–	33.25M	–
Shape-Guided [46]	Nvidia GTX 1080	–	224x224 images . . .	–	–	✓	✓	–	2.05	0.69 FPS	–	13.5% RGB . . .
Shape-Guided [46]	–	–	–	–	–	–	–	–	–	–	3.17M . . .	–
Simple3D [81]	NVIDIA A100	–	4k points (sampling . . .	–	–	–	–	–	–	exceeding 20 . . .	–	–
SplatPose [58]	a single NVIDIA RTX . . .	–	400 x 400 pixels	–	✓	✓	–	model-only	00:05±00 . . .	–	–	–
SplatPose [58]	a single NVIDIA RTX . . .	–	400 x 400 pixels	–	✓	✓	–	model-only	roughly one . . .	–	–	–
SplatPose+ [150]	single NVIDIA RTX 3090	–	–	–	✓	✓	–	end-to-end	34 millisecond . . .	approximately . . .	–	–
SplatPose+ [150]	single NVIDIA RTX 3090	–	–	✓	✓	–	–	end-to-end . . .	184 seconds . . .	–	–	–
Template3D-AD [56]	NVIDIA RTX 3090	–	4096 center points	–	–	–	–	–	–	–	–	–
TopoOT [169]	NVIDIA RTX 5090 GPU . . .	1	–	✓	–	✓	–	end-to-end	–	approximately . . .	–	349 MB
TopoOT [169]	NVIDIA RTX 5090 GPU . . .	1	224 x 224 (2D . . .	×	–	×	–	model-only	–	121 FPS	–	349 MB
TopoOT [169]	NVIDIA RTX 5090 GPU . . .	1	–	×	–	×	–	model-only	PD 0.325-0.397 . . .	–	–	0.349-0.495 . . .
TRD [107]	single Nvidia GTX 3090	16	256 x 256	×	–	✓	–	model-only	–	50.8 FPS	524.5 (M)	–
TRD [107]	single Nvidia GTX 3090	16	256 x 256	✓	–	✓	–	end-to-end	–	31.5 FPS	524.5 (M)	–

Method	Hardware	Batch	Points / res.	P	R	F	M	Scope	Latency	Throughput	Params	Memory
UCF [128]	NVIDIA GeForce RTX ...	–	–	–	–	–	–	–	–	5.147 FPS	–	585.6 MB
UCF [128]	–	–	56×56 feature-map ...	–	–	–	–	–	–	–	–	–
UniMF [129]	NVIDIA GeForce RTX ...	–	image size 224	–	–	–	–	–	146 ms	–	–	–
V2G (Ours, -GCN) [98]	NVIDIA GeForce RTX ...	–	224x224 images ...	–	–	–	–	–	–	8.801 FPS	–	1002 MB
V2G (Ours, -MFE) [98]	NVIDIA GeForce RTX ...	–	224x224 images ...	–	–	–	–	–	–	9.814 FPS	–	942 MB
V2G (Ours, -NLA) [98]	NVIDIA GeForce RTX ...	–	224x224 images ...	–	–	–	–	–	–	8.762 FPS	–	985 MB
V2G (Ours, Full) [98]	NVIDIA GeForce RTX ...	–	224x224 images ...	–	–	–	–	–	–	8.213 FPS	–	1045 MB
Vote3D-AD [173]	four NVIDIA RTX-3090 ...	–	scans downsampled ...	×	–	✓	–	model-only	–	9.05 FPS	18.6M	–
Vote3D-AD (Small-Transformer variant) [173]	four NVIDIA RTX-3090 ...	–	50,000 points	×	–	✓	–	model-only	–	13.3 FPS	8.9M	–
ZUMA [121]	NVIDIA RTX 3090	–	9 rendered views ...	–	–	–	–	–	–	–	0.00M	–
ZUMA-FT [121]	NVIDIA RTX 3090	4	9 rendered views ...	–	–	–	–	–	–	–	6.75M	–

S14. Experiments That Vary a Sensing Condition

Table S12. Every experiment in the corpus that varies a sensing condition and reports the effect on detection. Effects are quoted in the units and metric each source uses and are not converted or compared across rows, since each comes from a different pipeline. Rows are grouped by condition in the order Section 11.5 discusses them.

Condition	Source	Benchmark	What was varied	Reported effect
Additive noise	3DSR/DADA [48]	MVTec3D	depth-simulation generator used to pretrain DADA: Perlin noise maps replaced with grayscale ImageNet images (no-perlin), and Perlin map used without the alpha/beta affine scaling (no-affine)	full 3DSR 97.8 / 99.5 / 97.2; 3DSR no-affine 94.8 / 99.2 / 95.9 ('approximately 3 percentage point drop' in image AUROC); 3DSR no-perlin 90.0 / 98.3 / 93.3 ('by approximately 8 percentage points' in image AUROC)
Additive noise	ActiveInspect [147]	Real-IAD D3	additive Gaussian noise on RGB (sigma = 0/8/16/32 on the 0-255 scale) and depth noise before rendering (sigma = 0/0.5/1/2 mm)	largest degradation across all corruption types is 0.023 I-AUROC, under severe depth noise; per-severity values are plotted only
Additive noise	DUS-Net [102]	AnomalyShapeNet	Gaussian noise parameters alpha (group center) and beta (patch) in Noise-Gen; and the noise family, Gaussian replaced by uniform or salt-and-pepper	O-AUROC 0.767 to 0.796 across six (alpha/beta) settings, best 0.797 at (0.08/0.15); replacing Gaussian with uniform gives O-AUROC 0.572 and with salt-and-pepper 0.642
Additive noise	Eyecandies [141]	Eyecandies	sensor noise deliberately absent	"no noise is intentionally added to the data"; "we do not model any real acquisition device"
Additive noise	LDSD [105]	Real3D	Gaussian noise with standard deviation 0, 0.001, 0.003, 0.005 or 0.01 injected into test samples	O-AUROC 0.854 (sd 0.001), 0.856 (0.003), 0.853 (0.005), 0.857 (0.01); P-AUROC 0.841, 0.844, 0.843, 0.84 respectively
Additive noise	M3DM-NR [123]	MVTec3D	proportion of anomalous samples injected from the test set into the training set: 10%, 20%, 30% (Overlap and Non-Overlap settings)	3D+RGB Overlap I-AUROC 86.6 +/- 1.3 at 10% noise, 79.3 +/- 1.0 at 20%, 76.8 +/- 0.6 at 30% (M3DM: 66.3, 42.9, 27.9 over the same levels)
Additive noise	MC4AD [116]	Anomaly-IntraVariance	Group 2 of the authors' Anomaly-IntraVariance dataset has fewer points and added Gaussian noise (variance 0.002) relative to Group 1	O-AUROC 0.761 (Group 1) -> 0.625 (Group 2); P-AUROC 0.807 -> 0.678
Additive noise	PO3AD [59]	AnomalyShapeNet	Gaussian noise added to test point clouds with standard deviation 0, 0.001, 0.003, 0.005 (0 = clean)	performance drops only slightly from std 0 to 0.005 on bottle0, bottle1, bottle3; worst case still above R3D-AD on clean data (73.3%, 73.7%, 78.1% object-level AUC-ROC)
Additive noise	PointAD+ [63]	MVTec3D	rendering quality: Gaussian blur applied to the 2D renderings at sigma 0, 1, 5, 9 to simulate the noise level	local (96.9, 88.7) / global (82.5, 94.8) at sigma=0; (96.8, 88.1) / (81.3, 93.9) at sigma=1; (96.6, 87.4) / (77.7, 92.8) at sigma=5; (96.7, 87.1) / (77.5, 92.3) at sigma=9
Additive noise	PointCore [71]	Real3D	registration error tests on point-point ICP and point-plane ICP under different levels of Gaussian noise	point-plane ICP yields more accurate registration at lower noise levels; no per-noise-level numbers printed
Additive noise	Reg2Inv [77]	Real3D	Gaussian noise added to test point clouds only, no noise augmentation during training (Setting 2)	O-AUROC 78.0 / P-AUROC 87.8 clean -> 76.9/86.5 at SD=0.001 -> 63.0/69.1 at SD=0.003 -> 56.6/56.5 at SD=0.005
Additive noise	Reg2Inv [77]	Real3D	Gaussian noise (SD = 0.005) injected during training, varying test-time noise levels (Setting 1)	O-AUROC 78.0 / P-AUROC 87.8 clean -> 76.7/87.5 at SD=0.001 -> 77.7/86.4 at SD=0.003 -> 76.8/83.1 at SD=0.005
Additive noise	RIF (CTF-Net) [78]	Real3D-AD	Gaussian noise added to test point clouds at standard deviation 0.002	P-AUROC 0.768 average, O-AUROC 0.583 average under sigma=0.002 (one-shot)

Condition	Source	Benchmark	What was varied	Reported effect
Additive noise	RIF (CTF-Net) [78]	Real3D-AD	Gaussian noise added to test point clouds at standard deviation 0.003	P-AUROC 0.702 average, O-AUROC 0.569 average under sigma=0.003 (one-shot); down from 0.768/0.583 at sigma=0.002
Point density or downsampling	3D-ST [51]	MVTec3D	number of input points n after farthest point sampling (16k, 32k, 64k, 128k)	AU-PRO 0.759 at 16k -> 0.803 at 32k -> 0.818 at 64k -> 0.821 at 128k points
Point density or downsampling	ActiveInspect [147]	Real-IAD D3	random point-cloud downsampling to 100 / 75 / 50 / 25% of the points	per-level values plotted only in Fig. 8b; the only printed figure for this corruption family is the 0.023 I-AUROC maximum degradation, which the text attributes to depth noise rather than downsampling
Point density or downsampling	AEMG [85]	MVTec3D	3D local patch size K, i.e. the number of points per point-cloud patch: 700, 600, 500, 400, 300	I-AUROC 0.939 (K=700), 0.946 (600), 0.956 (500), 0.956 (400), 0.961 (300); AUPRO@30% 0.971/0.973/0.976/0.975/0.975; AUPRO@1% 0.453/0.456/0.461/0.456/0.458; inference time 1.36/1.42/1.58/1.74/2.06
Point density or downsampling	BTP [66]	Real3D-AD	number of input points after farthest point sampling: 1024, 2048, 4096, 8192	O-AUROC 62.7 (1024) / 61.4 (2048) / 62.9 (4096) / 61.5 (8192); P-AUROC 83.0 / 84.5 / 82.3 / 80.1; P-AUPRO 52.9 / 55.5 / 54.1 / 53.7
Point density or downsampling	C3D-AD [104]	AnomalyShapeNet	number of point groups (#Groups) 512, 1024, 2048, 4096, 8192	AUROC plotted against #Groups from 512 to 8192; the paper states 4096 gives satisfactory performance. Inference time and GPU peak memory increase linearly with #Groups
Point density or downsampling	CDRS [131]	MVTec3D	point grouping of the released-code baseline: group size 128 with 1024 groups versus group size 64 with 784 groups plus multiscale	0.779 / 0.975 / 0.915 (group 128, num 1024) -> 0.781 / 0.976 / 0.917 (group 64, num 784, multiscale)
Point density or downsampling	DUS-Net [102]	AnomalyShapeNet	number of groups G (1024, 4096, 8192) and number of points per group K (128, 384, 640)	O-AUROC 0.618 (G=1024) to 0.707 (4096) to 0.797 (8192) at K=640; O-AUROC 0.685 (K=128), 0.724 (K=384), 0.797 (K=640) at G=8192; FPS 3.4 to 2.1
Point density or downsampling	Hierarchical Asymmetric Student-Teacher [180]	MVTec3D	input point cloud size reduced from 8192 points (default) down to 2048 points	at 2048 points the model 'maintains a certain level of anomaly detection efficacy'; per-size values are plotted only, not printed
Point density or downsampling	IMRNet [49]	–	point-cloud downsampling method used to pick the 256 patch centres: random sampling (RS), farthest point sampling (FPS), voxel down-sampling, geometry-aware sampling (GPS)	I-AUC 0.55 (RS) / 0.64 (FPS) / 0.62 (Voxel) / 0.66 (GPS); P-AUC 0.61 (RS) / 0.62 (FPS) / 0.55 (Voxel) / 0.65 (GPS)
Point density or downsampling	M3DM [22]	MVTec3D (pure 3D setting)	Point Transformer grouping (points per group S.G / number of groups N.G: 64/784 vs 128/1024) and 2D pooling patch size (point groups, 28×28, 56×56)	I-AUROC 0.793 (64/784, point group) / 0.841 (128/1024, point group) / 0.805 (64/784, 28×28) / 0.819 (128/1024, 28×28) / 0.874 (128/1024, 56×56); AUPRO 0.813 / 0.896 / 0.879 / 0.896 / 0.906; P-AUROC 0.922 / 0.960 / 0.963 / 0.967 / 0.970
Point density or downsampling	M3DM-NR [123]	MVTec3D	threshold theta, the minimum number of points required for a point-cloud patch to be considered meaningful: 128, 256, 512, 1024	Overlap I-AUROC 86.6 +/- 1.3 (theta=128), 86.2 +/- 0.6 (256), 85.9 +/- 0.4 (512), 84.0 +/- 0.5 (1024); Overlap AUPRO 90.7 / 90.3 / 90.2 / 89.0
Point density or downsampling	MAD (PAD benchmark) [142]	MAD-Real	training-view density on MAD-Real (sample size 100 vs 70 vs 50)	per class at 100/70/50, e.g. Gorilla image 93.6/85.4/91.0 pixel 99.5/97.5/97.4; Zalika image 88.2/78.8/72.8; Mallard image 87.3/80.3/74.6
Point density or downsampling	MambaAD3D [89]	–	input point count per sample: 2048, 4096, 8192, 16,384	no detection metric reported; total latency 41.9 -> 57.8 -> 79.4 -> 109.6 ms, FPS 23.9 -> 17.3 -> 12.6 -> 9.1, peak memory 2.7 -> 3.2 -> 4.1 -> 5.5 GB

Condition	Source	Benchmark	What was varied	Reported effect
Point density or downsampling	MP3D [88]	self-collected vehicle stamped parts dataset	number of input center points (1024 / 2048 / 4096)	MP3D I-AUROC 89.7 -> 90.5 -> 91.8 and P-AUROC 91.3 -> 92.1 -> 94.3 for 1024 -> 2048 -> 4096 center points
Point density or downsampling	PointAD [20]	MVTec3D	train on the original high-density clouds, test on downsampled versions (70%, 50%, 30%, 20%): a density gap between train and test	point global (82.0, 94.2) at 100% -> (81.3, 94.0) 70% -> (79.6, 93.4) 50% -> (76.6, 91.8) 30% -> (72.7, 90.5) 20%; point local (95.5, 84.4) -> (94.2, 78.2) at 20%
Point density or downsampling	PointAD+ [63]	MVTec3D	input point density: original point clouds downsampled with Farthest Point Sampling at ratios 70%, 50%, 30%, 20%	local (96.9, 88.7) and global (82.5, 94.8) at 100% -> local (96.2, 86.1) / global (82.8, 94.5) at 70%, (95.8, 85.1) / (81.2, 93.8) at 50%, (95.7, 84.3) / (79.0, 92.8) at 30%, (95.8, 83.5) / (75.0, 91.5) at 20%
Point density or downsampling	Real-IAD D3 [152]	Real-IAD D3	point cloud downsampled 4x (PointMAE)	e.g. connector housing-female Hole I-AUROC 0.516 -> 0.500 P 0.819 -> 0.801; Deformation 0.872 -> 0.804 / 0.653 -> 0.638; Pit 1.000 -> 0.976 / 0.457 -> 0.447; headphone jack socket Pit P 0.905 -> 0.832; audio jack socket Scratch P 0.919 -> 0.802
Point density or downsampling	Reg3D-AD [19]	Real3D	number of point groups fed to the Point Transformer / PointMAE feature extractor: 16384 vs 8192 (memory size held at 10000)	O-AUROC 0.705 (16384) -> 0.666 (8192); P-AUROC 0.700 -> 0.681; O-AUPR 0.723 -> 0.653; P-AUPR 0.113 -> 0.120
Point density or downsampling	SeDiR [96]	Real3D	number of FPS group centres partitioning the point cloud (512 / 1024 / 2048)	O-AUROC 75.7 (512) / 81.0 (1024) / 77.7 (2048); P-AUROC 77.4 / 80.6 / 78.4
Point density or downsampling	SiM3D [146]	SiM3D	downsampling required to run Point-MAE (~5-7M to ~8k points)	defects "no longer show up in the point clouds" at ~8k points
Point density or downsampling	SiM3D [146]	SiM3D	point count of SiM3D vs existing multimodal benchmarks	~8K vs ~2.3M points (after downsampling); "current multimodal ADS methods do not scale to significantly higher resolutions"
Point density or downsampling	SplatPose+ [150]	MAD-Sim	fraction of the 210 reference views used for training (20%, 40%, 60%, 80%, 100%)	no numeric values printed; paper states SplatPose+ has the highest AUROC and AUPRO at every sparse-view level, with the largest margin at 20%
Point density or downsampling	Teacher-Student Network [177]	ShapeNet-Part	number of sampled points per point cloud (512 / 1024 / 2048) used for both training and test	more points generally raise AUC but 'the change of detection accuracy does not vary greatly'; per-setting AUC values not printed (ROC curves only)
Point density or downsampling	Template3D-AD [56]	Real3D	number of FPS center points sampled from the registered test cloud: 512, 1024, 2048, 4096	I-AU 0.816 / 0.833 / 0.840 / 0.844 and P-AU 0.935 / 0.938 / 0.933 / 0.925 at 512 / 1024 / 2048 / 4096 center points
Point density or downsampling	Vote3D-AD [173]	Anomaly-ShapeNet and Industry3D Anomaly Benchmark (averaged)	uniform subsampling of input points to 40k, 30k, 20k, 10k, 5k from the standardized 50k-point input	Point AUROC 90.3 (50,000) -> 89.9 (40k) -> 84.5 (30k) -> 77.1 (20k) -> 67.3 (10k) -> 55.0 (5k); Object AUROC 85.5 -> 84.8 -> 82.3 -> 80.0 -> 77.6 -> 73.7
Point density or downsampling	ZUMA [121]	MVTec3D	point-cloud sampling ratio 100%, 80%, 50%, 20%	P-AUC 97.6 at 100% -> 97.5 (-0.1) at 80%, 97.3 (-0.3) at 50%, 96.3 (-1.3) at 20%; I-AUC 84.0 -> 83.7 (-0.3), 82.9 (-1.1), 81.9 (-2.1)

Condition	Source	Benchmark	What was varied	Reported effect
Missing points, holes, dropout	BTF [23]	MVTec3D	3D-aware preprocessing applied or not: RANSAC background-plane removal plus connected-components outlier removal, motivated by how depth and point-cloud methods handle missing sensor values	raw -> preprocessed: HoG PRO 0.625 -> 0.771 and I-ROC 0.558 -> 0.559; SIFT PRO 0.869 -> 0.910 and I-ROC 0.723 -> 0.727; FPFH PRO 0.930 -> 0.924 and I-ROC 0.764 -> 0.782; RGB PRO 0.876 -> 0.876 and I-ROC 0.788 -> 0.770
Missing points, holes, dropout	EasyNet [91]	Eyecandies	depth modality removed entirely (simulated depth-sensor failure; RGB-only input)	I-AUROC 0.869 (RGB+Depth) -> 0.858 (Pure RGB)
Missing points, holes, dropout	EasyNet [91]	MVTec3D	depth modality removed entirely (simulated depth-sensor failure; RGB-only input)	I-AUROC 0.926 (RGB+Depth) -> 0.904 (Pure RGB)
Missing points, holes, dropout	FMFR [126]	MVTec3D	random dropout of 10%, 30%, 50% of valid foreground points (coordinates set to zero) at test time, no retraining	I-AUROC 98.0 (0%) -> 94.1 (10%) -> 90.5 (30%) -> 75.7 (50%); AUPRO@30% 97.9 -> 95.6 -> 92.4 -> 80.1
Missing points, holes, dropout	MambaAD3D [89]	Real3D	visibility / occlusion level of the test object: low (<10%), moderate (10-30%), heavy (30-50%), severe (>50%)	AUROC 83.1 -> 79.0 -> 74.8 -> 68.5 and AUPR 28.7 -> 22.6 -> 17.0 -> 11.0 from low to severe occlusion
Missing points, holes, dropout	MISDD [47]	MVTec 3D-AD	RGB modality missing at rate eta = 30%, 50%, 70% (simulating unavailable RGB sensor)	I-AUROC 78.80 -> 73.86 -> 68.75; P-AUROC 93.38 -> 90.78 -> 89.22; AUPRO 79.20 -> 70.50 -> 65.31 at eta = 30/50/70%
Missing points, holes, dropout	MISDD [47]	MVTec 3D-AD	3D modality missing at rate eta = 30%, 50%, 70% (simulating unavailable 3D sensor)	I-AUROC 81.97 -> 81.74 -> 81.82; P-AUROC 98.16 -> 98.10 -> 97.98; AUPRO 92.68 -> 92.44 -> 91.99 at eta = 30/50/70%
Missing points, holes, dropout	MISDD [47]	MVTec 3D-AD	both RGB and 3D missing, each at rate eta/2, for total eta = 30%, 50%, 70%	I-AUROC 77.71 -> 76.95 -> 73.83; P-AUROC 95.00 -> 93.28 -> 93.05; AUPRO 84.03 -> 79.79 -> 77.44 at eta = 30/50/70%
Missing points, holes, dropout	MTFI (cross-modal distillation) [178]	MVTec3D	modality availability at inference: complete RGB+PCs dual memory bank vs. PCs-only vs. RGB-only	PCs-only dual bank vs complete RGB+PCs bank: I-AUROC 7.8% down and AUPRO 5.1% down (0.860 vs 0.938 I-AUROC); MTFI F2F with PCs-only inference restores I-AUROC 0.938 / AUPRO 0.934
Missing points, holes, dropout	PointAD [20]	MVTec3D	point clouds occluded by removing points not visible from a chosen rendering angle, then re-rendered with the same parameters	point global (82.0, 94.2) -> (73.3, 90.6); point local (95.5, 84.4) -> (94.3, 80.8); multimodal global (86.9, 96.1) -> (83.0, 94.8); multimodal local (97.2, 90.2) -> (96.7, 89.5)
Missing points, holes, dropout	PointAD+ [63]	MVTec3D	point occlusion: points invisible from a given rendering angle removed and the rest projected, at angles chosen to keep anomalous regions visible	local (96.9, 88.7) / global (82.5, 94.8) original -> local (95.4, 83.9) / global (73.6, 90.6) occluded
Missing points, holes, dropout	Vote3D-AD [173]	Anomaly-ShapeNet and Industry3D Anomaly Benchmark (averaged)	contiguous occlusion: points removed inside a single axis-aligned box of 10%, 25%, 50%, 75% of the object bounding-box volume	Point AUROC 90.3 (0%) -> 85.1 (10%) -> 70.4 (25%) -> 50.1 (50%) -> 30.2 (75%); Object AUROC 85.5 -> 80.6 -> 66.0 -> 55.5 -> 40.0
Missing points, holes, dropout	ZUMA [121]	Eyecandies	modality dropout: missing RGB only, missing point cloud only, or both, at missing rates eta = 5%, 10%, 30%, 50%	multimodal mean 97.3 / 88.9 / 74.9 with no missing modalities -> 96.5 / 86.9 / 74.6 at 5%, 96.1 / 85.1 / 72.5 at 10%, 92.2 / 76.0 / 69.1 at 30%, 88.0 / 70.1 / 68.0 at 50%

Condition	Source	Benchmark	What was varied	Reported effect
Missing points, holes, dropout	ZUMA [121]	MVTec3D	modality dropout: missing RGB only, missing point cloud only, or both, at missing rates eta = 5%, 10%, 30%, 50%	multimodal mean 97.6 / 92.5 / 84.0 with no missing modalities -> 97.3 / 91.6 / 83.6 at 5%, 96.7 / 90.2 / 81.4 at 10%, 96.0 / 87.4 / 77.3 at 30%, 94.9 / 84.7 / 75.1 at 50%
Missing points, holes, dropout	ZUMA [121]	MVTec3D	occlusion applied to the input	P-AUC 97.6 -> 97.3 (-0.3); I-AUC 84.0 -> 83.2 (-0.8)
Calibration or modality misalignment	ActiveInspect [147]	Real-IAD D3	test-time extrinsic rotation error up to 2 degrees and focal-length error up to +/-2%, with all geometric observations re-rendered and no policy retraining	I-AUROC 0.906 -> 0.892 at 2 degrees; Geo-AUROC 0.895 -> 0.874; focal-length errors of up to +/-2% change I-AUROC by at most 0.004
Calibration or modality misalignment	IEC3D-AD [153]	IEC3D-AD	calibration artefact and procedure	5mm alumina calibration plate, moved several times over the whole camera view field
Calibration or modality misalignment	PatchCore (FPFH+Raw), Reg3D-AD, ISMP (evaluated in this paper) [82]	Real3D-AD	registration accuracy, varied by modifying the voxel size in the RANSAC alignment algorithm	positive correlation for all three methods; Supp. Tables 5-6 p. 17 (same PDF): mean P-AUROC at registration error R 0.80 -> 0.30: Reg3D-AD 0.646 -> 0.707, PatchCore (FPFH+Raw) 0.646 -> 0.721, ISMP 0.736 -> 0.842; mean P-AUPR 0.048 -> 0.118, 0.052 -> 0.1395, 0.153 -> 0.181; the 12 Real3D-AD categories only
Calibration or modality misalignment	PD-REAL [151]	PD-REAL	camera-to-background alignment before capture	leveller used so the background is parallel to the camera plane "to reduce the depth error"
Calibration or modality misalignment	Real-IAD D3 [152]	Real-IAD D3	RGB and 3D captured on one platform	"natural alignment without additional calibration"
Calibration or modality misalignment	SiM3D [146]	SiM3D	calibration-pattern accuracy (occurrence 1 of 2)	"centre coordinates with precision < 1 mm"
Calibration or modality misalignment	SiM3D [146]	SiM3D	SRF-to-CRF roto-translation estimation accuracy (occurrence 2 of 2)	"a precision under 1 mm"
Change of sensor or capture setup	ActiveInspect [147]	Real-IAD D3	removal of the depth and 3D observation modality from the action pool (w/o depth/3D)	I-AUROC 0.906 -> 0.876 (-0.030) and Geo-AUROC 0.895 -> 0.853; single-image mode (no view or modality selection) 0.846 (-0.060)
Change of sensor or capture setup	APG-Net [86]	MulSen-AD	which of the three MulSen-AD sensor modalities is used (RGB camera / lock-in infrared thermography / laser-scanner point cloud) and their combinations	RGB only 89.8%; infrared only 88.8%; point cloud only 73%; infrared+RGB 94%; all three modalities 97.4%
Change of sensor or capture setup	CMT [171]	BrokenChairs-180K	number of multi-view reference images used at training (Ntrain in {5,10}) and at test (Ntest in {5,10,15,20})	increasing views in both training and testing helps; no numeric values printed in text (values only in Fig. 7 right)
Change of sensor or capture setup	CMT [171]	real chair samples (in-house)	model trained on synthetic renders, evaluated on real chairs photographed while walking around them and reconstructed with 3D reconstruction software plus Laplacian smoothing	5 out of 6 cases correctly classified and localized; the failure case relates a self-occluded arm with an anomaly

Condition	Source	Benchmark	What was varied	Reported effect
Change of sensor or capture setup	DMP-3DAD [182]	ShapeNet-Part	number of rendered depth-map views per point cloud (V = 5 / 10 / 20 / 30) from the fixed 3D view grid	1-shot 90.34 / 92.63 / 92.49 / 91.78; 3-shot 94.50 / 96.07 / 96.08 / 95.33; 5-shot 94.95 / 96.42 / 96.44 / 95.60 for V = 5/10/20/30
Change of sensor or capture setup	DRAIN [103]	MVTec 3D-AD	input modality: depth only vs RGB only vs RGB+depth, explicitly framed as simulating depth-sensor failure	Image-AUROC 82.7 (depth only) / 92.8 (RGB only) / 94.3 (RGB+depth); AUPRO 83.5 / 88.6 / 89.2
Change of sensor or capture setup	GLFM [79]	Actual industry parts (in-house; Blade 1, Blade 2, Impeller, Plastic part)	evaluation moved from public benchmarks to actual industry parts scanned by an industrial robot equipped with 3D sensors	mean 82.4/90.2; Blade 1 100.0/98.2, Blade 2 73.3/92.2, Impeller 58.8/93.9, Plastic part 97.6/76.3
Change of sensor or capture setup	GLFM [79]	Mix of MVTec3D and Real3D	MVTec 3D-AD and Real3D-AD (different sensors and capture setups) mixed into one 22-class dataset	72.9% object-wise AUROC, 93.1% point-wise AUROC, and 77.9% point-wise AUPRO as stated in the prose; Table IV p. 8 prints a GLFM mean of 80.2 / 93.5 / 79.2 for the same setting, so the paper contradicts itself
Change of sensor or capture setup	GLFM [79]	Real3D	Real3D-AD test data reduced to a single view and converted to 2.5D, while training data remain complete multi-view scans	53.2 / 89.8 / 67.0
Change of sensor or capture setup	GS-CLIP [62]	MVTec3D; Real3D; Eyecandies; AnomalyShapeNet	number of rendered views	metrics show an overall upward trend with more views, saturating at approximately 9 views; no numeric values printed in text (values only in Fig. 6)
Change of sensor or capture setup	LPFSTNet [108]	MVTec3D vs own dataset	evaluation dataset and sensor: MVTec3D (Zivid One+ Medium, per paper) vs own dataset (Intel D435 depth camera)	I-AUROC 94.7 (MVTec3D) vs 97.6 (own); P-AUROC 97.2 (MVTec3D) vs 90.6 (own)
Change of sensor or capture setup	MambaAD3D [89]	RealScan-AD vs Real3D	capture setup: RealScan-AD collected with two commodity depth sensors, versus Real3D-AD structured-light scans	point-level AUROC 78.9 on RealScan-AD vs 76.6 on Real3D-AD; point-level AUPR 20.5 vs 19.7; object-level AUROC 80.0 vs 78.4; object-level AUPR 81.6 vs 77.7
Change of sensor or capture setup	MulSen-AD (dataset) / MulSen-TripleAD [87]	MulSen-AD	which sensor(s) feed the detector: RGB, infrared (IR), point cloud (PC), and their pairwise and triple combinations	RGB 0.911; IR 0.909; PC 0.668; PC+RGB 0.919; PC+IR 0.912; RGB+IR 0.948; RGB+IR+PC 0.961
Change of sensor or capture setup	MVP-PCLIP [61]	MVTec3D; Real3D	number of rendered depth views increased from one to nine	P-R, P-F and P-P increase with the number of views, optimal at nine views; FPS drops from 29.9 fps and 27.9 fps to 5.1 fps and 5.9 fps
Change of sensor or capture setup	MVP-PCLIP [61]	Plastic parts inspection (in-house)	evaluation moved from public benchmarks to in-house automotive plastic parts scanned by an industrial robot equipped with 3D sensors	Object-wise (57.4, 68.5, 62.6); Point-wise (93.1, 27.9, 20.2)
Change of sensor or capture setup	PointAD+ [63]	MVTec3D	number of rendering views K varied over 1, 3, 5, 7, 9, 11	local (95.8, 83.7) / global (74.0, 90.7) at K=1; (96.4, 86.4) / (79.2, 92.9) at K=3; (96.8, 87.7) / (81.7, 93.9) at K=5; (96.7, 87.6) / (81.8, 94.2) at K=7; (96.9, 88.7) / (82.5, 94.8) at K=9; (96.6, 87.4) / (82.7, 94.4) at K=11

Condition	Source	Benchmark	What was varied	Reported effect
Change of sensor or capture setup	proposed pipeline (SplatPose+ with BiRefNet and LaMa) [176]	own Blender LEGO-vehicle dataset	test images rendered with realistic cluttered conveyor background vs background-free images	with background clutter (Table 2) p-AUROC 0.992-0.999, AUPRO 0.977-0.997, i-AUROC 0.921-0.993; background-free (Table 7) p-AUROC 0.999 for all nine classes, AUPRO 0.984-0.996, i-AUROC 0.920-0.993
Change of sensor or capture setup	R^3-PAD [117]	–	percentage of available capture views used to build the 3D reference model (20 / 40 / 60 / 80 / 100%)	R3-PAD I-AUROC 90.2 / 94.9 / 96.8 / 97.7 / 97.9 and P-AUPRO 95.6 / 96.4 / 96.8 / 97.0 / 97.2 at 20/40/60/80/100% of views
Change of sensor or capture setup	R3D-AD [36]	Real3D	training data source for the same category: synthetic ShapeNetCore.v2 meshes vs real Real3D-AD scans	Airplane: 0.614 trained on ShapeNetCore.v2 vs 0.772 oracle trained on Real3D-AD; Car: 0.601 vs 0.713
Change of sensor or capture setup	SiM3D [146]	MVTec AD	greyscale vs RGB imagery (sanity check on MVTec AD)	PatchCore I-AUROC 0.988 (greyscale) vs 0.991 (official RGB); AUPRO@30% 0.929 vs 0.935
Change of sensor or capture setup	SiM3D [146]	SiM3D	real training scan vs CAD-rendered synthetic training scan (real2real vs synth2real)	PatchCore I-AUROC 0.754 -> 0.540; AST V-AUPRO@1% 0.699 -> 0.695
Change of sensor or capture setup	SiM3D [146]	SiM3D	single-view capture vs multiview capture	PatchCore mean I-AUROC 0.488 (single-view) vs 0.754 (multiview)
Change of sensor or capture setup	SplatPose [58]	MAD	view sparsification: training on 20%, 40%, 60%, 80%, 100% of the 210 available views per MAD category	SplatPose AUROC 77.6 (20% views) -> 89.3 (40%) -> 92.2 (60%) -> 93.1 (80%) -> 93.9 (100%); AUPRO 86.5 -> 93.3 -> 95.0 -> 95.5 -> 95.8
Change of sensor or capture setup	UniMF [129]	MVTec 3D-AD	modality used at input: RGB only vs 3-D only vs RGB+3-D fusion, under 1/2/4-shot	standalone 3-D reaches AUPRO 44.7% versus 87.9% for RGB in the 1-shot setting; fused RGB+3D attains I-AUROC 76.9% (1-shot)
Rotation or pose perturbation	3D-ADAM [145]	–	part orientation on a 2-axis fixture during capture	minimum of a full rotation of the primary axis at 45-degree intervals, secondary axis fixed at 0 and 30 degrees
Rotation or pose perturbation	CLIP3D-AD [186]	MVTec3D	Which of the 27 rendered viewpoints (obtained by rotating the point cloud about x/y/z) are selected as the multi-view input set	1-shot I-AUROC 67.4 for views {5,9,19,27}, 71.1 for {3,7,12,17,25}, 72.3 for {5,9,14,18,19,27}, 73.3 for {5,9,14,19,27}; 2-shot 70.3/72.8/72.1/74.0
Rotation or pose perturbation	CoGeoAD [185]	MVTec3D	Number of rendered views Nv (2, 4, 6, 8, 9, 10, 12) at 336 x 336	I-AUROC 81.7 (2 views), 85.1 (4), 84.8 (6), 86.2 (8), 87.4 (9), 85.0 (10), 82.1 (12)
Rotation or pose perturbation	MAD (PAD benchmark) [142]	MAD	query object pose unknown at test time; anomaly-free views rendered over a sphere	OmniposeAD outperforms CFlow by +7.0/+19.6 and UniAD by +8.7/+28.7 in the pose-agnostic setting
Rotation or pose perturbation	PCDiff [184]	AnomalyShapeNet	Elevation range and azimuth view count of the multi-view rendering used by the local branch	O-AUROC 0.93 at elevation [-45,0,45] with 6 azimuth views -> 0.87 at [-60,0,60], 0.86 at [-30,0,30], 0.90 at 8 views, 0.88 at 4 views
Rotation or pose perturbation	PointAD [20]	MVTec3D	rendering angles shifted by 1/20 pi, 2/20 pi, 3/20 pi (printed as 1/15 pi, 2/15 pi, 3/15 pi in the table) while keeping the adjacent-angle discrepancy fixed	point global (82.0, 94.2) at shift 0 -> (82.6, 94.6), (82.1, 94.4), (82.6, 94.6); point local (95.5, 84.4) -> (95.4, 83.9), (95.4, 84.1), (95.4, 84.3)
Rotation or pose perturbation	PointAD+ [63]	MVTec3D	absolute rendering orientation shifted by pi/20, 2pi/20 and 3pi/20 while keeping the adjacent-angle discrepancy of pi/5	local (96.9, 88.7) / global (82.5, 94.8) at shift 0; (96.6, 87.8) / (83.2, 94.9) at pi/20; (96.7, 88.0) / (83.2, 94.9) at 2pi/20; (96.7, 87.9) / (84.1, 95.2) at 3pi/20
Rotation or pose perturbation	Reg2Inv [77]	Real3D	Test point clouds randomly rotated around one of the X, Y or Z axes by 90, 180 or 270 degrees	O-AUROC 76.4 / P-AUROC 83.7 at 0 deg; 76.3/83.9 at 90 deg; 76.2/83.6 at 180 deg; 76.6/84.3 at 270 deg
Rotation or pose perturbation	SPA-SDF [97]	ArtiAD	articulation/joint configuration: test poses drawn from the training pose distribution (seen split) versus novel joint configurations (unseen split)	Obj-AUROC 0.8843 (seen) -> 0.8740 (unseen); Pt-AUROC 0.8651 (seen) -> 0.8410 (unseen)

Condition	Source	Benchmark	What was varied	Reported effect
Input resolution	CoGeoAD [185]	MVTec3D	Rendered image resolution 336 x 336 vs 224 x 224 (at 9 views)	I-AUROC 87.4 -> 75.1, AP 96.5 -> 91.8, P-AUROC 97.5 -> 94.6, AUPRO 91.9 -> 83.3 when resolution drops 336 to 224
Input resolution	DUS-Net [102]	AnomalyShapeNet	Up-Net reconstruction up-sampling rate gamma (1, 2, 4, 6, 8)	O-AUROC 0.620 (gamma=1) to 0.797 (gamma=8); P-AUROC 0.588 to 0.712; FPS 4.4 to 2.1
Input resolution	Eyecandies [141]	Eyecandies	autoencoder input resolution	all inputs "downscaled to a fixed resolution of 256 x 256 pixels"
Input resolution	Eyecandies [141]	Eyecandies	depth-map value range clamped to fixed bounds	RGB-D -> RGB-cD improves image AUROC in most categories (e.g. Peppermint Candy 0.750 -> 0.740 pixel 0.928 -> 0.940; Gummy Bear image 0.594 -> 0.635); with normals only 6/10 categories improve at image level and 2 at pixel level
Input resolution	MAD (PAD benchmark) [142]	MAD	image resolution differs between the simulated and the real split	400x400 (MAD-Sim); 504x378 (MAD-Real)
Input resolution	PointAD [20]	MVTec3D	Gaussian blur with sigma 0, 1, 5, 9 applied to the 2D renderings to simulate degraded rendering quality	point global (82.0, 94.2) at sigma=0 -> (77.6, 92.2) at sigma=9; point local (95.5, 84.4) -> (95.1, 82.3); multimodal global (86.9, 96.1) -> (83.2, 95.0)
Input resolution	PointAD [20]	MVTec3D	point clouds downsampled by farthest point sampling to 70%, 50%, 30%, 20%; PointAD trained and tested at the same resolution	point global (82.0, 94.2) at 100% -> (80.6, 93.3) 70% -> (77.9, 92.6) 50% -> (74.6, 91.1) 30% -> (73.9, 90.9) 20%; point local (95.5, 84.4) -> (94.3, 78.7) at 20%
Input resolution	Real-IAD D3 [152]	MVTec 3D-AD; Real-IAD D3	M3DM feature interpolation removed, on a low- and a high-resolution dataset	MVTec 3D-AD I-AUROC 0.822 -> 0.803 (-0.019), P-AUROC 0.967 -> 0.856 (-0.111); Real-IAD D3 I-AUROC 0.841 -> 0.832 (-0.009), P-AUROC 0.922 -> 0.934 (+0.012)
Input resolution	Real-IAD D3 [152]	Real-IAD D3	point cloud downsampled 4x and 40x to simulate Real3D-AD and MVTec 3D-AD resolutions	"subtle flaws visible at full resolution become hard to detect or vanish entirely at 40x downsampling"
Input resolution	SiM3D [146]	SiM3D	input image/depth resolution reduced from sensor resolution	rendered at sensor resolution 4096 x 3000, then "padded and downsampled to 1540 x 1540"
Input resolution	SiM3D [146]	SiM3D	3D representation fed to multimodal baselines: point clouds vs rendered depth maps	BTF real2real I-AUROC 0.478 -> 0.707 and V-AUPRO 0.444 -> 0.609; CFM 0.448 -> 0.548; M3DM 0.621 -> 0.659
Input resolution	Simple3D [81]	Real3D; AnomalyShapeNet; MulSen-AD; MiniShift	detection/sampling resolution t varied from 256 (2^8) to 8192 (2^13) points, aggregation point count fixed at 16	consistent improvement in all metrics as resolution increases; above 2^12 the method surpasses prior SOTA (per-resolution values are plotted only, not printed)
Input resolution	UCF [128]	own surface-mount inspection dataset	feature-map size (spatial resolution the UCF operates on), including 28x28 and 56×56	I-AUROC and P-AUROC remain above 0.930 and 0.912 except a significant drop at 28x28; at 56×56: 0.942, 0.912, 19.73 G FLOPs
Input resolution	ZUMA [121]	MVTec3D	rendering artifacts simulated by Gaussian blur with parameter lambda = 0, 1, 3, 5	P-AUC 97.6 -> 97.5 (-0.1) at lambda=1, 97.4 (-0.2) at 3, 97.2 (-0.4) at 5; I-AUC 84.0 -> 83.5 (-0.5), 83.1 (-0.9), 82.8 (-1.2)
Lighting	Eyecandies [141]	Eyecandies	six controlled lighting patterns per sample (box lights; each of four camera lights alone; all camera lights)	"while the presence of a defect can appear unclear under some specific lighting conditions, when comparing multiple shots ... the detection becomes much easier"

Condition	Source	Benchmark	What was varied	Reported effect
Lighting	MambaAD3D [89]	RealScan-AD	illumination systematically varied and small pose perturbations introduced during RealScan-AD capture	no per-condition numbers reported
Lighting	PointAD [20]	MVTec3D	rendering lighting set to weakest (–), weaker (-), original, stronger (+) and strongest (++); PointAD trained on the original dataset	point global (82.0, 94.2) original; (82.0, 94.3) at ++, (82.4, 94.6) at +, (82.4, 94.5) at -, (81.9, 94.3) at –; point local (95.5, 84.4) -> (95.3, 83.4) at –
Lighting	PointAD+ [63]	MVTec3D	rendering lighting intensity in Open3D varied over five levels: ++ (strongest), + , original, - , – (weakest)	local (96.9, 88.3) / global (82.1, 94.8) at ++; (96.9, 88.3) / (82.0, 95.1) at +; (96.9, 88.7) / (82.5, 94.8) original; (96.7, 87.9) / (81.8, 94.8) at -; (96.7, 87.7) / (81.5, 94.8) at –
Lighting	R^3-PAD [117]	MAD; PIAD	presence/absence of the Retinex-UNet illumination-alignment module on the categories exhibiting lighting reconstruction discrepancy (MAD) and train-test lighting discrepancy (PIAD)	I-AUROC none -> Retinex-UNet: Gorilla 94.7 -> 96.2, Owl 90.6 -> 95.4, Phoenix 92.0 -> 96.8, Cat 92.5 -> 95.7 (MAD); Amphora 74.8 -> 84.3, Teapot 86.6 -> 91.2 (PIAD)
Lighting	R^3-PAD (Retinex-UNet on PatchCore) [117]	MVTec3D	low-light test subset of MVTec 3D-AD: the bottom 50% of each category's test images by Grayscale Mean Intensity, training set unchanged	I-AUROC none -> Retinex-UNet: Cable gland 64.0 -> 77.5, Dowels 69.3 -> 79.8, Foam 71.5 -> 83.4, Ropes 77.8 -> 89.2; P-AUROC 81.4 -> 86.7, 78.0 -> 85.3, 85.0 -> 88.5, 89.3 -> 92.2
Lighting	ReflectSL [148]	self-built reflective-surface dataset	Projection strategy for highly reflective surfaces: conventional uniform projection vs multi-exposure fusion vs polarization-based imaging vs the proposed adaptive projection	Reconstruction integrity 82.50% (traditional, 120 ms) -> 97.39% (multi-exposure fusion, 480 ms) -> 97.30% (polarization-based, 120 ms) -> 98.67% (proposed, 390 ms)
Lighting	ZUMA [121]	MVTec3D	photometric conditions of the renderings: + +, +, Org, -, - -	P-AUC 97.6 (Org) -> 97.7 (+0.1) at ++, 97.6 (0.0) at +, 97.7 (+0.1) at -, 97.5 (-0.1) at –; I-AUC 84.0 -> 83.8, 83.7, 84.0, 83.9

Reference numbers in this document are those of the article's reference list.
